# Linear Stability of Schwarzschild–Anti-de Sitter Spacetimes III: Quasimodes and Sharp Decay of Gravitational Perturbations

**DOI:** 10.1007/s00220-025-05453-9

**Published:** 2025-10-03

**Authors:** Olivier Graf, Gustav Holzegel

**Affiliations:** 1 Univ. Grenoble Alpes, CNRS, IF, 38000 Grenoble, France; 2 Mathematisches Institut, Universität Münster, Einsteinstrasse 62, 48149 Münster, Federal Republic of Germany; 3 Department of Mathematics, Imperial College London, South Kensington Campus, London, SW7 2AZ UK

## Abstract

In this last part of the series we prove that the slow (inverse logarithmic) decay in time of solutions to the linearised Einstein equations on Schwarzschild–Anti-de Sitter backgrounds obtained in Graf and Holzegel (Linear stability of Schwarzschild–Anti-de sitter spacetimes I: the system of gravitational perturbations. arXiv:2408.02251, 2024) and Graf and Holzegel (Linear stability of Schwarzschild–Anti-de sitter spacetimes II: logarithmic decay of solutions to the Teukolsky system. arXiv:2408.02252, 2024) is in fact optimal by constructing quasimode solutions for the Teukolsky system. The main difficulties compared with the case of the scalar wave equation treated in earlier works arise from the first order terms in the Teukolsky equation, the coupling of the Teukolsky quantities at the conformal boundary and ensuring that the relevant quasimode solutions satisfy the Teukolsky–Starobinsky relations. The proof invokes a quasimode construction for the corresponding Regge–Wheeler system (which can be fully decoupled at the expense of a higher order boundary condition) and a reverse Chandrasekhar transformation which generates solutions of the Teukolsky system from solutions of the Regge–Wheeler system. Finally, we provide a general discussion of the well-posedness theory for the higher order boundary conditions that typically appear in the process of decoupling.

## Introduction

This is the third and final paper of our series studying the time evolution of gravitational perturbations on the black hole exterior of a Schwarzschild–Anti-de Sitter (Schwarzschild-AdS) spacetime. In Part 1 [GH24a], the authors established the linear stability of the Schwarzschild-AdS spacetime by showing that in a suitably (initial data) normalised double null gauge, all linearised geometric quantities decay inverse logarithmically in time to a member of the 4-parameter family of linearised Kerr-AdS solutions, the latter being identifiable directly at the level of initial data. A key ingredient of the proof in [GH24a], established independently in Part 2 of the series [GH24b], was to prove inverse logarithmic decay rates for the pair of (linearised) extremal null curvature quantities $\alpha ^{[\pm 2]}$, which are spin-weighted, complex valued functions on the Schwarzschild-AdS manifold, also known as the Teukolsky variables.

### The Teukolsky system and informal statement of the main result

We recall that the linearised Einstein equations imply decoupled equations for the aforementioned Teukolsky variables $\alpha ^{[\pm 2]}$. For convenience, we collect them here in the standard $(t,r,\vartheta , \varphi )$ coordinates on the Schwarzschild-AdS manifold $(\mathcal {M},g_{M,k})$ (see (2.2) below):

1.1a $$\begin{aligned} \begin{aligned} 0&= \Box _{g_{M,k}}\alpha ^{[+2]} + \frac{2}{r^2}\frac{{\textrm{d}}\Delta }{{\textrm{d}}r}{\partial }_r\alpha ^{[+2]} + \frac{4}{r^2}\left(\frac{r^2}{2\Delta }\frac{{\textrm{d}}\Delta }{{\textrm{d}}r}-2r\right){\partial }_t\alpha ^{[+2]} + \frac{4}{r^2}i\frac{\cos {\vartheta }}{\sin ^2{\vartheta }}{\partial }_\varphi \alpha ^{[+2]}\\&\quad + \frac{2}{r^2}\left(1+15k^2r^2-2\cot ^2{\vartheta }\right)\alpha ^{[+2]}, \end{aligned} \end{aligned}$$

and

1.1b $$\begin{aligned} \begin{aligned} 0&= \Box _{g_{M,k}}\alpha ^{[-2]} - \frac{2}{r^2}\frac{{\textrm{d}}\Delta }{{\textrm{d}}r}{\partial }_r\alpha ^{[-2]} - \frac{4}{r^2}\left(\frac{r^2}{2\Delta }\frac{{\textrm{d}}\Delta }{{\textrm{d}}r}-2r\right){\partial }_t\alpha ^{[-2]} - \frac{4}{r^2}i\frac{\cos {\vartheta }}{\sin ^2{\vartheta }}{\partial }_\varphi \alpha ^{[-2]}\\&\quad + \frac{2}{r^2}\left(-1+3k^2r^2-2\cot ^2{\vartheta }\right)\alpha ^{[-2]}, \end{aligned} \end{aligned}$$

where $\Delta := r^2 + k^2 r^4 - 2Mr$, and $\Box _{g_{M,k}}$ is the d’Alembertian operator associated with the metric $g_{{M,k}}$,

$$\begin{aligned} \Box _{g_{M,k}}&= -\frac{r^2}{\Delta }{\partial }_t^2 + \frac{1}{r^2}{\partial }_r\left(\Delta {\partial }_r\right) + \frac{1}{r^2\sin {\vartheta }}{\partial }_{\vartheta }\left(\sin {\vartheta }{\partial }_{\vartheta }\right) + \frac{1}{r^2\sin ^2{\vartheta }}{\partial }_\varphi ^2. \end{aligned}$$

We recall also (see [GH24a] for a derivation) that fixing the conformal class of the non-linear metric on the conformal boundary for the perturbations determines the following coupled *conformal boundary conditions at infinity* for the $\alpha ^{[\pm 2]}$ (with the $*$ denoting complex conjugation):

1.2a $$\begin{aligned} \widetilde{\alpha }^{[+2]} - \left( \widetilde{\alpha }^{[-2]}\right) ^*&\xrightarrow {r\rightarrow +\infty } 0, \end{aligned}$$

1.2b $$\begin{aligned} r^2{\partial }_r\widetilde{\alpha }^{[+2]} + r^2{\partial }_r\big (\widetilde{\alpha }^{[-2]}\big )^*&\xrightarrow {r\rightarrow +\infty } 0, \end{aligned}$$

where $\widetilde{\alpha }^{[+2]} = \Delta ^2r^{-3}\alpha ^{[+2]}$ and $\widetilde{\alpha }^{[-2]}:= r^{-3}\alpha ^{[-2]}$. The main result of [GH24b] can then be informally stated as

#### Theorem 1.1

([GH24b]) Solutions to the system (1.1) with the boundary conditions (1.2) arising from suitably regular initial data prescribed on a spacelike hypersurface connecting the future event horizon $\mathcal {H}^+$ with the conformal boundary $\mathcal {I}$ decay (at least) inverse logarithmically in time.

We emphasise that Theorem 1.1 holds independently of the full system of linearised Einstein equations (“the system of gravitational perturbations”) considered in [GH24a]. On the other hand, it is of course natural to ask whether the solutions to the Teukolsky system in Theorem 1.1 always generate solutions to the system of gravitational perturbations. It turns out (as proven in [GH24a]) that a necessary and sufficient condition is that $\alpha ^{[\pm 2]}$ are in addition related by the Teukolsky–Starobinsky identities, which one should think of as constraints arising from the validity of the full system gravitational perturbations.^1^

#### Definition 1.2

We say that $\widetilde{\alpha }^{[\pm 2]}$ satisfy the *Teukolsky–Starobinsky identities* if

1.3a $$\begin{aligned} w^2(w^{-1}L)^4(\widetilde{\alpha }^{[-2]})^*&= \mathcal {L}_{-1}\mathcal {L}_{0}\mathcal {L}_{1}\mathcal {L}_{2}(\widetilde{\alpha }^{[+2]})^*- 12M{\partial }_t\widetilde{\alpha }^{[+2]}, \end{aligned}$$

1.3b $$\begin{aligned} w^2(w^{-1}{\,\underline{L}})^4(\widetilde{\alpha }^{[+2]})^*&= \mathcal {L}_{-1}^{\dagger }\mathcal {L}_{0}^{\dagger }\mathcal {L}_{1}^{\dagger }\mathcal {L}_{2}^{\dagger }(\widetilde{\alpha }^{[-2]})^*+ 12M{\partial }_t\widetilde{\alpha }^{[-2]}, \end{aligned}$$

where $L=\partial _t + \frac{\Delta }{r^2} \partial _r$ and $\underline{L} = \partial _t - \frac{\Delta }{r^2} \partial _r$ are the null directions in Schwarzschild-AdS and for all $n\in \mathbb {Z}$

1.4 $$\begin{aligned} \mathcal {L}_{n}&:= \left({\partial }_{\vartheta }+ \frac{i}{\sin {\vartheta }} {\partial }_\varphi \right) + n \cot {\vartheta },&\mathcal {L}_{n}^{\dagger }&:= \left({\partial }_{\vartheta }- \frac{i}{\sin {\vartheta }} {\partial }_\varphi \right) + n \cot {\vartheta }. \end{aligned}$$

#### Remark 1.3

One easily checks that (1.3) are indeed equivalent to the tensorial Teukolsky–Starobinsky identities for  given in Section 2.10 of [GH24a]. See Sect. 2.8 below for details.

#### Theorem 1.4

(Producing full solutions from solutions to Teukolsky system; Proposition 3.13 of [GH24a]). Let $\widetilde{\alpha }^{[\pm 2]}$ be solutions to the system (1.1) with the boundary conditions (1.2) arising from suitably regular initial data and satisfying in addition the Teukolsky–Starobinsky identities (1.3). Then, there exists a solution to the system of gravitational perturbations on Schwarzschild-AdS (regular at the horizon and conformal at infinity to the AdS metric, see [GH24a]), such that its associated Teukolsky quantities coincide with $\widetilde{\alpha }^{[\pm 2]}$.

While the validity of the Teukolsky–Starobinsky identities did not have to be used in [GH24a] (having proven the upper bound of Theorem 1.1 for the *larger* class of solutions to the Teukolsky system in [GH24b] was more than sufficient in [GH24a]) it becomes relevant when we want to establish *lower bounds* in the setting of Theorem 1.1. In particular, we would like to construct solutions with very slow decay *which in addition satisfy the Teukolsky–Starobinsky identities* because it is only in this way that our lower bounds for the Teukolsky system have relevance for the system of gravitational perturbations. Our main result can be informally stated as follows (see already Theorem 3.1 in Sect. 3 for the formal statement):

#### Theorem 1.5

General solutions to the system (1.1) with the boundary conditions (1.2) arising from suitably regular initial data prescribed on a spacelike hypersurface connecting the future event horizon $\mathcal {H}^+$ with the conformal boundary $\mathcal {I}$ and satisfying in addition the Teukolsky–Starobinsky identities cannot decay better than inverse logarithmically in time.

Note that this does not exclude solutions exhibiting faster decay but only that if one insists on a uniform decay bound for *all* solutions, then the optimal rate is inverse logarithmic.^2^ Note also that it is through Theorem 1.4 that we can interpret Theorem 1.5 as showing that inverse logarithmic decay is optimal for the system of gravitational perturbations.

The proof of Theorem 1.5 proceeds by the construction of special solutions to the Teukolsky system that are as close as possible to *quasimodes*, *i.e.* to time-periodic (=non-decaying) approximate solutions of the Teukolsky system. In fact, one first constructs quasimodes by solving an eigenvalue problem for a truncated Teukolsky system and then perturbs them (with exponentially small errors) to actual slowly decaying solutions. We outline the details of the proof in Sect. 1.3 but first compare with the case of the scalar wave equation, where such a construction has already been carried out successfully in [HS14, Gan14].

### Comparison with the linear wave equation and main difficulties

About a decade ago, the second author in collaboration with J. Smulevici established exact analogues of Theorems 1.1 and 1.5 for the massive wave equation, $\Box _g \psi + \alpha \psi =0$, with Dirichlet conditions imposed on $\psi$ on the more complicated Kerr-AdS black hole exterior.^3^ While the background geometry considered in the present paper is simpler, there are nevertheless several novel analytical difficulties:

1. The (large) first order terms appearing in the Teukolsky equation (1.1). Indeed, the proof in [HS14] relied on separation of variables and, for the quasimode construction, the fact that the resulting one-dimensional operator was *self-adjoint*. This is not true for the Teukolsky equation (1.1).
2. The coupling of the two Eq. (1.1) through the boundary condition (1.2).
3. The additional requirement that the generalised quasimode construction has to ensure validity of the Teukolsky–Starobinsky identities as described in Sect. 1.1.

### Overview and main ideas of the proof

To address the first difficulty above, one exploits a remarkable algebraic relation between solutions to the Teukolsky equations and solutions to the Regge–Wheeler equations first explored in the asymptotically flat context (and for mode solutions) by Chandrasekhar. We briefly review this algebraic structure in Sect. 1.3.1.

#### Chandrasekhar transformations and Reverse Chandrasekhar Transformations

Let $w=\frac{\Delta }{r^2}$. For a pair of spin-weighted functions $\widetilde{\alpha }^{[\pm 2]}$ one defines the *Chandrasekhar transformations* of $\widetilde{\alpha }^{[\pm 2]}$ to be the functions

1.5a $$\begin{aligned} \psi ^{[+2]}&:= w^{-1}{\,\underline{L}}\widetilde{\alpha }^{[+2]},&\Psi ^{[+2]}&:= w^{-1}{\,\underline{L}}\psi ^{[+2]}, \end{aligned}$$

1.5b $$\begin{aligned} \psi ^{[-2]}&:= w^{-1}L\widetilde{\alpha }^{[-2]},&\Psi ^{[-2]}&:= w^{-1}L\psi ^{[-2]}. \end{aligned}$$

The point is that if $\alpha ^{[\pm 2]}$ satisfy the Teukolsky equations, then the $\Psi ^{[\pm 2]}$ constructed from $\widetilde{\alpha }^{[\pm 2]}$ satisfy the following *Regge–Wheeler equations* (see Proposition 2.2; the angular operators $\mathcal {L}^{[\pm 2]}$ are defined in (2.6))

1.6 $$\begin{aligned} \begin{aligned} 0&= -L{\,\underline{L}}\Psi ^{[\pm 2]} - \frac{\Delta }{r^4}\left(\mathcal {L}^{[\pm 2]}-\frac{6M}{r}\right)\Psi ^{[\pm 2]} =: \mathfrak {R}^{[\pm 2]}\Psi ^{[\pm 2]}. \end{aligned} \end{aligned}$$

Moreover, one can show that the $\Psi ^{[\pm 2]}$ obey the following boundary conditions:

1.7a $$\begin{aligned} \Psi ^{[+2]}-(\Psi ^{[-2]})^*&\xrightarrow {r\rightarrow +\infty } 0, \end{aligned}$$

1.7b $$\begin{aligned} LL\Psi ^{[+2]} + {\,\underline{L}}{\,\underline{L}}(\Psi ^{[-2]})^*+ \frac{1}{6M}\mathcal {L}\left(\mathcal {L}-2\right)\left(L\Psi ^{[+2]} - {\,\underline{L}}(\Psi ^{[-2]})^*\right)&\xrightarrow {r\rightarrow +\infty } 0, \end{aligned}$$

where $\mathcal {L}:= \mathcal {L}^{[+2]}$ (note that $\big (\mathcal {L}^{[-2]}\Psi \big )^*= \mathcal {L}^{[+2]}\Psi ^*$). We observe that the mixed Neumann condition for the Teukolsky variables (1.2b) has been replaced by a mixed higher order boundary condition. One can also derive a first order mixed Neumann boundary condition (see (1.12) in [GH24b]), however that condition couples back to the $\widetilde{\alpha }^{[\pm 2]}$ themselves and is inconvenient when trying to analyse (1.6) by itself.

In this paper we also define the following *reverse Chandrasekhar transformations*. For a pair of spin-weighted functions $\Psi ^{[\pm 2]}$ we define

1.8a $$\begin{aligned} \psi ^{[+2],r}&:= w^{-1}L\Psi ^{[+2]}, \nonumber \\ \widetilde{\alpha }^{[+2],r}&:= w^2\left(\mathcal {L}^{[+2]}(\mathcal {L}^{[+2]}-2)+12M{\partial }_t\right)\left(w^{-1}L\right)\psi ^{[+2],r}, \end{aligned}$$

1.8b $$\begin{aligned} \psi ^{[-2],r}&:= w^{-1}{\,\underline{L}}\Psi ^{[-2]},\nonumber \\ \widetilde{\alpha }^{[-2],r}&:= w^2\left(\mathcal {L}^{[-2]}(\mathcal {L}^{[-2]}-2)-12M{\partial }_t\right)\left(w^{-1}{\,\underline{L}}\right)\psi ^{[-2],r}. \end{aligned}$$

The point is that given solutions $\Psi ^{[\pm 2]}$ to the Regge–Wheeler equations (1.6) the pair $\widetilde{\alpha }^{[\pm 2],r}$ defined above will satisfy the Teukolsky equations (2.5). Moreover, if the $\Psi ^{[\pm 2]}$ satisfy the Regge–Wheeler boundary conditions (1.7), $\widetilde{\alpha }^{[\pm 2,r]}$ will satisfy the Teukolsky boundary conditions (1.2).

We remark already that the (reverse) Chandrasekhar transformations are not injective without imposing further conditions. Their kernels can be understood explicitly. However, as this is not necessary for the constructions in this paper, we will not comment on this further here.

#### Quasimodes for the Regge–Wheeler problem

The algebraic structure revealed in Sect. 1.3.1 suggests a strategy to overcome the first difficulty. Since the Regge–Wheeler equation (1.6) does not have first order terms, we can—by its close analogy with the wave equation itself—repeat the construction of quasimodes for the Regge–Wheeler equation as done for the scalar wave equation in [HS14]. We give a very short discussion referring the reader to the introduction of [HS14] for details. See also Sect. 4.2 below. The idea is to study the semi-classical Schrödinger-type problem

1.9 $$\begin{aligned} - \partial _{r^\star }^2 \Psi _\ell + \frac{\Delta }{r^4} \left( \ell \left( \ell + 1\right) - \frac{6M}{r}\right) \Psi _\ell = \omega ^2 \Psi _\ell \end{aligned}$$

for the angular separated Regge–Wheeler equation. One exploits the maximum of the potential *V* at $r=3M$ to construct bound states in $r \ge 3M$ (by putting a Dirichlet condition at $r=3M$) and cuts these off (near $r=3M$) to solutions defined on all of $[r_+,\infty )$. The error from the cut-off is exponential small in the angular momentum number $\ell$ by a classical Agmon estimate. In summary, the final objects $\Psi _\ell$ thus constructed are approximate solutions to the Regge–Wheeler equation in the sense that they solve the equation everywhere except in a small region near $r=3M$ and we have

1.10 $$\begin{aligned} \big \Vert \Psi _\ell \big \Vert _{H^n(\Sigma _{t^\star }\cap \{3M\le r \le 3M+\delta \})} \le Ce^{-C^{-1}\ell }\big \Vert \Psi _\ell \big \Vert _{H^1(\Sigma _{t^\star _0})} \end{aligned}$$

Such quasimodes for the Regge–Wheeler system are then transformed to quasimodes for the Teukolsky system using the reverse Chandrasekhar transformation (1.8) with analogous control on the error. As in [HS14], an application of Duhamel’s principle produces the required candidate solutions for the desired lower bound (3.1), which implies that solutions cannot decay faster than inverse logarithmically.

#### Ensuring the boundary conditions and the Teukolsky–Starobinsky identities

In our discussion we have so-far ignored the fact that we need to perform the construction of Sect. 1.3.2 ensuring the complicated mixed higher order boundary condition (1.7) at the conformal boundary for the Regge–Wheeler quantities. (In [HS14] considerations were applied to a scalar with Dirichlet conditions.) We have also not addressed whether and which additional constraints should enter the construction of the $\Psi ^{[\pm 2]}$ to ensure that the corresponding Teukolsky quantities $\alpha ^{[\pm 2]}$ satisfy the Teukolsky–Starobinsky identities.

To address the first point one introduces new quantities (the notation $\frac{1}{\mathcal {L}}$ denoting the inverse of $\mathcal {L}$)

1.11 $$\begin{aligned}&\Psi ^D = \Psi ^{[+2]} - \big (\Psi ^{[-2]}\big )^*\ \ \ \text {and} \nonumber \\&\Psi ^R = \left(\Psi ^{[+2]} + \big (\Psi ^{[-2]}\big )^*\right)+\frac{12M}{\mathcal {L}(\mathcal {L}-2)}{\partial }_t\left(\Psi ^{[+2]} - \big (\Psi ^{[-2]}\big )^*\right), \end{aligned}$$

which also satisfy the Regge–Wheeler equation (1.6) but decoupled (albeit still higher order) boundary conditions

1.12 $$\begin{aligned} \Psi ^D \xrightarrow {r\rightarrow +\infty } 0 \ \ \ \text {and} \ \ \ 2{\partial }_{t}^2\Psi ^R + \frac{\mathcal {L}(\mathcal {L}-2)}{6M}{\partial }_{r^\star }\Psi ^R + k^2\mathcal {L}\Psi ^R \xrightarrow {r\rightarrow +\infty } 0 \, . \end{aligned}$$

To address the second point, we establish—by a sequence of algebraic manipulations—that a necessary and sufficient condition for $\Psi ^D$ and $\Psi ^R$ to generate $\widetilde{\alpha }^{[\pm 2]}$ satisfying the Teukolsky–Starobinsky relations is that $\Psi ^D_s=\Psi ^D + (\mathcal {C} \Psi ^D)^\star =0$ and $\Psi ^R_a=\Psi ^R - (\mathcal {C} \Psi ^R)^\star =0$, where $\mathcal {C}$ is the conjugacy operator defined in Definition 2.16.

The main difficulty with performing the construction outlined in Sect. 1.3.2 for $\Psi ^R$ then is that (1.9) has to be solved with the higher order boundary condition (1.12) at infinity (which, as it contains *T*-derivatives, involves itself the eigenvalue $\omega$ we are looking for) and to combine modes such that $\Psi ^R$ and $\Psi ^D$ satisfy $\Psi ^R_a=0$ and $\Psi ^D_s=0$. Specifically, the construction requires a well-posedness statement for the Regge–Wheeler equation with the higher-order boundary condition (1.12) for $\Psi ^R$, which we provide in Sect. 4.4.

### Final comments

The inverse logarithmic decay of solutions to wave-type equations being sharp is more generally related to the existence of stable trapped light rays, see for example [Ral71]. As mentioned above, for the wave equation on Anti-de Sitter black holes, this was first shown in [HS14] and [Gan14]. See also [Ben21] for the case of black strings and black rings. The general idea is that the existence of stable trapped rays can be used to construct quasimodes, see [Ral76, de77] and [Zwo12, DZ19] for general discussions.

For black holes it is well-known that the existence of quasimodes and the associated slow inverse logarithmic decay is closely related to the behaviour of quasinormal modes, more specifically the existence of an exponentially small resonance free region near the axis. We refer to [War15] for a general definition of quasinormal modes in the asymptotically AdS black hole setting and to the papers [Gan14, Gan17] for a construction of quasinormal modes from quasimodes, which can in particular be applied to the quasimodes constructed for the wave equation on Kerr-AdS in [HS14]. One may expect that these techniques (combined with the ones in this paper) can be applied to obtain analogous results on quasinormal modes for the Teukolsky system discussed here.

For a numerical computation of quasinormal modes for the Teukolsky system in the more complicated Kerr-AdS case see [DS13, CDH14]. In the latter papers, decoupled (but higher order) boundary conditions are derived individually for $\alpha ^{[\pm 2]}$ themselves assuming they satisfy the Teukolsky–Starobinsky constraints.^4^ It is a natural question whether these higher order boundary conditions are well-posed. In Appendix B we provide a way to construct solutions with these boundary conditions directly. However, the proof for $\alpha ^{[-2]}$ (say) goes through constructing auxiliary data for a $\alpha ^{[+2]}$ from the Teukolsky–Starobinsky relations and applying the usual well-posedness result for the coupled system to construct the evolution of $\alpha ^{[-2]}$—illustrating the fundamental role of the coupled system. We note that the situation is different for the higher order boundary condition (1.12) satisfied by the $\Psi ^R$, as this leads to an actual energy estimate for $\Psi ^R$ (exploited crucially in [GH24b]), which is the underlying reason the non-standard eigenvalue problem for $\Psi ^R$ discussed above is treatable here.

We finally comment on Theorem 1.4. It was obtained in [GH24a, Proposition 3.13] by directly integrating the system of gravitational perturbations in double null gauge. For readers unfamiliar with the double null gauge, we present in Appendix A an alternative way to construct a metric perturbation from a solution of the Teukolsky system based on duality (see [Wal78] and references therein). While this allows displaying explicit formulae for (most of) the components of the system of gravitational perturbations, the Teukolsky quantities associated with this metric perturbation are *not* the original ones but only related to them by a Teukolsky–Starobinsky transformation. Understanding this transformation (on quasimodes) is then sufficient to also interpret Theorem 1.5 as providing an optimal decay for the system of gravitational perturbations without invoking Theorem 1.4.

## Preliminaries

We collect the necessary preliminaries to make the paper self-contained referring readers to [GH24b] for details.

### The Schwarzschild-AdS background

We fix $k>0$ and define the manifold $\mathcal {M}:=(-\infty ,\infty )_{t^\star } \times [r_+,\infty )_r \times {\mathbb S}^2$, where $r=r_+$ is defined as the largest real zero of $\Delta := r^2 + k^2 r^4 - 2Mr$, and equip it with the metric

2.1 $$\begin{aligned} g_{M,k}&= -\left( 1 + k^2 r^2 -\frac{2M}{r}\right) ({\textrm{d}}t^\star )^2 + \frac{4M}{r(1+k^2 r^2)} {\textrm{d}}t^\star {\textrm{d}}r +\frac{1 + k^2 r^2 +\frac{2M}{r}}{(1+k^2 r^2)^2} {\textrm{d}}r^2 \nonumber \\&\quad +r^2\left({\textrm{d}}{\vartheta }^2+\sin ^2{\vartheta }{\textrm{d}}\varphi ^2\right) . \end{aligned}$$

**Fig. 1:**
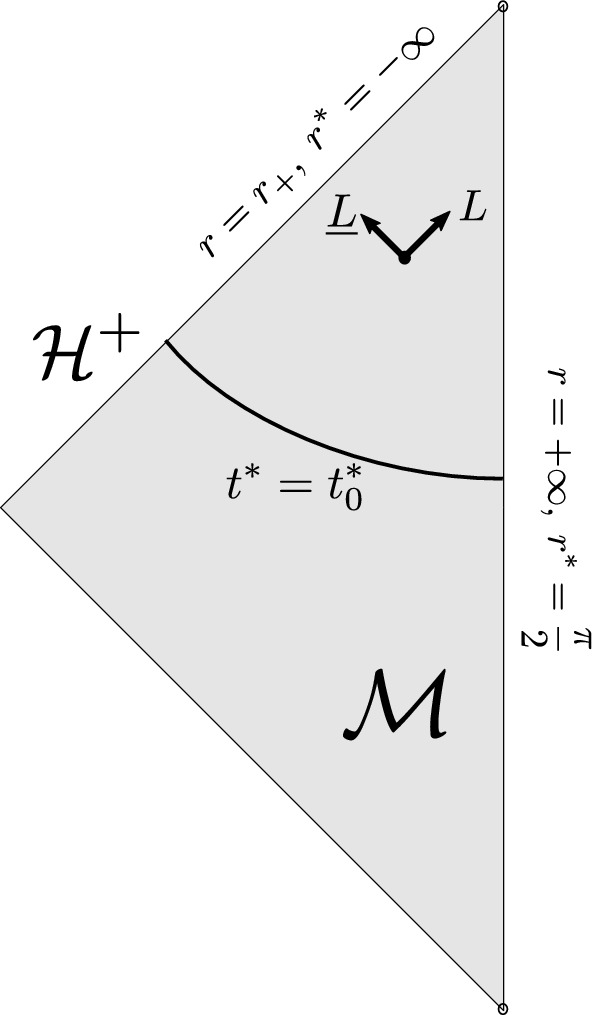
Penrose diagram of Schwarzschild-AdS spacetime $(\mathcal {M}, g_{M,k})$

We call the pair $(\mathcal {M}, g_{M,k})$ (the exterior of) the Schwarzschild-AdS spacetime, which is the unique spherically symmetric solution of $\textrm{Ric}(g) = - \frac{k^2}{3}g$. We denote by $\mathcal {H}^+$ the set $\{ r= r_+\}$, which defines a null boundary of $(\mathcal {M}, g_{M,k})$ referred to as the *future event horizon*. See the Penrose diagram in Fig. 1 below for a depiction of the geometry. Note that defining the *radial tortoise coordinate* $r^\star$ and the time coordinate *t* by

$$\begin{aligned} \frac{{\textrm{d}}r^\star }{{\textrm{d}}r}&:= \frac{r^2}{\Delta },&r^\star \left(r=+\infty \right)&= \frac{\pi }{2},&t&:= t^\star - r^\star + \frac{1}{k}\arctan (k r) \, , \end{aligned}$$

we obtain the more familiar Schwarzschildean form of the metric,

2.2 $$\begin{aligned} g_{M,k} = \!-\!\left( 1+ k^2r^2 -\frac{2M}{r} \right) {\textrm{d}}t^2 +\left( 1+k^2r^2-\frac{2M}{r}\right) ^{-1} {\textrm{d}}r^2 +r^2\left({\textrm{d}}{\vartheta }^2\!+\!\sin ^2{\vartheta }{\textrm{d}}\varphi ^2\right), \end{aligned}$$

which is well-defined on the interior of $\mathcal {M}$.

We recall the pair of future directed null vectorfields $L,{\,\underline{L}}$, expressed in the above $(t,r,{\vartheta },\varphi )$ coordinates, by

2.3 $$\begin{aligned} L := {\partial }_t+\frac{\Delta }{r^2}{\partial }_r , \qquad \qquad \qquad {\,\underline{L}}:= {\partial }_t-\frac{\Delta }{r^2}{\partial }_r \, . \end{aligned}$$

Using the regular $(t^\star ,r,\theta ,\phi )$ coordinates one infers that *L*, $\Delta ^{-1} \underline{L}$ extend regularly to the boundary $\mathcal {H}^+$ of $\mathcal {M}$.

### Regularity of spin-weighted functions on $\mathcal {M}$

The spin-weighted functions considered in this paper will always be smooth on $\mathcal {M}\setminus \mathcal {H}^+$ (or this set intersected with $\{t^\star \ge t^\star _0\}$). We recall (see for instance equation (28) of [DHR19a]) the analogue of the angular momentum vectorfields for spin *s*-weighted functions:

2.4 $$\begin{aligned} \tilde{Z}_1&= -\sin \phi \partial _\theta + \cos \phi (-is \csc \theta -\cot \theta \partial _\phi ) \ \ , \nonumber \\ \tilde{Z}_2&= -\cos \phi \partial _\theta - \sin \phi (-is \csc \theta -\cot \theta \partial _\phi ) \ \ , \ \ \tilde{Z}_3 = \partial _\phi . \end{aligned}$$

We will call a spin-weighted function $\phi$ of weight $s=\pm 2$ on $\mathcal {M}\setminus \mathcal {H}^+$ *regular at the (future event) horizon* if

$$ L^p (\Delta ^{-1} \underline{L})^q (\tilde{Z}_1)^{k_1} (\tilde{Z}_2)^{k_2} (\tilde{Z}_3)^{k_3} \phi $$

extends continuously to the future event horizon $\mathcal {H}^+$ for all $p,q,k_1,k_2,k_3 \in \mathbb {N}$.

Moreover, we will call $\phi$ *regular at the conformal boundary* (or shortly *regular at infinity*) if

$$ r^2(L-\underline{L})^p (L+\underline{L})^q (\tilde{Z}_1)^{k_1} (\tilde{Z}_2)^{k_2} (\tilde{Z}_3)^{k_3} \phi $$

extends continuously to the conformal boundary $\mathcal {I}$ for all $p,q, k_1, k_2,k_3 \in \mathbb {N}$.

We will sometimes apply the above definitions restricted to $(\mathcal {M} \setminus \mathcal {H}^+) \cap \{t^\star \ge t^\star _0\}$.

### The Teukolsky equations

We recall the quantities

$$\begin{aligned} \widetilde{\alpha }^{[+2]} = \Delta ^2r^{-3}\alpha ^{[+2]}, \quad \widetilde{\alpha }^{[-2]} = r^{-3}\alpha ^{[-2]}, \end{aligned}$$

defined in the introduction. A computation reveals that for $\widetilde{\alpha }^{[+2]},\widetilde{\alpha }^{[-2]}$, the Teukolsky equations (1.1) become

2.5a $$\begin{aligned} 0&= -L{\,\underline{L}}\widetilde{\alpha }^{[+2]} + 2\frac{\Delta }{r^2}{\partial }_r\left(\log \frac{\Delta }{r^4}\right){\,\underline{L}}\widetilde{\alpha }^{[+2]} - \frac{\Delta }{r^4}\left(\mathcal {L}^{[+2]} -2 + \frac{6M}{r}\right) \widetilde{\alpha }^{[+2]} \nonumber \\&=: \mathfrak {T}^{[+2]}\widetilde{\alpha }^{[+2]}, \end{aligned}$$

2.5b $$\begin{aligned} 0&= -L{\,\underline{L}}\widetilde{\alpha }^{[-2]} - 2\frac{\Delta }{r^2}{\partial }_r\left(\log \frac{\Delta }{r^4}\right)L \widetilde{\alpha }^{[-2]} \nonumber \\&\quad - \frac{\Delta }{r^4}\left(\mathcal {L}^{[-2]} -2 + \frac{6M}{r}\right) \widetilde{\alpha }^{[-2]} =: \mathfrak {T}^{[-2]}\widetilde{\alpha }^{[-2]}, \end{aligned}$$

where the angular operators $\mathcal {L}^{[\pm 2]}$ are defined by

2.6 $$\begin{aligned} -\mathcal {L}^{[\pm 2]}&:= \frac{1}{\sin {\vartheta }}{\partial }_{\vartheta }\left(\sin {\vartheta }\,{\partial }_{\vartheta }\right) + \frac{1}{\sin ^2{\vartheta }}{\partial }_\varphi ^2 + 2(\pm 2)i\frac{\cos {\vartheta }}{\sin ^2{\vartheta }}{\partial }_\varphi - 4\cot ^2{\vartheta }-4. \end{aligned}$$

The well-posedness theory (see Theorem 1.6 in [GH24a]) constructs smooth solutions to (2.5) satisfying the boundary conditions (1.2) from smooth initial data prescribed on some spacelike slice $\{t^\star =t^\star _0\}$. Moreover, these solutions are such that $\widetilde{\alpha }^{[+2]}$ and $\Delta ^{-2}\widetilde{\alpha }^{[-2]}$ are regular at the future event horizon and $\widetilde{\alpha }^{[+2]}$ and $\widetilde{\alpha }^{[-2]}$ are regular at the conformal boundary. Hence in particular, the boundary conditions (1.2) make sense.

#### Definition 2.1

We will call $\widetilde{\alpha }^{[\pm 2]}$ solutions of the Teukolsky problem on $\mathcal {M} \cap \{ t^\star \ge t^\star _0\}$ if $\widetilde{\alpha }^{[+2]},\Delta ^{-2}\widetilde{\alpha }^{[-2]}$ are regular at the future event horizon $\mathcal {H}^+$ and $\widetilde{\alpha }^{[+2]},\widetilde{\alpha }^{[-2]}$ are regular at infinity (see Sect. 2.2), and if the $\widetilde{\alpha }^{[\pm 2]}$ satisfy the Teukolsky system (2.5) together with the boundary conditions (1.2).

### Chandrasekhar transformations

We now discuss the transformation theory mapping solutions to the Teukolsky system to the Regge–Wheeler system. The conventions of Sect. 2.2 apply. We denote by $\mathfrak {F}^{[\pm 2]}$ a (smooth) spin-weighted function.

#### Proposition 2.2

Assume that $\widetilde{\alpha }^{[\pm 2]}$ satisfy the inhomogeneous Teukolsky equations

2.7 $$\begin{aligned} \mathfrak {T}^{[\pm 2]}\widetilde{\alpha }^{[\pm 2]}&= \mathfrak {F}^{[\pm 2]}, \end{aligned}$$

Then, the following inhomogeneous Regge–Wheeler equations are satisfied

2.8 $$\begin{aligned}&\begin{aligned} \mathfrak {R}^{[+2]}\widetilde{\alpha }^{[+2]} = \mathfrak {F}^{[+2]} - 2w'\psi ^{[+2]} - w\left(2-\frac{12M}{r}\right)\widetilde{\alpha }^{[+2]}, \\ \mathfrak {R}^{[-2]}\widetilde{\alpha }^{[-2]} = \mathfrak {F}^{[-2]} + 2w'\psi ^{[-2]} - w\left(2-\frac{12M}{r}\right)\widetilde{\alpha }^{[-2]}, \end{aligned} \end{aligned}$$

2.9 $$\begin{aligned}&\begin{aligned} \mathfrak {R}^{[+2]}\psi ^{[+2]} = {\,\underline{L}}\left(w^{-1}\mathfrak {F}^{[+2]}\right) - w'\Psi ^{[+2]} + 6Mw\widetilde{\alpha }^{[+2]}, \\ \mathfrak {R}^{[-2]}\psi ^{[-2]} = L \left(w^{-1}\mathfrak {F}^{[-2]}\right) + w'\Psi ^{[-2]} - 6Mw\widetilde{\alpha }^{[-2]}, \end{aligned} \end{aligned}$$

2.10 $$\begin{aligned}&\begin{aligned} \mathfrak {R}^{[+2]}\Psi ^{[+2]} = {\,\underline{L}}\left(w^{-1}{\,\underline{L}}\left(w^{-1}\mathfrak {F}^{[+2]}\right)\right), \\ \mathfrak {R}^{[-2]}\Psi ^{[-2]} = L\left(w^{-1}L \left(w^{-1}\mathfrak {F}^{[-2]}\right)\right), \end{aligned} \end{aligned}$$

where $\psi ^{[\pm 2]},\Psi ^{[\pm 2]}$ are the Chandrasekhar transformations defined by (1.5) and $\mathfrak {R}^{[\pm 2]}$ are the Regge–Wheeler operators of (1.6).

If, moreover, $\widetilde{\alpha }^{[+2]}$ and $\widetilde{\alpha }^{[-2]}$ are regular at infinity and satisfy the conformal Teukolsky boundary conditions (1.2) and $\mathfrak {F}^{[\pm 2]}$ satisfies $\mathfrak {F}^{[\pm 2]}\xrightarrow {r\rightarrow +\infty }0$, $L\mathfrak {F}^{[\pm 2]}\xrightarrow {r\rightarrow +\infty }0$ (hence $\underline{L}\mathfrak {F}^{[\pm 2]}\xrightarrow {r\rightarrow +\infty }0$), then

- $\psi ^{[+2]},\psi ^{[-2]}$ satisfy the following boundary conditions at infinity
  2.11 $$\begin{aligned} \psi ^{[+2]} + (\psi ^{[-2]})^*\xrightarrow {r\rightarrow +\infty } 0 \ \ \ \text {and} \ \ \ r^2\partial _r \psi ^{[+2]} -r^2 \partial _r (\psi ^{[-2]})^*\xrightarrow {r\rightarrow +\infty } 0 \, . \end{aligned}$$
- $\Psi ^{[+2]},\Psi ^{[-2]}$ satisfy the boundary conditions (1.7).

Finally, if $\widetilde{\alpha }^{[+2]}$ and $\Delta ^{-2} \widetilde{\alpha }^{[-2]}$ are regular at the horizon, then $\psi ^{[+2]}$, $\Delta ^{-1} \psi ^{[-2]}$ and $\Psi ^{[\pm 2]}$ are regular at the horizon.

#### Proof

First note that (2.8) is a rewriting of the Teukolsky equations (2.7) using that $\mathfrak {T}^{[+2]} = \mathfrak {R}^{[+2]} + 2\frac{w'}{w}{\,\underline{L}}+ w\left(2-\frac{12M}{r}\right)$ and $\mathfrak {T}^{[-2]} = \mathfrak {R}^{[-2]} - 2\frac{w'}{w}L + w\left(2-\frac{12M}{r}\right)$. With the definitions (1.5), equations (2.7) rewrite as

2.12a $$\begin{aligned} -w^{-1}\mathfrak {F}^{[+2]}&= L\psi ^{[+2]} - \frac{w'}{w}\psi ^{[+2]} + \left(\mathcal {L}^{[+2]}-2+\frac{6M}{r} \right) \widetilde{\alpha }^{[+2]} \end{aligned}$$

2.12b $$\begin{aligned} -w^{-1}\mathfrak {F}^{[-2]}&= {\,\underline{L}}\psi ^{[-2]} + \frac{w'}{w}\psi ^{[-2]} + \left(\mathcal {L}^{[-2]}-2+\frac{6M}{r} \right) \widetilde{\alpha }^{[-2]}. \end{aligned}$$

Taking ${\,\underline{L}}$ and *L* derivatives in the previous equations, we have the following intermediate equations

2.13a $$\begin{aligned} -{\,\underline{L}}\left(w^{-1}\mathfrak {F}^{[+2]}\right)&= L{\,\underline{L}}\psi ^{[+2]} -\frac{w'}{w}{\,\underline{L}}\psi ^{[+2]} + w\left(\mathcal {L}^{[+2]} - \frac{6M}{r} \right)\psi ^{[+2]} +6Mw\widetilde{\alpha }^{[+2]}, \end{aligned}$$

and

2.13b $$\begin{aligned} -L\left(w^{-1}\mathfrak {F}^{[-2]}\right)&= L{\,\underline{L}}\psi ^{[-2]} + \frac{w'}{w}L\psi ^{[-2]} + w\left(\mathcal {L}^{[-2]}-\frac{6M}{r}\right)\psi ^{[-2]} -6Mw\widetilde{\alpha }^{[-2]}, \end{aligned}$$

where we used that $w^{-1}\left(\frac{w'}{w}\right)' = \left(2-\frac{12M}{r}\right)$. This proves (2.9). Equations (2.13) rewrite as

2.14a $$\begin{aligned} -w^{-1}{\,\underline{L}}\left(w^{-1}\mathfrak {F}^{[+2]}\right)&= L\Psi ^{[+2]} + \left(\mathcal {L}^{[+2]} -\frac{6M}{r} \right) \psi ^{[+2]} + 6M \widetilde{\alpha }^{[+2]} \end{aligned}$$

2.14b $$\begin{aligned} -w^{-1}L\left(w^{-1}\mathfrak {F}^{[-2]}\right)&= {\,\underline{L}}\Psi ^{[-2]} + \left(\mathcal {L}^{[-2]}-\frac{6M}{r} \right) \psi ^{[-2]} - 6M\widetilde{\alpha }^{[-2]}. \end{aligned}$$

Taking ${\,\underline{L}}$ and *L* derivatives in the previous equation, we obtain the desired Regge–Wheeler equations (2.10).

Let us define

2.15 $$\begin{aligned} {\mathring{\alpha }}&:= \lim _{r\rightarrow +\infty } \left(\widetilde{\alpha }^{[+2]}+(\widetilde{\alpha }^{[-2]})^*\right),&{\mathring{\psi }}&:= \lim _{r\rightarrow +\infty } \left(\psi ^{[+2]}+(\psi ^{[-2]})^*\right). \end{aligned}$$

Directly from the boundary conditions (1.2), we obtain

2.16 $$\begin{aligned} \psi ^{[+2]}-(\psi ^{[-2]})^*\xrightarrow {r\rightarrow +\infty } 0. \end{aligned}$$

From (2.12), (2.16) and (1.2), and the vanishing of $\mathfrak {F}^{[\pm 2]}$ at infinity, we have

$$\begin{aligned} L\psi ^{[+2]}-{\,\underline{L}}(\psi ^{[-2]})^*\xrightarrow {r\rightarrow +\infty } 0. \end{aligned}$$

Together with (2.16), this proves that (2.11) holds for $\psi ^{[\pm 2]}$. Using (2.16), it also implies

$$\begin{aligned} \begin{aligned} \Psi ^{[+2]}-(\Psi ^{[-2]})^*&= w^{-1}\left(L+{\,\underline{L}}\right)\left(\psi ^{[+2]}-(\psi ^{[-2]})^*\right) \\&- w^{-1}L\psi ^{[+2]}+w^{-1}{\,\underline{L}}(\psi ^{[-2]})^*\xrightarrow {r\rightarrow +\infty } 0, \end{aligned} \end{aligned}$$

which is the first desired boundary condition (1.7a). From (2.12), (2.15) and the vanishing of $\mathfrak {F}^{[\pm 2]}$ at infinity, we have

$$\begin{aligned} L\psi ^{[+2]}+{\,\underline{L}}(\psi ^{[-2]})^*\xrightarrow {r\rightarrow +\infty } -\left(\mathcal {L}-2\right){\mathring{\alpha }}. \end{aligned}$$

Using (2.15), this implies

2.17 $$\begin{aligned} \begin{aligned} \Psi ^{[+2]}+(\Psi ^{[-2]})^*&= w^{-1}\left(L+{\,\underline{L}}\right)\left(\psi ^{[+2]}+(\psi ^{[-2]})^*\right) - w^{-1}L\psi ^{[+2]}-w^{-1}{\,\underline{L}}(\psi ^{[-2]})^*\\&\xrightarrow {r\rightarrow +\infty } 2k^{-2}{\partial }_t{\mathring{\psi }}+ k^{-2}\left(\mathcal {L}-2\right){\mathring{\alpha }}. \end{aligned} \end{aligned}$$

From the equations (2.14), the vanishing of (derivatives of) $\mathfrak {F}^{[\pm 2]}$ at infinity and the boundary conditions (2.15), (2.16), we infer

2.18 $$\begin{aligned} L\Psi ^{[+2]} - {\,\underline{L}}(\Psi ^{[-2]})^*\xrightarrow {r\rightarrow +\infty } -6M{\mathring{\alpha }}. \end{aligned}$$

From the equations (2.14), the vanishing of (derivatives of) $\mathfrak {F}^{[\pm 2]}$ at infinity and (1.2) (2.15), we have

2.19 $$\begin{aligned} L\Psi ^{[+2]} + {\,\underline{L}}(\Psi ^{[-2]})^*\xrightarrow {r\rightarrow +\infty } -\mathcal {L}{\mathring{\psi }}. \end{aligned}$$

Taking a $2{\partial }_t$ derivative of the limit (2.19), we get

$$\begin{aligned} (L+{\,\underline{L}})\left(L\Psi ^{[+2]}+{\,\underline{L}}(\Psi ^{[-2]})^*\right) \xrightarrow {r\rightarrow +\infty } -2\mathcal {L}{\partial }_t{\mathring{\psi }}. \end{aligned}$$

Using the above and (2.17), we get

$$\begin{aligned} (L+{\,\underline{L}})\left(L\Psi ^{[+2]}+(\Psi ^{[-2]})^*\right) + k^2\mathcal {L}\left(\Psi ^{[+2]}+(\Psi ^{[-2]})^*\right)&\xrightarrow {r\rightarrow +\infty } \mathcal {L}\left(\mathcal {L}-2\right){\mathring{\alpha }}, \end{aligned}$$

which, using the Regge–Wheeler equation (2.10) and the vanishing of (derivatives of) $\mathfrak {F}^{[\pm 2]}$ at infinity, rewrites as

$$\begin{aligned} LL\Psi ^{[+2]}+{\,\underline{L}}{\,\underline{L}}(\Psi ^{[-2]})^*&\xrightarrow {r\rightarrow +\infty } \mathcal {L}\left(\mathcal {L}-2\right){\mathring{\alpha }}. \end{aligned}$$

Combining the above and (2.18), the last desired boundary condition (1.7b) follows.

Finally, if $\widetilde{\alpha }^{[+2]}$ and $\Delta ^{-1} \widetilde{\alpha }^{[-2]}$ are regular at the horizon, then the regularity of $\psi ^{[+2]}, \Delta ^{-1}\psi ^{[-2]}$ and $\Psi ^{[\pm 2]}$ follows directly from the definition of the Chandrasekhar transformations (1.5) and the regularity of the vectorfields *L* and $\Delta ^{-1} \underline{L}$. This finishes the proof of the proposition. $\square$

For future reference, we make the following definition in analogy to Definition 2.1:

#### Definition 2.3

We will call $\Psi ^{[\pm 2]}$ solutions to the Regge–Wheeler problem (on $\mathcal {M}$ or $\mathcal {M} \cap \{ t^\star \ge t^\star _0\}$ depending on context) if the $\Psi ^{[\pm 2]}$ are regular at the future event horizon and regular at infinity and satisfy the Regge–Wheeler equations (1.6) together with the boundary conditions (1.7).

### Reverse Chandrasekhar transformations

The following proposition is the reverse analogue to Proposition 2.2.

#### Proposition 2.4

(Reverse Chandrasekhar transformations). Assume that $\Psi ^{[\pm 2]}$ satisfy the inhomogeneous Regge–Wheeler equations

2.20 $$\begin{aligned} \mathfrak {R}^{[\pm 2]}\Psi ^{[\pm 2]}&= \mathfrak {F}^{[\pm 2]}. \end{aligned}$$

Then, the reverse Chandrasekhar transformations $\widetilde{\alpha }^{[\pm 2],r}$ defined by (1.8) satisfy the inhomogeneous Teukolsky equations

2.21 $$\begin{aligned} \mathfrak {T}^{[+2]}\widetilde{\alpha }^{[+2],r}&= \left(\mathcal {L}(\mathcal {L}-2)+12M{\partial }_t\right)\left(L\left(w L\left(w^{-1}\mathfrak {F}^{[+2]}\right)\right) - 2 w' L\left(w^{-1}\mathfrak {F}^{[+2]}\right)\right),\ \end{aligned}$$

2.22 $$\begin{aligned} \mathfrak {T}^{[-2]}\widetilde{\alpha }^{[-2],r}&= \left(\mathcal {L}(\mathcal {L}-2)-12M{\partial }_t\right)\left({\,\underline{L}}\left(w {\,\underline{L}}\left(w^{-1}\mathfrak {F}^{[-2]}\right)\right) + 2 w' {\,\underline{L}}\left(w^{-1}\mathfrak {F}^{[-2]}\right)\right). \end{aligned}$$

If, moreover, $\Psi ^{[\pm 2]}$ are regular at infinity and satisfy the conformal Regge–Wheeler boundary conditions (1.7) and $\mathfrak {F}^{[\pm 2]}$ satisfies $\mathfrak {F}^{[\pm 2]}\xrightarrow {r\rightarrow +\infty }0$, $\underline{L}\mathfrak {F}^{[\pm 2]}\xrightarrow {r\rightarrow +\infty }0$ (hence ${L}\mathfrak {F}^{[\pm 2]}\xrightarrow {r\rightarrow +\infty }0$), then $\widetilde{\alpha }^{[\pm 2],r}$ satisfy the conformal Teukolsky boundary conditions (1.2).

Finally, if $\Psi ^{[\pm 2]}$ are regular at the horizon, then the reverse Chandrasekhar transformed quantities $\widetilde{\alpha }^{[+2],r}$ and $\Delta ^{-2}\widetilde{\alpha }^{[-2],r}$ are regular at the horizon.

#### Proof

The inhomogeneous Regge–Wheeler equations (2.20) rewrite as

2.23a $$\begin{aligned} -w^{-1}\mathfrak {F}^{[+2]}&= {\,\underline{L}}\psi ^{[+2],r} - \frac{w'}{w}\psi ^{[+2],r} + \left(\mathcal {L}-\frac{6M}{r} \right) \Psi ^{[+2]}, \end{aligned}$$

2.23b $$\begin{aligned} -w^{-1}\mathfrak {F}^{[-2]}&= L\psi ^{[-2],r} + \frac{w'}{w}\psi ^{[-2],r} + \left(\mathcal {L}-\frac{6M}{r} \right) \Psi ^{[-2]}. \end{aligned}$$

Taking *L* and ${\,\underline{L}}$ derivatives in (2.23), we get

2.24a $$\begin{aligned} -L\left(w^{-1}\mathfrak {F}^{[+2]}\right)&= L{\,\underline{L}}\psi ^{[+2],r} - \frac{w'}{w}L\psi ^{[+2],r} \nonumber \\&\quad + \left(\mathcal {L}-2+\frac{6M}{r}\right) w\psi ^{[+2],r} + w 6M \Psi ^{[+2]}, \end{aligned}$$

2.24b $$\begin{aligned} -{\,\underline{L}}\left(w^{-1}\mathfrak {F}^{[-2]}\right)&= L{\,\underline{L}}\psi ^{[-2],r} + \frac{w'}{w}{\,\underline{L}}\psi ^{[-2],r}\nonumber \\&\quad + \left(\mathcal {L}-2+\frac{6M}{r}\right) w\psi ^{[-2],r}-w 6M\Psi ^{[-2]}, \end{aligned}$$

where we used again that $w^{-1}\left(\frac{w'}{w}\right)' = 2 -\frac{12M}{r}$. Let us define the intermediate quantities

2.25 $$\begin{aligned} \widetilde{\alpha }^{[+2],r,int}&:= w^2\left(w^{-1}L\right)\psi ^{[+2],r},&\widetilde{\alpha }^{[-2],r,int}&:= w^2\left(w^{-1}{\,\underline{L}}\right)\psi ^{[-2],r}. \end{aligned}$$

With these definitions, equation (2.24) rewrites as

2.26a $$\begin{aligned} -w L\left(w^{-1}\mathfrak {F}^{[+2]}\right)&= {\,\underline{L}}\widetilde{\alpha }^{[+2],r,int} + \left(\mathcal {L}-2+\frac{6M}{r}\right) w^2\psi ^{[+2],r} + w^2 6M \Psi ^{[+2]}, \end{aligned}$$

2.26b $$\begin{aligned} -w {\,\underline{L}}\left(w^{-1}\mathfrak {F}^{[-2]}\right)&= L\widetilde{\alpha }^{[-2],r,int} + \left(\mathcal {L}-2+\frac{6M}{r}\right) w^2\psi ^{[-2],r}-w^2 6M\Psi ^{[-2]}. \end{aligned}$$

Taking *L* and ${\,\underline{L}}$ derivatives in (2.26) and substracting (resp. adding) $2\frac{w'}{w}$ times (2.26), we get

$$\begin{aligned} L\left(w L\left(w^{-1}\mathfrak {F}^{[+2]}\right)\right) - 2 w' L\left(w^{-1}\mathfrak {F}^{[+2]}\right)&= \mathfrak {T}^{[+2]}\widetilde{\alpha }^{[+2],r,int}, \\ {\,\underline{L}}\left(w {\,\underline{L}}\left(w^{-1}\mathfrak {F}^{[-2]}\right)\right) + 2 w' {\,\underline{L}}\left(w^{-1}\mathfrak {F}^{[-2]}\right)&= \mathfrak {T}^{[-2]}\widetilde{\alpha }^{[-2],r,int}. \end{aligned}$$

The right-hand side commutes with the operator $\mathcal {L}(\mathcal {L}-2)\pm 12M{\partial }_t$ and the desired inhomogeneous Teukolsky equations (2.7) for $\widetilde{\alpha }^{[\pm 2]}$ follow.

Let us define

2.27 $$\begin{aligned} \begin{aligned} {\mathring{\beta }}&:= \lim _{r\rightarrow +\infty }\left(\Psi ^{[+2]}-\big (\Psi ^{[-2]}\big )^*\right),&{\gamma _0}&:= \lim _{r\rightarrow +\infty }\left(L\Psi ^{[+2]}-{\,\underline{L}}\big (\Psi ^{[-2]}\big )^*\right),\\ {\mathring{\delta }}&:= \lim _{r\rightarrow +\infty }\left(\Psi ^{[+2]}+\big (\Psi ^{[-2]}\big )^*\right),&{\mathring{\epsilon }}&:= \lim _{r\rightarrow +\infty }\left(L\Psi ^{[+2]}+{\,\underline{L}}\big (\Psi ^{[-2]}\big )^*\right). \end{aligned} \end{aligned}$$

With these definitions, the Regge–Wheeler boundary conditions (1.7) rewrite as

2.28 $$\begin{aligned} {\mathring{\beta }}&= 0,&2{\partial }_t{\mathring{\epsilon }}+ k^2\mathcal {L}{\mathring{\delta }}+\frac{\mathcal {L}(\mathcal {L}-2)}{6M}{\gamma _0}&= 0. \end{aligned}$$

Directly from the definitions (1.8) and (2.27), one has

2.29 $$\begin{aligned} \begin{aligned} \psi ^{[+2],r}-\big (\psi ^{[-2],r}\big )^*&\xrightarrow {r\rightarrow +\infty } k^{-2} {\gamma _0}, \\ \psi ^{[+2],r}+\big (\psi ^{[-2],r}\big )^*&\xrightarrow {r\rightarrow +\infty } k^{-2} {\mathring{\epsilon }}. \end{aligned} \end{aligned}$$

Using the limits (2.29), and taking the limit in equation (2.23) using the definitions (2.27) and the vanishing of $\mathfrak {F}^{[\pm 2]}$ at infinity, we infer

$$\begin{aligned} \begin{aligned} L\psi ^{[+2],r} - {\,\underline{L}}\big (\psi ^{[+2],r}\big )^*&= (L+{\,\underline{L}})\left(\psi ^{[+2],r}-\big (\psi ^{[-2],r}\big )^*\right) - {\,\underline{L}}\psi ^{[+2],r} + L\big (\psi ^{[-2],r}\big )^*\\&\xrightarrow {r\rightarrow +\infty } 2k^{-2}{\partial }_t{\gamma _0}+\mathcal {L}{\mathring{\beta }}, \end{aligned} \end{aligned}$$

which, for the intermediate Teukolsky quantity defined in (2.25), rewrites as

2.30 $$\begin{aligned} \widetilde{\alpha }^{[+2],r,int} - \big (\widetilde{\alpha }^{[-2],r,int}\big )^*&\xrightarrow {r\rightarrow +\infty } 2{\partial }_t{\gamma _0}+k^2\mathcal {L}{\mathring{\beta }}. \end{aligned}$$

Along the dual lines, we get

2.31 $$\begin{aligned} \widetilde{\alpha }^{[+2],r,int}+\big (\widetilde{\alpha }^{[-2],r,int}\big )^*&\xrightarrow {r\rightarrow +\infty } 2{\partial }_t{\mathring{\epsilon }}+k^2\mathcal {L}{\mathring{\delta }}. \end{aligned}$$

Taking the limits in linear combinations of equation (2.26), using the definitions (2.27) and the vanishing of $\mathfrak {F}^{[\pm 2]}$ and its derivatives at infinity, we get

2.32 $$\begin{aligned} \begin{aligned} {\,\underline{L}}\widetilde{\alpha }^{[+2],r,int}-L\big (\widetilde{\alpha }^{[-2],r,int}\big )^*&\xrightarrow {r\rightarrow +\infty } -k^2(\mathcal {L}-2){\gamma _0}- 6Mk^4 {\mathring{\delta }},\\ {\,\underline{L}}\widetilde{\alpha }^{[+2],r,int}+L\big (\widetilde{\alpha }^{[-2],r,int}\big )^*&\xrightarrow {r\rightarrow +\infty } -k^2(\mathcal {L}-2){\mathring{\epsilon }}- 6Mk^4 {\mathring{\beta }}. \end{aligned} \end{aligned}$$

Using the definitions (1.8) of $\widetilde{\alpha }^{[\pm 2],r}$, the limits (2.30), (2.31) and the rewriting of the Regge–Wheeler boundary conditions (2.28), we have

$$\begin{aligned} \widetilde{\alpha }^{[+2],r} - \big (\widetilde{\alpha }^{[-2],r}\big )^*&= \mathcal {L}(\mathcal {L}-2)\left(\widetilde{\alpha }^{[+2],r,int} - \big (\widetilde{\alpha }^{[-2],r,int}\big )^*\right) \\&\quad + 12M{\partial }_t\left(\widetilde{\alpha }^{[+2],r,int} + \big (\widetilde{\alpha }^{[-2],r,int}\big )^*\right)\\&\xrightarrow {r\rightarrow +\infty } \mathcal {L}(\mathcal {L}-2)\left(2{\partial }_t{\gamma _0}+k^2\mathcal {L}{\mathring{\beta }}\right) + 12M{\partial }_t\left(2{\partial }_t{\mathring{\epsilon }}+k^2\mathcal {L}{\mathring{\delta }}\right) \\&= k^2\mathcal {L}^2(\mathcal {L}-2){\mathring{\beta }}+ 12M{\partial }_t\left(2{\partial }_t{\mathring{\epsilon }}+k^2\mathcal {L}{\mathring{\delta }}+\frac{\mathcal {L}(\mathcal {L}-2)}{6M}{\gamma _0}\right) \\&= 0. \end{aligned}$$

Similarly, using (2.32) and (2.28), we have

$$\begin{aligned}&{\,\underline{L}}\widetilde{\alpha }^{[+2],r}-L\big (\widetilde{\alpha }^{[-2],r}\big )^*\\&\quad = \mathcal {L}(\mathcal {L}-2)\left({\,\underline{L}}\widetilde{\alpha }^{[+2],r,int}-L\big (\widetilde{\alpha }^{[-2],r,int}\big )^*\right) \\ &\qquad + 12M{\partial }_t\left({\,\underline{L}}\widetilde{\alpha }^{[+2],r,int}+L\big (\widetilde{\alpha }^{[-2],r,int}\big )^*\right) \\&\qquad \xrightarrow {r\rightarrow +\infty } \mathcal {L}(\mathcal {L}-2)\left(-k^2(\mathcal {L}-2){\gamma _0}- 6Mk^4 {\mathring{\delta }}\right) + 12M{\partial }_t\left(-k^2(\mathcal {L}-2){\mathring{\epsilon }}- 6Mk^4 {\mathring{\beta }}\right) \\&\quad = -72M^2k^4{\partial }_t{\mathring{\beta }}- 6Mk^2(\mathcal {L}-2)\left(2{\partial }_t{\mathring{\epsilon }}+k^2\mathcal {L}{\mathring{\delta }}+\frac{\mathcal {L}(\mathcal {L}-2)}{6M}{\gamma _0}\right) \\&\quad = 0. \end{aligned}$$

From these two limits, we deduce that $\widetilde{\alpha }^{[\pm 2],r}$ satisfy the Teukolsky conformal Anti-de Sitter boundary conditions (1.2).

Finally, the regularity at the horizon of $\widetilde{\alpha }^{[\pm 2],r}$ follows directly from the definition of the reverse Chandrasekhar transformations (1.8), the regularity of $\Psi ^{[\pm 2]}$ and that of the vectorfields *L* and $\Delta ^{-1}\underline{L}$. This finishes the proof of the proposition. $\square$

#### Remark 2.5

The fact that the reverse Chandrasekhar transformed quantities satisfy the Teukolsky equations was obtained in [Cha83, §30]—although this was proved in the special $k=0$ case –, see relation (318) with coefficients given by (28), (327), (328).

#### Remark 2.6

In Proposition 2.2 (see (2.10)), we showed that for all $\widetilde{\alpha }^{[\pm 2]}$,

2.33 $$\begin{aligned} \begin{aligned} \mathfrak {R}^{[+2]}\left((w^{-1}{\,\underline{L}})^2\widetilde{\alpha }^{[+2]}\right)&= {\,\underline{L}}\left(w^{-1}{\,\underline{L}}\left(w^{-1}\mathfrak {T}^{[+2]}\widetilde{\alpha }^{[+2]}\right)\right), \\ \mathfrak {R}^{[-2]}\left((w^{-1}L)^2\widetilde{\alpha }^{[-2]}\right)&= L\left(w^{-1}L\left(w^{-1}\mathfrak {T}^{[-2]}\widetilde{\alpha }^{[-2]}\right)\right). \end{aligned} \end{aligned}$$

Note that on the RHS of (2.33), the Teukolsky operators $\mathfrak {T}^{[\pm 2]}$ are composed with the formal adjoints (for the $L^2({\textrm{d}}t{\textrm{d}}r^\star {\textrm{d}}{\vartheta }\sin {\vartheta }{\textrm{d}}\varphi )$ inner product) of the Chandrasekhar transformations $(w^{-1}{\,\underline{L}})^2,(w^{-1}L)^2$ respectively. Moreover, the Teukolsky operators $\mathfrak {T}^{[\pm 2]}$ are the formal adjoints of the $\mathfrak {T}^{[\mp 2]}$ operators and the Regge–Wheeler operators $\mathfrak {R}^{[\pm 2]}$ are self-adjoint. Hence, following Wald’s duality argument [Wal78], it is immediate that the transformations

2.34 $$\begin{aligned} \begin{aligned} \widetilde{\alpha }^{[+2],r,int}&:= w^2\left(w^{-1}L\right)^2\Psi ^{[+2]},&\widetilde{\alpha }^{[-2],r,int}&:= w^2\left(w^{-1}{\,\underline{L}}\right)^2\Psi ^{[-2]}, \end{aligned} \end{aligned}$$

satisfy the Teukolsky equations (2.5) if $\Psi ^{[\pm 2]}$ satisfy the Regge–Wheeler equations (1.6). However, as it is clear from the proof of Proposition 2.4, $\widetilde{\alpha }^{[\pm 2],r,int}$ do not satisfy the desired boundary conditions (1.2) if $\Psi ^{[\pm 2]}$ satisfy the Regge–Wheeler boundary conditions (1.7). One retrieves the Teukolsky boundary conditions for (1.8) only after composition of (2.34) with the operators $(\mathcal {L}(\mathcal {L}-2)\pm 12M{\partial }_t)$ (see Proposition 2.4). We discuss these “Teukolsky–Starobinsky” operators in Sect. 2.7. Interestingly, they appear naturally when composing the Chandrasekhar transformations, as in the identities (2.41) and (2.42), and as in the Teukolsky–Starobinsky identities (1.3). See also Lemma 2.20.

### The decoupled Regge–Wheeler problem

We have the following proposition.

#### Proposition 2.7

(Decoupled Regge–Wheeler problem). Let $\Psi ^{[+2]},(\Psi ^{[-2]})^*,\Psi ^D,\Psi ^R$ be four spin-$+2$-weighted functions satisfying the relations^5^

2.35 $$\begin{aligned} \begin{aligned} \Psi ^D&= \Psi ^{[+2]} - \big (\Psi ^{[-2]}\big )^*, \\ \Psi ^R&= \left(\Psi ^{[+2]} + \big (\Psi ^{[-2]}\big )^*\right) +\frac{12M}{\mathcal {L}(\mathcal {L}-2)}{\partial }_t\left(\Psi ^{[+2]} - \big (\Psi ^{[-2]}\big )^*\right). \end{aligned} \end{aligned}$$

Let $\mathfrak {F}^D,\mathfrak {F}^R$ be two spin-$+2$-weighted functions, vanishing at infinity. The following two items are equivalent.

- $\Psi ^D,\Psi ^R$ are solutions to the (inhomogeneous) Regge–Wheeler equations
  2.36a $$\begin{aligned} \mathfrak {R}\Psi ^D&= \mathfrak {F}^D,&\mathfrak {R}\Psi ^R&= \mathfrak {F}^R, \end{aligned}$$
  where $\mathfrak {R}=\mathfrak {R}^{[+2]}$, and $\Psi ^D,\Psi ^R$ are regular at infinity and satisfy the boundary conditions
  2.36ba $$\begin{aligned} \Psi ^D&\xrightarrow {r\rightarrow +\infty } 0, \end{aligned}$$
  2.36bb $$\begin{aligned} 2{\partial }_{t}^2\Psi ^R + \frac{\mathcal {L}(\mathcal {L}-2)}{6M}{\partial }_{r^\star }\Psi ^R + k^2\mathcal {L}\Psi ^R&\xrightarrow {r\rightarrow +\infty } 0, \end{aligned}$$
  and $\Psi ^{D},\Psi ^R$ are regular at the horizon.
- $\Psi ^{[+2]},\Psi ^{[-2]}$ are solutions to the inhomogeneous Regge–Wheeler equations
  2.37 $$\begin{aligned} \begin{aligned} \mathfrak {R}^{[+2]}\Psi ^{[+2]}&= +\frac{1}{2}\mathfrak {F}^D +\frac{1}{2}\left(\mathfrak {F}^R-\frac{12M}{\mathcal {L}(\mathcal {L}-2)}{\partial }_t\mathfrak {F}^D\right),\\ \mathfrak {R}^{[-2]}\Psi ^{[-2]}&= -\frac{1}{2}\big (\mathfrak {F}^D\big )^*+\frac{1}{2}\left(\mathfrak {F}^R-\frac{12M}{\mathcal {L}(\mathcal {L}-2)}{\partial }_t\mathfrak {F}^D\right)^*, \end{aligned} \end{aligned}$$
  and $\Psi ^{[\pm 2]}$ are regular at infinity and satisfy the boundary conditions (1.7) and are regular at the horizon.

In the following, we call (2.36) the (inhomogeneous) *decoupled Regge–Wheeler problem*.

#### Remark 2.8

For further reference, we record that relation (2.35) rewrites as

2.38 $$\begin{aligned} \begin{aligned} \Psi ^{[+2]}&= +\frac{1}{2}\Psi ^D+\frac{1}{2}\left(\Psi ^R-\frac{12M}{\mathcal {L}(\mathcal {L}-2)}{\partial }_t\Psi ^D\right),\\ \big (\Psi ^{[-2]}\big )^*&= -\frac{1}{2}\Psi ^D+\frac{1}{2}\left(\Psi ^R-\frac{12M}{\mathcal {L}(\mathcal {L}-2)}{\partial }_t\Psi ^D\right). \end{aligned} \end{aligned}$$

#### Proof of Proposition 2.7

The equivalence between equations (2.36a) and (2.37) as well as the equivalence of the regularity conditions at the horizon is a direct consequence from the fact that the transformations (2.35) (and its inverse (2.38)) commute with the operators $L,{\,\underline{L}}$ and $\mathcal {L}$ and with all radial functions. Concerning the equivalence of the boundary conditions (2.36b) and (1.7), let us first define the intermediate quantity $\Psi ^N := \Psi ^{[+2]}+\big (\Psi ^{[-2]}\big )^*$. With this definition, we write

2.39 $$\begin{aligned} \begin{aligned}&LL\Psi ^{[+2]} + {\,\underline{L}}{\,\underline{L}}\big (\Psi ^{[-2]}\big )^*+ \frac{\mathcal {L}(\mathcal {L}-2)}{6M}\left(L\Psi ^{[+2]}-{\,\underline{L}}\big (\Psi ^{[-2]}\big )^*\right) \\&\quad = \frac{1}{2}\left(LL+{\,\underline{L}}{\,\underline{L}}\right)\Psi ^N + \frac{1}{2}\left(LL-{\,\underline{L}}{\,\underline{L}}\right)\Psi ^D + \frac{\mathcal {L}(\mathcal {L}-2)}{12M}\left((L+{\,\underline{L}})\Psi ^D + (L-{\,\underline{L}})\Psi ^N\right) \\&\quad = \frac{1}{2}\left(LL+{\,\underline{L}}{\,\underline{L}}\right)\Psi ^N + \frac{1}{12M}(L-{\,\underline{L}})\left(12M{\partial }_t\Psi ^D+ \mathcal {L}(\mathcal {L}-2)\Psi ^N\right) + \frac{\mathcal {L}(\mathcal {L}-2)}{6M}{\partial }_t\Psi ^D \\&\quad = \frac{1}{2}\left(LL+{\,\underline{L}}{\,\underline{L}}\right)\Psi ^R + \frac{\mathcal {L}(\mathcal {L}-2)}{12M}(L-{\,\underline{L}})\Psi ^R \\&\qquad - \frac{1}{2}\left(LL+{\,\underline{L}}{\,\underline{L}}\right)\frac{12M}{\mathcal {L}(\mathcal {L}-2)}{\partial }_t\Psi ^D + \frac{\mathcal {L}(\mathcal {L}-2)}{6M}{\partial }_t\Psi ^D. \end{aligned} \end{aligned}$$

If $\Psi ^D \xrightarrow {r\rightarrow +\infty } 0$, and if $\Psi ^D$ satisfies the Regge–Wheeler equation (2.36a) with vanishing $\mathfrak {F}^D$ at infinity, we have

$$\begin{aligned} \mathcal {L}(\mathcal {L}-2){\partial }_t\Psi ^D \xrightarrow {r\rightarrow +\infty } 0, \end{aligned}$$

and

$$\begin{aligned} \frac{1}{2}(LL+{\,\underline{L}}{\,\underline{L}})\frac{12M}{\mathcal {L}(\mathcal {L}-2)}{\partial }_t\Psi ^D&= \frac{12M}{\mathcal {L}(\mathcal {L}-2)}{\partial }_t\left({\partial }_t^2+{\partial }_{r^\star }^2\right)\Psi ^D \\&= \frac{12M}{\mathcal {L}(\mathcal {L}-2)}{\partial }_t\left(2{\partial }_t^2 + \frac{\Delta }{r^4}\left(\mathcal {L}-\frac{6M}{r}\right) - \mathfrak {F}^D\right)\Psi ^D \\&\xrightarrow {r\rightarrow +\infty } 0. \end{aligned}$$

Plugging the above two limits in (2.39), we get that if $\Psi ^D \xrightarrow {r\rightarrow +\infty } 0$, and if $\Psi ^D$ satisfies the Regge–Wheeler equation (2.36a) with vanishing $\mathfrak {F}^D$ at infinity, the boundary condition (1.7b) is equivalent to

2.40 $$\begin{aligned} \begin{aligned} \frac{1}{2}\left(LL+{\,\underline{L}}{\,\underline{L}}\right)\Psi ^R + \frac{\mathcal {L}(\mathcal {L}-2)}{12M}(L-{\,\underline{L}})\Psi ^R&\xrightarrow {r\rightarrow +\infty } 0. \end{aligned} \end{aligned}$$

Now, (2.40) is equivalent to

$$\begin{aligned}&\left({\partial }_t^2+{\partial }_{r^\star }^2\right)\Psi ^R + \frac{\mathcal {L}(\mathcal {L}-2)}{6M}{\partial }_{r^\star }\Psi ^R \\&\quad = \frac{1}{2}\left(LL+{\,\underline{L}}{\,\underline{L}}\right)\Psi ^R + \frac{\mathcal {L}(\mathcal {L}-2)}{12M}(L-{\,\underline{L}})\Psi ^R \xrightarrow {r\rightarrow +\infty } 0, \end{aligned}$$

which, if $\Psi ^R$ satisfies the Regge–Wheeler equation (2.36a) with vanishing $\mathfrak {F}^R$ at infinity is equivalent to

$$\begin{aligned} 2{\partial }_{t}^2\Psi ^R + \frac{\mathcal {L}(\mathcal {L}-2)}{6M}{\partial }_{r^\star }\Psi ^R + k^2\mathcal {L}\Psi ^R&\xrightarrow {r\rightarrow +\infty } 0. \end{aligned}$$

This finishes the proof of the equivalence of the boundary conditions and of the proposition. $\square$

### Additional relations for the Chandrasekhar transformations

We have the following two lemmas. Their proofs are direct computations which are left to the reader.

#### Lemma 2.9

If $\widetilde{\alpha }^{[+2]}$ be a solution to the Teukolsky equation (2.5a), then

2.41a $$\begin{aligned} \left(\mathcal {L}^{[+2]}(\mathcal {L}^{[+2]}-2) - 12M{\partial }_t\right)\widetilde{\alpha }^{[+2]}&= w^2(w^{-1}L)(w^{-1}L)(w^{-1}{\,\underline{L}})(w^{-1}{\,\underline{L}}) \widetilde{\alpha }^{[+2]}, \end{aligned}$$

If $\widetilde{\alpha }^{[-2]}$ be a solution to the Teukolsky equation (2.5b), then

2.41b $$\begin{aligned} \left(\mathcal {L}^{[-2]}(\mathcal {L}^{[-2]}-2) + 12M{\partial }_t\right)\widetilde{\alpha }^{[-2]}&= w^2(w^{-1}{\,\underline{L}})(w^{-1}{\,\underline{L}})(w^{-1}L)(w^{-1}L) \widetilde{\alpha }^{[-2]}. \end{aligned}$$

#### Lemma 2.10

Let $\Psi$ be a solution to the Regge–Wheeler equation (1.6). Then,

2.42 $$\begin{aligned} \begin{aligned} (w^{-1}{\,\underline{L}})(w^{-1}{\,\underline{L}})w^2(w^{-1}L)(w^{-1}L)\Psi&= \left(\mathcal {L}(\mathcal {L}-2)-12M{\partial }_t\right)\Psi ,\\ \\ (w^{-1}L)(w^{-1}L)w^2(w^{-1}{\,\underline{L}})(w^{-1}{\,\underline{L}})\Psi&= \left(\mathcal {L}(\mathcal {L}-2)+12M{\partial }_t\right)\Psi . \end{aligned} \end{aligned}$$

#### Definition 2.11

In the following, we will call

$$\begin{aligned} \left(\mathcal {L}(\mathcal {L}-2) - 12M{\partial }_t\right) &  \text {and} &  \left(\mathcal {L}(\mathcal {L}-2) + 12M{\partial }_t\right), \end{aligned}$$

respectively the growing and decaying Teukolsky–Starobinsky operators. Elements of their respective kernels will be called *growing* and *decaying* *Teukolsky–Starobinsky modes*.^6^

#### Remark 2.12

From the formulas (2.41) and (2.42) and the definitions of the (reverse) Chandrasekhar transformations (1.5) and (1.8), one can directly deduce that Teukolsky (resp. Regge–Wheeler) quantities with vanishing Chandrasekhar (resp. reverse Chandrasekhar) transformations are Teukolsky–Starobinsky modes as defined in Definition 2.11.

### Teukolsky–Starobinsky identities

We recall the Teukolsky Starobinsky identities introduced in Definition 1.2. To first verify the claim made in Remark 1.3, we recall from Section 4.1.1 of [GH24a] the relationswhere $\mathfrak {m}:= \frac{1}{r}\left({\partial }_{\vartheta }+ \frac{i}{\sin {\vartheta }} {\partial }_\varphi \right)$, and use the following easily verified formulaewhere *f*, *g* are scalars, *X* is a 1-tensor and $\alpha$ a symmetric traceless 2-tensor tangent to the spheres. See for instance Section 2.3.3 of [GH24a] for the definition of the angular operators appearing on the left.

As we will construct $\widetilde{\alpha }^{[\pm 2]}$ from Regge–Wheeler quantities using the (reverse) Chandrasekhar transformations, we want to understand which constraints the Teukolsky–Starobinsky identities (1.3) impose on $\Psi ^{[\pm 2]}$. Before that, it will be useful to rewrite (1.3) using the operators $\mathcal {L}^{[\pm 2]}$ and set an appropriate normalisation for the Hilbert basis of spherical modes.

#### Angular decompositions

##### Lemma 2.13

(Angular Teukolsky–Starobinsky identities). The following operator identities hold true

2.43 $$\begin{aligned} \begin{aligned} \mathcal {L}_{-1}\mathcal {L}^{\dagger }_2\mathcal {L}_{-1}\mathcal {L}_{0}\mathcal {L}_{1}\mathcal {L}_{2}&= \mathcal {L}_{-1}\mathcal {L}_{0}\mathcal {L}_{1}\mathcal {L}_{2}\mathcal {L}_{-1}^{\dagger }\mathcal {L}_2 \end{aligned} \end{aligned}$$

and

2.44 $$\begin{aligned} \begin{aligned} \mathcal {L}_{-1}^{\dagger }\mathcal {L}_{0}^{\dagger }\mathcal {L}_{1}^{\dagger }\mathcal {L}_{2}^{\dagger }\mathcal {L}_{-1}\mathcal {L}_{0}\mathcal {L}_{1}\mathcal {L}_{2}&= \big (\mathcal {L}_{-1}^{\dagger }\mathcal {L}_2\big )^2\big (\mathcal {L}^{\dagger }_{-1}\mathcal {L}_2-2\big )^2. \end{aligned} \end{aligned}$$

Moreover, we have

2.45 $$\begin{aligned} \mathcal {L}^{[+2]}&= -\mathcal {L}_{-1}\mathcal {L}_2^{\dagger }+2,&\mathcal {L}^{[-2]}&= -\mathcal {L}^{\dagger }_{-1}\mathcal {L}_2 +2. \end{aligned}$$

##### Proof

Identities (2.43) and (2.44) both follow from applying repeatedly the following commutation identities

$$\begin{aligned} \mathcal {L}_{2}^{\dagger }\mathcal {L}_{-1}&= \mathcal {L}_0\mathcal {L}_{1}^{\dagger }+ 2,&\mathcal {L}^{\dagger }_1\mathcal {L}_0&= \mathcal {L}_1\mathcal {L}^{\dagger }_{0},&\mathcal {L}^{\dagger }_{0}\mathcal {L}_1&= \mathcal {L}_2\mathcal {L}_{-1}^{\dagger }-2, \end{aligned}$$

which are easily verified. Identities (2.45) follow from direct computations using the definition (2.6) of $\mathcal {L}^{[\pm 2]}$. $\square$

##### Proposition 2.14

There exists a Hilbert basis $\left(S_{m\ell }({\vartheta })e^{+im\varphi }\right)_{\ell \ge 2, |m|\le \ell }$ of spin- $+2$-weighted complex-valued $L^2$ functions, so that, for all integers $\ell \ge 2$ and $|m|\le \ell$,

1. $S_{m\ell }({\vartheta })e^{+im\varphi }$ are smooth spin-$+2$-weighted complex-valued functions of $L^2$ norm 1 and $S_{m\ell }^*=S_{m\ell }$,
2. $S_{m\ell }({\vartheta })e^{+im\varphi }$ are eigenvectors of $\mathcal {L}^{[+2]}$:
  2.46 $$\begin{aligned} \mathcal {L}^{[+2]}\left(S_{m\ell }({\vartheta })e^{+im\varphi }\right)&= \ell (\ell +1)\left(S_{m\ell }({\vartheta })e^{+im\varphi }\right), \end{aligned}$$
3. $S_{m\ell }({\vartheta })e^{-im\varphi }$ are eigenvectors of $\mathcal {L}^{[-2]}$:
  2.47 $$\begin{aligned} \mathcal {L}^{[-2]}\left(S_{m\ell }({\vartheta })e^{-im\varphi }\right)&= \ell (\ell +1)\left(S_{m\ell }({\vartheta })e^{-im\varphi }\right), \end{aligned}$$
4. The $S_{m\ell }$ and $S_{(-m)\ell }$ functions are related by the following Teukolsky–Starobinsky transformations
  2.48 $$\begin{aligned} \begin{aligned} \mathcal {L}_{-1}^{\dagger }\mathcal {L}_{0}^{\dagger }\mathcal {L}_{1}^{\dagger }\mathcal {L}_{2}^{\dagger }\left(S_{m\ell }({\vartheta })e^{+im\varphi }\right)&= (\ell -1)\ell (\ell +1)(\ell +2)\left(S_{-m\ell }({\vartheta })e^{+im\varphi }\right) \\ \mathcal {L}_{-1}\mathcal {L}_{0}\mathcal {L}_{1}\mathcal {L}_{2}\left(S_{m\ell }({\vartheta })e^{-im\varphi }\right)&= (\ell -1)\ell (\ell +1)(\ell +2)\left(S_{-m\ell }({\vartheta })e^{-im\varphi }\right). \end{aligned} \end{aligned}$$

##### Proof

The existence of a Hilbert basis satisfying items 1, 2 and 3 is a standard result of Sturm-Liouville theory for the operators $\mathcal {L}^{[\pm 2]}$. From the commutation relation (2.43), relation (2.45), and item 3 of the lemma, we have that

$$\begin{aligned} \mathcal {L}^{[+2]}\bigg (\mathcal {L}_{-1}\mathcal {L}_{0}\mathcal {L}_{1}\mathcal {L}_{2} \left(S_{m\ell }({\vartheta })e^{-im\varphi }\right)\bigg )&= \ell (\ell +1)\bigg (\mathcal {L}_{-1}\mathcal {L}_{0}\mathcal {L}_{1}\mathcal {L}_{2} \left(S_{m\ell }({\vartheta })e^{-im\varphi }\right)\bigg ). \end{aligned}$$

Moreover the operators $\mathcal {L}_n$ map regular spin-$(-n)$-weighted complex functions to regular spin-$(-n+1)$-weighted complex functions, hence $\mathcal {L}_{-1}\mathcal {L}_{0}\mathcal {L}_{1}\mathcal {L}_{2} \left(S_{m\ell }({\vartheta })e^{-im\varphi }\right)$ is a regular spin-$+2$-weighted complex function, and using items 1 and 2 of the lemma, one deduces that there exists $\aleph _{m\ell }\in \mathbb {C}$ such that

2.49 $$\begin{aligned} \mathcal {L}_{-1}\mathcal {L}_{0}\mathcal {L}_{1}\mathcal {L}_{2} \left(S_{m\ell }({\vartheta })e^{-im\varphi }\right)&= \aleph _{m\ell }\left(S_{-m\ell }({\vartheta })e^{-im\varphi }\right), \end{aligned}$$

for all $\ell \ge 2$ and all $|m|\le \ell$. From (2.44), (2.45), (2.47) and (2.49), noting that $(\ell -1)\ell (\ell +1)(\ell +2) = \ell (\ell +1)\big (\ell (\ell +1)-2\big )$, one has that

2.50 $$\begin{aligned} \aleph _{m\ell }\aleph _{-m\ell }^*&= \big ((\ell -1)\ell (\ell +1)(\ell +2)\big )^2. \end{aligned}$$

Moreover, from (2.44), (2.45), (2.47) and (2.49) and integration by parts, one has

2.51 $$\begin{aligned} \begin{aligned} |\aleph _{m\ell }|^2&= \int _{S^2}\left|\mathcal {L}_{-1}\mathcal {L}_{0}\mathcal {L}_{1}\mathcal {L}_{2} \left(S_{m\ell }({\vartheta })e^{-im\varphi }\right)\right|^2\,\sin {\vartheta }{\textrm{d}}{\vartheta }{\textrm{d}}\varphi \\&= \int _{S^2}S_{m\ell }({\vartheta })e^{+im\varphi }\mathcal {L}_{-1}^{\dagger }\mathcal {L}_{0}^{\dagger }\mathcal {L}_{1}^{\dagger }\mathcal {L}_{2}^{\dagger }\mathcal {L}_{-1}\mathcal {L}_{0}\mathcal {L}_{1}\mathcal {L}_{2}\left(S_{m\ell }({\vartheta })e^{-im\varphi }\right)\,\sin {\vartheta }{\textrm{d}}{\vartheta }{\textrm{d}}\varphi \\&= \big ((\ell -1)\ell (\ell +1)(\ell +2)\big )^2. \end{aligned} \end{aligned}$$

It is immediate from (2.49), the definition (1.4) of the $\mathcal {L}_n$ and the fact that the $S_{m\ell }$ are real-valued (item 1), that $\aleph _{m\ell }\in \mathbb {R}$ for all $\ell \ge 2$ and all $|m|\le \ell$. Hence, from (2.50) and (2.51), we deduce that

2.52 $$\begin{aligned} \aleph _{m\ell } = \aleph _{-m\ell } = \pm (\ell -1)\ell (\ell +1)(\ell +2), \end{aligned}$$

for all $\ell \ge 2$ and all $|m|\le \ell$. Up to changing the sign of the $S_{m\ell }$ with $m<0$, one can thus assume that $\aleph _{m\ell } = \aleph _{-m\ell } = (\ell -1)\ell (\ell +1)(\ell +2)$ for all $m\ne 0$. For $m=0$, multiplying (2.49) by $S_{0\ell }$ and integrating on the sphere, we have

$$\begin{aligned} \aleph _{0\ell }&= \int _{S^2}S_{0\ell }({\vartheta })\mathcal {L}_{-1}\mathcal {L}_{0}\mathcal {L}_{1}\mathcal {L}_{2}S_{0\ell }({\vartheta })\,\sin {\vartheta }{\textrm{d}}{\vartheta }{\textrm{d}}\varphi \\&= \int _{S^2}\left|\mathcal {L}_{1}\mathcal {L}_{2}S_{0\ell }({\vartheta })\right|^2\,\sin {\vartheta }{\textrm{d}}{\vartheta }{\textrm{d}}\varphi \ge 0. \end{aligned}$$

Thus, from (2.52), we infer that $\aleph _{0\ell } = (\ell -1)\ell (\ell +1)(\ell +2)$ and this finishes the proof of item 4. $\square$

##### Definition 2.15

For a spin-$+2$-weighted complex valued function $\phi$, we denote by $\phi _{m\ell }$ the coefficients of $\phi$ on the basis $\left(S_{m\ell }({\vartheta })e^{+im\varphi }\right)_{\ell \ge 2, |m|\le \ell }$. For a spin-$-2$-weighted complex valued function $\phi$, we denote by $\phi _{m\ell }$ the coefficients of $\phi$ on the basis $\left(S_{m\ell }({\vartheta })\right. \left. e^{-im\varphi }\right)_{\ell \ge 2, |m|\le \ell }$.

##### Definition 2.16

We define the conjugacy operators $\mathcal {C},\mathcal {C}^*$ from spin-$+2$ to spin-$-2$ weighted (resp. spin-$-2$ to spin-$+2$) functions by their action on the basis elements as follows:

$$\begin{aligned} \mathcal {C}&: \left(S_{m\ell }({\vartheta })e^{+im\varphi }\right) \mapsto \left(S_{-m\ell }({\vartheta })e^{+im\varphi }\right), \\ \mathcal {C}^*&: \left(S_{m\ell }({\vartheta })e^{-im\varphi }\right) \mapsto \left(S_{-m\ell }({\vartheta })e^{-im\varphi }\right). \end{aligned}$$

Note that we have

$$\begin{aligned} \mathcal {C}\mathcal {C}^*&= \textrm{Id},&\mathcal {C}^*\mathcal {C}&= \textrm{Id}. \end{aligned}$$

##### Corollary 2.17

We can write

2.53 $$\begin{aligned} \mathcal {L}_{-1}^{\dagger }\mathcal {L}_{0}^{\dagger }\mathcal {L}_{1}^{\dagger }\mathcal {L}_{2}^{\dagger }&= \mathcal {L}^{[-2]}(\mathcal {L}^{[-2]}-2)\mathcal {C},&\mathcal {L}_{-1}\mathcal {L}_{0}\mathcal {L}_{1}\mathcal {L}_{2}&= \mathcal {L}^{[+2]}(\mathcal {L}^{[+2]}-2)\mathcal {C}^*. \end{aligned}$$

##### Proof

Use the relations (2.48), (2.46), (2.47) and that $\ell (\ell +1)(\ell (\ell +1)-2) = (\ell -1)\ell (\ell +1)(\ell +2)$. $\square$

##### Definition 2.18

(Symmetrised and anti-symmetrised quantities) For all spin-$\pm 2$-weighted complex valued functions $\phi ^{[\pm 2]}$, define^7^

2.54 $$\begin{aligned} \begin{aligned} \phi ^{[+2]}_s&:= \frac{1}{2}\phi ^{[+2]} + \frac{1}{2}(\mathcal {C}\phi ^{[+2]})^*,&\phi _a^{[+2]}&:= \frac{1}{2}\phi ^{[+2]} - \frac{1}{2}(\mathcal {C}\phi ^{[+2]})^*,\\ \phi ^{[-2]}_s&:= \frac{1}{2}\phi ^{[-2]} + \frac{1}{2}\mathcal {C}(\phi ^{[-2]})^*,&\phi _a^{[-2]}&:= \frac{1}{2}\phi ^{[-2]} - \frac{1}{2}\mathcal {C}(\phi ^{[-2]})^*. \end{aligned} \end{aligned}$$

##### Remark 2.19

The symmetrised and anti-symmetrised decompositions (2.54) have a direct interpretation in terms of Hodge decomposition of symmetric traceless 2-tensors. Namely, using the formulae from Sect. 2.8, from Lemma 2.13 and (2.53), for all *S*-tangent symmetric traceless 2-tensors $\Phi$, a direct calculation gives

$$\begin{aligned} \begin{aligned} \mathcal {L}^{[+2]}(\mathcal {L}^{[+2]}-2)\left(\Phi (\mathfrak {m},\mathfrak {m})\right)_s&= 2r^2\mathcal {L}_{-1}\mathcal {L}_0{{\,\mathrm{\textrm{div}}\,}}\hspace{-9.44443pt}/\ {{\,\mathrm{\textrm{div}}\,}}\hspace{-9.44443pt}/\ \Phi , \\ \mathcal {L}^{[+2]}(\mathcal {L}^{[+2]}-2)\left(\Phi (\mathfrak {m},\mathfrak {m})\right)_a&= 2ir^2\mathcal {L}_{-1}\mathcal {L}_0\mathop {\mathrm {\textrm{curl}}}\limits \hspace{-9.44443pt}/\ {{\,\mathrm{\textrm{div}}\,}}\hspace{-9.44443pt}/\ \Phi , \end{aligned} \end{aligned}$$

where we recall that $\mathfrak {m}=\frac{1}{r}\left({\partial }_{\vartheta }+\frac{i}{\sin {\vartheta }}{\partial }_\varphi \right)$.

For the next Lemma we recall the definition of a solution to the Teukolsky problem, Definition 2.1.

##### Lemma 2.20

The following two items are equivalent.

- $(\widetilde{\alpha }^{[+2]},\widetilde{\alpha }^{[-2]})$ are solutions to the Teukolsky problem
- both $(\widetilde{\alpha }^{[+2]}_s,\widetilde{\alpha }^{[-2]}_s)$ and $(\widetilde{\alpha }^{[+2]}_a,\widetilde{\alpha }^{[-2]}_a)$ are solutions to the Teukolsky problem

The following two items are equivalent.

- $(\widetilde{\alpha }^{[+2]},\widetilde{\alpha }^{[-2]})$ satisfy the Teukolsky–Starobinsky identities (1.3),
- $(\widetilde{\alpha }^{[+2]}_s,\widetilde{\alpha }^{[-2]}_s)$ satisfy the following identities
  2.55a $$\begin{aligned} w^2(w^{-1}L)^4(\widetilde{\alpha }^{[-2]}_s)^*&= +\mathcal {L}^{[+2]}(\mathcal {L}^{[+2]}-2)\widetilde{\alpha }^{[+2]}_s-12M{\partial }_t\widetilde{\alpha }^{[+2]}_s, \end{aligned}$$
  2.55b $$\begin{aligned} w^2(w^{-1}{\,\underline{L}})^4(\widetilde{\alpha }^{[+2]}_s)^*&= +\mathcal {L}^{[-2]}(\mathcal {L}^{[-2]}-2)\widetilde{\alpha }^{[-2]}_s+12M{\partial }_t\alpha _s^{[-2]}, \end{aligned}$$
  and $(\widetilde{\alpha }^{[+2]}_a,\widetilde{\alpha }^{[-2]}_a)$ satisfy the following identities
  2.56a $$\begin{aligned} w^2(w^{-1}L)^4(\widetilde{\alpha }^{[-2]}_a)^*&= -\mathcal {L}^{[+2]}(\mathcal {L}^{[+2]}-2)\widetilde{\alpha }^{[+2]}_a-12M{\partial }_t\widetilde{\alpha }^{[+2]}_a, \end{aligned}$$
  2.56b $$\begin{aligned} w^2(w^{-1}{\,\underline{L}})^4(\widetilde{\alpha }^{[+2]}_a)^*&= -\mathcal {L}^{[-2]}(\mathcal {L}^{[-2]}-2)\widetilde{\alpha }^{[-2]}_a+12M{\partial }_t\widetilde{\alpha }_a^{[-2]}. \end{aligned}$$

##### Proof

The first equivalence is immediate using that the composition of $\mathcal {C}$ and complex conjugation commutes with the Teukolsky equation. The second equivalence is a direct consequence of (2.53). $\square$

#### Regge–Wheeler Teukolsky–Starobinsky constraints

We have the following proposition which translates the Teukolsky–Starobinsky constraints into conditions on the Dirichlet and “Robin” Regge–Wheeler quantities.

##### Proposition 2.21

Let $\widetilde{\alpha }^{[\pm 2]}$ be two solutions of the Teukolsky problem (2.5) on $\{t^\star \ge t^\star _0\}$. Let $\Psi ^{[\pm 2]},\psi ^{[\pm 2]}$ be the Chandrasekhar transformations of $\widetilde{\alpha }^{[\pm 2]}$ and $\Psi ^D,\Psi ^R$ be the associated Regge–Wheeler quantities defined by (2.35). The following two items are equivalent.

- $\widetilde{\alpha }^{[+2]}_s$ and $\widetilde{\alpha }^{[-2]}_s$ satisfy the Teukolsky–Starobinsky relations (2.55),
- $$\Psi ^D_s=0$$.

The following two items are equivalent.

- $\widetilde{\alpha }^{[+2]}_a$ and $\widetilde{\alpha }^{[-2]}_a$ satisfy the Teukolsky–Starobinsky relations (2.56),
- $$\Psi ^R_a=0$$.

##### Proof

We start with the first equivalence. Using the definition of the Chandrasekhar transformation (1.5) and the definition (2.35) of $\Psi ^D$ and the formula (2.41), we have

$$\begin{aligned} (2.55)~&\Leftrightarrow {\left\{ \begin{array}{ll} \left(\mathcal {L}^{[+2]}(\mathcal {L}^{[+2]}-2) - 12M{\partial }_t\right)\widetilde{\alpha }^{[+2]}_s= w^2(w^{-1}L)^2\big (\Psi ^{[-2]}_s\big )^*\\ \left(\mathcal {L}^{[-2]}(\mathcal {L}^{[-2]}-2) + 12M{\partial }_t\right)\widetilde{\alpha }^{[-2]}_s= w^2(w^{-1}{\,\underline{L}})^2\big (\Psi ^{[+2]}_s\big )^*\end{array}\right. }\\&\Leftrightarrow {\left\{ \begin{array}{ll} \left(\mathcal {L}^{[+2]}(\mathcal {L}^{[+2]}-2) - 12M{\partial }_t\right)\widetilde{\alpha }^{[+2]}_s = w^2(w^{-1}L)^2\Psi _s^{[+2]} - w^2(w^{-1}L)^2\Psi _s^D\\ \left(\mathcal {L}^{[-2]}(\mathcal {L}^{[-2]}-2) + 12M{\partial }_t\right)\widetilde{\alpha }^{[-2]}_s = w^2(w^{-1}{\,\underline{L}})^2\Psi ^{[-2]}_s + w^2(w^{-1}{\,\underline{L}})^2\big (\Psi ^D_s\big )^*\end{array}\right. }\\&\Leftrightarrow {\left\{ \begin{array}{ll} \left(\mathcal {L}^{[+2]}(\mathcal {L}^{[+2]}-2) - 12M{\partial }_t\right)\widetilde{\alpha }^{[+2]}_s = w^2(w^{-1}L)^2(w^{-1}{\,\underline{L}})^2\widetilde{\alpha }^{[+2]}_s - w^2(w^{-1}L)^2\Psi ^D_s\\ \left(\mathcal {L}^{[-2]}(\mathcal {L}^{[-2]}-2) + 12M{\partial }_t\right)\widetilde{\alpha }^{[-2]}_s = w^2(w^{-1}{\,\underline{L}})^2(w^{-1}L)^2\widetilde{\alpha }^{[-2]}_s + w^2(w^{-1}{\,\underline{L}})^2\big (\Psi ^D_s\big )^*\end{array}\right. }\\&\Leftrightarrow {\left\{ \begin{array}{ll} 0 = w^2(w^{-1}L)^2\Psi ^D_s\\ 0 = w^2(w^{-1}{\,\underline{L}})^2\Psi ^D_s. \end{array}\right. } \end{aligned}$$

Using formula (2.42), we have

$$\begin{aligned} {\left\{ \begin{array}{ll} 0 = w^2(w^{-1}L)^2\Psi ^D_s\\ 0 = w^2(w^{-1}{\,\underline{L}})^2\Psi ^D_s \end{array}\right. }&\implies 0 = (w^{-1}{\,\underline{L}})^2(w^{-1}L)^2\Psi ^D_s = (w^{-1}L)^2(w^{-1}{\,\underline{L}})^2\Psi ^D_s \\&\implies 0 = \left(\mathcal {L}(\mathcal {L}-2) - 12M{\partial }_t\right)\Psi ^D_s = \left(\mathcal {L}(\mathcal {L}-2) + 12M{\partial }_t\right)\Psi ^D_s \\&\implies 0 = \Psi ^D_s, \end{aligned}$$

and the converse is evident. We turn to the second equivalence. Using again (1.5), (2.35) and (2.41), we have

2.57 $$\begin{aligned} \begin{aligned} \Psi ^R_a = 0 \quad \Leftrightarrow {\left\{ \begin{array}{ll} w^2(w^{-1}L)^2\Psi ^R_a= 0 \\ w^2(w^{-1}{\,\underline{L}})^2\Psi ^R_a = 0 \\ \end{array}\right. } \Leftrightarrow {\left\{ \begin{array}{ll} \left(\mathcal {L}^{[+2]}(\mathcal {L}^{[+2]}-2)-12M{\partial }_t\right)A^{[+2]}_a = 0 \\ \left(\mathcal {L}^{[-2]}(\mathcal {L}^{[-2]}-2)+12M{\partial }_t\right)A^{[-2]}_a = 0, \end{array}\right. } \end{aligned} \end{aligned}$$

with

$$\begin{aligned} A^{[+2]}_a&:= \left(\mathcal {L}^{[+2]}(\mathcal {L}^{[+2]}-2)+12M{\partial }_t\right)\widetilde{\alpha }^{[+2]}_a+ w^2(w^{-1}L)^2(\Psi ^{[-2]}_a)^*,\\ A^{[-2]}_a&:= \left(\mathcal {L}^{[-2]}(\mathcal {L}^{[-2]}-2)-12M{\partial }_t\right)\widetilde{\alpha }^{[-2]}_a + w^2(w^{-1}{\,\underline{L}})^2(\Psi ^{[+2]}_a)^*. \end{aligned}$$

Now, the boundary conditions (1.2), Teukolsky equations (2.5) and their consequences (see the proof of Proposition 2.2, in particular (2.18)), imply that

2.58 $$\begin{aligned} \begin{aligned} A^{[+2]}_a - (A^{[-2]}_a)^*&\xrightarrow {r\rightarrow +\infty }0,&{\,\underline{L}}(A^{[+2]}_a) - L(A^{[-2]}_a)^*&\xrightarrow {r\rightarrow +\infty }0. \end{aligned} \end{aligned}$$

Thus, combining (2.57) and (2.58), one has that

$$\begin{aligned}&\Psi ^R_a =0 \implies A^{[+2]}_a,~(A^{[-2]}_a)^*,~{\,\underline{L}}(A^{[+2]}_a),\\&L(A^{[-2]}_a)^*\xrightarrow {r\rightarrow +\infty }0 \implies A^{[+2]}_a = (A^{[-2]}_a)^*= 0, \end{aligned}$$

where the last step follows by unique continuation.^8^ The converse is immediate by (2.57) and this finishes the proof of the proposition. $\square$

##### Remark 2.22

Note that the proof of the first equivalence of Proposition 2.21 uses only the Teukolsky equations (2.5) and algebraic manipulations. In the proof of the second equivalence of Proposition 2.21, the first step (2.57) uses only the Teukolsky equations (2.5) and algebraic manipulations. In general (*i.e.* without imposing boundary conditions), it implies that the Teukolsky–Starobinsky identity (2.56) is equivalent to $\Psi ^R_a=0$ modulo “Teukolsky–Starobinsky modes” (see Definition 2.11).

From Proposition 2.21 we deduce the following corollary, which gives conditions so that the constructed Teukolsky quantities $\widetilde{\alpha }^{[\pm 2],r}$ satisfy the Teukolsky–Starobinsky relations.

##### Corollary 2.23

Let $\Psi ^D,\Psi ^R$ be two solutions of the decoupled Regge–Wheeler problem (2.36). Let $\Psi ^{[\pm 2]}$ be defined as in (2.38) and $\widetilde{\alpha }^{[+2],r}$ and $\widetilde{\alpha }^{[-2],r}$ be the reverse Chandrasekhar transformations of $\Psi ^{[\pm 2]}$, as defined in (1.8). Then, if $\Psi ^D_s=\Psi ^R_a=0$, $\widetilde{\alpha }^{[\pm 2,r]}$ satisfy the Teukolsky–Starobinsky relations (1.3).

##### Proof

From the formulas (2.42) and the definition of $\widetilde{\alpha }^{[\pm 2],r}$, we have that

$$\begin{aligned} \left(\mathcal {L}^2(\mathcal {L}-2)^2-144M^2{\partial }_t^2\right)\Psi ^{[+2]}&= (w^{-1}{\,\underline{L}})(w^{-1}{\,\underline{L}})\widetilde{\alpha }^{[+2],r}, \\ \left(\mathcal {L}^2(\mathcal {L}-2)^2-144M^2{\partial }_t^2\right)\Psi ^{[-2]}&= (w^{-1}L)(w^{-1}L)\widetilde{\alpha }^{[-2],r}. \end{aligned}$$

That is, $\left(\mathcal {L}^2(\mathcal {L}-2)^2-144M^2{\partial }_t^2\right)\Psi ^{[\pm 2]}$ are the Chandrasekhar transformations of $\widetilde{\alpha }^{[\pm 2],r}$ as defined in (1.5). The result of the corollary then follows from Proposition 2.21. $\square$

##### Remark 2.24

One may refine Corollary 2.23 to obtain an equivalence, *i.e.* that the Teukolsky–Starobinsky identities are satisfied if and only if $\Psi ^D_s,\Psi ^R_a$ are Regge–Wheeler Teukolsky–Starobinsky modes. We will not require this refinement here.

## The Main Theorem

Using the definitions of Sect. 2, we are ready to state the main theorem. We define the following energy-type norm for $\widetilde{\alpha }^{[\pm 2]}$ on $\Sigma _{t^\star }$

$$\begin{aligned} \begin{aligned} \textrm{E}^{\mathfrak {T},n}[\widetilde{\alpha }](t^\star )&:= \big \Vert {\partial }_{t^\star }\widetilde{\alpha }^{[+2]}\big \Vert _{H^{n-1}(\Sigma _{t^\star })}^2 + \big \Vert w^{-2}{\partial }_{t^\star }\widetilde{\alpha }^{[-2]}\big \Vert _{H^{n-1}(\Sigma _{t^\star })}^2+\big \Vert \widetilde{\alpha }^{[+2]}\big \Vert _{H^{n}(\Sigma _{t^\star })}^2\\&+ \big \Vert w^{-2}\widetilde{\alpha }^{[-2]}\big \Vert _{H^{n}(\Sigma _{t^\star })}^2, \end{aligned} \end{aligned}$$

for all integers $n\ge 1$ and where $L^2(\Sigma _{t^\star })$ and $H^n(\Sigma _{t^\star })$ as the standard spin-weighted Riemannian Sobolev spaces on the manifold $\Sigma _{t^\star }$ with metric induced by the conformal metric $r^{-2}g_{{M,k}}$. See for example [GH24b, Section 1.4] for more explicit definitions.

For the statement of the main theorem we recall in particular Definition 2.1.

### Theorem 3.1

(Inverse logarithmic decay: sharpness of the Teukolsky estimates) Let $t^\star _0\in {\mathbb R}$, $n>2$ and $p\in \mathbb {N}$. We have

3.1 $$\begin{aligned} \liminf _{t^\star \rightarrow +\infty }\sup _{\widetilde{\alpha }\in \mathcal {S}}\left(\log (t^\star -t^\star _0)^{2p} \frac{\textrm{E}^{\mathfrak {T},n}[\widetilde{\alpha }](t^\star )}{\textrm{E}^{\mathfrak {T},n}[\mathcal {L}^{p/2}\widetilde{\alpha }](t^\star _0)}\right)&> 0, \end{aligned}$$

where

$$\begin{aligned} \mathcal {S}&:= \left\{ \widetilde{\alpha }^{[\pm 2]}\text { solutions of the Teukolsky problem satisfying the Teukolsky--Starobinsky} \right. \\ &\quad \left. \text { constraints~(1.3)}\right\} \setminus \{0\}. \end{aligned}$$

Moreover, there exists an $H^{n+p}$-regular solution $\widetilde{\alpha }^{[\pm 2]}$ to the Teukolsky problem satisfying the Teukolsky–Starobinsky constraints (1.3), such that for all $\varepsilon >0$ we have

3.2 $$\begin{aligned} \log (t^\star -t^\star _0)^{2p+\varepsilon }\textrm{E}^{\mathfrak {T},n}[\widetilde{\alpha }](t^\star ) \xrightarrow {t^\star \rightarrow +\infty } + \infty \, . \end{aligned}$$

We refer the reader back to the introduction for a detailed interpretation and discussion of the theorem. The proof will be the content of Sects. 4 and 5. In the former, we construct quasimodes for the Regge–Wheeler system (Theorem 4.1). In the latter, we use the reverse Chandrasekhar transformations to produce quasimodes for the Teukolsky system and from those conclude the estimates (3.1) and (3.2).

## Quasimodes for the Regge–Wheeler Problem

In this section we construct quasimodes for the decoupled Regge–Wheeler problem (2.36). Quasimodes are real modes which are approximate solutions of the Regge–Wheeler problem (in the sense that the Regge–Wheeler equation (1.6) is satisfied up to a source error term). We will construct both Dirichlet and “Robin” quasimodes $\Psi ^D$ and $\Psi ^R$ for the Regge–Wheeler problem (2.36). Moreover, our construction will impose that respectively the symmetrised and anti-symmetrised parts of $\Psi ^D$ and $\Psi ^R$ vanish, *i.e.* $\Psi ^D_s=\Psi ^R_a=0$, which will guarantee that the associated Teukolsky quantities (constructed later in Sect. 5) will satisfy the Teukolsky–Starobinsky relations (see Sect. 2.8).

### The main result

The following theorem is the main result of the section.

#### Theorem 4.1

(Quasimodes for the Dirichlet and the Robin Regge–Wheeler problem). Let $M,k>0$ and $\delta >0$. There exists $\ell _{\textrm{QM}}(M,k,\delta )>0$ and two families of smooth spin-$+2$-weighted functions on $\mathcal {M}$

$$\begin{aligned} \Psi ^{D}_{m,\ell } := \Psi ^{D}_{\textrm{QM},m,\ell } + \Psi ^{D}_{{\textrm{Err}},m,\ell } \ \ \ \text {and} \ \ \ \Psi ^{R}_{m,\ell } := \Psi ^{R}_{\textrm{QM},m,\ell } + \Psi ^{R}_{{\textrm{Err}},m,\ell } \end{aligned}$$

with $\ell \ge \ell _{\textrm{QM}}$ and $0\le m \le \ell$, such that the following holds:

$$\Psi _{\textrm{QM},m,\ell }^{D/R}$$ **is a quasimode:**

1. The functions $\Psi ^{D/R}_{\textrm{QM},m,\ell }$ are (sums of) real modes, *i.e.* for all $\ell \ge \ell _\textrm{QM}$ there exists $\omega _\ell ^{D}, \omega ^{R}_{\ell }\in {\mathbb R}$^9^ such that
  4.1 $$\begin{aligned} \begin{aligned} \Psi ^D_{\textrm{QM},m,\ell }(t,r,{\vartheta },\varphi )&= e^{-i\omega _\ell ^D t} R_{\textrm{QM},\ell }^D(r) S_{m\ell }({\vartheta })e^{+im\varphi } - e^{+i\omega _\ell ^D t} R_{\textrm{QM},\ell }^D(r) S_{-m\ell }({\vartheta })e^{-im\varphi }, \\ \Psi ^{R}_{\textrm{QM},m,\ell }(t,r,{\vartheta },\varphi )&= e^{-i\omega _\ell ^R t} R_{\textrm{QM},\ell }^R(r) S_{m\ell }({\vartheta })e^{+im\varphi } + e^{+i\omega _\ell ^R t} R_{\textrm{QM},\ell }^R(r) S_{-m\ell }({\vartheta })e^{-im\varphi }, \end{aligned} \end{aligned}$$
  in the Dirichlet and the “Robin” case respectively, and where $R_{\textrm{QM},\ell }^{D/R}$ are real-valued functions.
2. The supports of $R^{D/R}_{\textrm{QM},\ell }$ are contained in $r\ge 3M$.
3. The mode frequencies $\omega _\ell ^{D}, \omega _\ell ^R$ both satisfy
  4.2 $$\begin{aligned} C^{-1}<\frac{(\omega ^{D/R}_\ell )^2}{\ell (\ell +1)}<C, \end{aligned}$$
  where $$C=C(M,k)>0$$.
4.
  $\Psi ^{D}_{\textrm{QM},m,\ell }$ satisfies the Dirichlet boundary condition at infinity (2.36ba),
  $\Psi ^R_{\textrm{QM},m,\ell }$ satisfies the “Robin” condition at infinity (2.36bb).
5. The supports of $\mathfrak {R}\Psi ^{D/R}_{\textrm{QM},m,\ell }$ are contained in $\{3M\le r \le 3M+\delta \}$.
6. For all $t^\star \ge t^\star _0$ and all $n\ge 0$,
  4.3 $$\begin{aligned} \big \Vert \Psi ^{D/R}_{\textrm{QM},m,\ell }\big \Vert _{H^n(\Sigma _{t^\star }\cap \{3M\le r \le 3M+\delta \})} \le Ce^{-C^{-1}\ell }\big \Vert \Psi ^{D/R}_{\textrm{QM},m,\ell }\big \Vert _{H^1(\Sigma _{t^\star _0})}, \end{aligned}$$
  where $C=C(M,k,n)>0$. In particular, we have
  4.4 $$\begin{aligned} \big \Vert \mathfrak {R}\Psi ^{D/R}_{\textrm{QM},m,\ell }\big \Vert _{H^n(\Sigma _{t^\star })}&\le Ce^{-C^{-1}\ell }\big \Vert \Psi ^{D/R}_{\textrm{QM},m,\ell }\big \Vert _{H^1(\Sigma _{t^\star _0})}, \end{aligned}$$
  for all $t^\star \ge t^\star _0$ and all $n\ge 0$, and where $C=C(M,k,n)>0$.
7. For all $t^\star \ge t^\star _0$ and all $n\ge 1$, we have
  4.5 $$\begin{aligned}&\left\Vert{\partial }_{t^\star }\Psi ^{D/R}_{\textrm{QM},m,\ell }\right\Vert_{H^{n-1}(\Sigma _{t^\star })} + \left\Vert\Psi ^{D/R}_{\textrm{QM},m,\ell }\right\Vert_{H^{n}(\Sigma _{t^\star })}\nonumber \\&\simeq _{M,k,n} \ell ^{n-1}\left(\left\Vert{\partial }_{t^\star }\Psi ^{D/R}_{\textrm{QM},m,\ell }\right\Vert_{L^2(\Sigma _{t^\star })} + \left\Vert\Psi ^{D/R}_{\textrm{QM},m,\ell }\right\Vert_{H^1(\Sigma _{t^\star })}\right), \end{aligned}$$
  and
  4.6 $$\begin{aligned} \left\Vert{\partial }_{t^\star }\Psi ^{D/R}_{\textrm{QM},m,\ell }\right\Vert_{H^{n-1}(\Sigma _{t^\star })} + \left\Vert\Psi ^{D/R}_{\textrm{QM},m,\ell }\right\Vert_{H^{n}(\Sigma _{t^\star })}&\simeq _{M,k,n} \left\Vert{\partial }_{t^\star }\Psi ^{D/R}_{\textrm{QM},m,\ell }\right\Vert_{H^{n-1}(\Sigma _{t^\star _0})}\nonumber \\&+ \left\Vert\Psi ^{D/R}_{\textrm{QM},m,\ell }\right\Vert_{H^{n}(\Sigma _{t^\star _0})}, \end{aligned}$$
  provided that $\ell$ is sufficiently large (depending on *M*, *k*, *n*).

$\Psi _{{\textrm{Err}},m,\ell }^{D/R}$ **is an error term:**

1. $\Psi ^{D/R}_{{\textrm{Err}},m,\ell }$ is the solution of the inhomogeneous Regge–Wheeler problem
  4.7a $$\begin{aligned} \begin{aligned} \mathfrak {R}\Psi ^{D/R}_{{\textrm{Err}},m,\ell }&= - \mathfrak {R}\Psi ^{D/R}_{\textrm{QM},m,\ell }, \end{aligned} \end{aligned}$$
  satisfying the Dirichlet boundary condition (2.36ba) in the $\Psi ^D$ case and the “Robin” boundary condition (2.36bb) in the $\Psi ^R$ case, and with initial data
  4.7b $$\begin{aligned} \Psi ^{D/R}_{{\textrm{Err}},m,\ell }\bigg |_{t^\star =t^\star _0}&= 0,&{\partial }_{t^\star }\Psi ^{D/R}_{{\textrm{Err}},m,\ell }\bigg |_{t^\star =t^\star _0}&= 0. \end{aligned}$$
2. We have
  4.8 $$\begin{aligned} \left\Vert{\partial }_{t^\star }\Psi ^{D/R}_{{\textrm{Err}},m,\ell }\right\Vert_{H^{n-1}(\Sigma _{t^\star })} + \left\Vert\Psi ^{D/R}_{{\textrm{Err}},m,\ell }\right\Vert_{H^n(\Sigma _{t^\star })}&\le C (1+t^\star ) e^{-C^{-1}\ell }\big \Vert \Psi ^{D/R}_{\textrm{QM},m,\ell }\big \Vert _{H^1(\Sigma _{t^\star _0})}, \end{aligned}$$
  for all $n\ge 1$ and all $t^\star \ge t^\star _0$ and with $C=C(M,k,n)>0$.

#### Remark 4.2

Note that by (4.7a) and item 4 above we have in particular that $\Psi ^{D}_{m, \ell }$ and $\Psi ^R_{m \ell }$ satisfy the Regge–Wheeler equation with boundary conditions  (2.36ba) and  (2.36bb) respectively.

#### Remark 4.3

The sums of the modes in definition (4.1) ensures that $\left(\Psi ^D_{\textrm{QM},m,\ell }\right)_s=\left(\Psi ^R_{\textrm{QM},m,\ell }\right)_a=0$ (see Definition 2.18). In turn, by the definition of $\Psi ^{D/R}_{{\textrm{Err}},m,\ell }$, this implies that

$$\begin{aligned} \left(\Psi ^D_{m,\ell }\right)_s=\left(\Psi ^R_{m,\ell }\right)_a= \left(\Psi ^D_{{\textrm{Err}},m,\ell }\right)_s=\left(\Psi ^R_{{\textrm{Err}},m,\ell }\right)_a = 0. \end{aligned}$$

That Teukolsky/Regge–Wheeler modes have to be summed to be acceptable quantities emanating from solutions to the system of gravitational perturbations is a direct consequence of a combination of the AdS boundary conditions and the Bianchi equations. See also Definition 1.5 and Remarks 1.6, 1.7 in [GH23].

The remaining subsections are dedicated to the proof of Theorem 4.1. Since the proof in the Dirichlet case is much simpler (and follows with very minor modifications the one in [HS14]), we will only prove Theorem 4.1 for $\Psi ^R_{m \ell }$. In the following we will thus drop all the superscripts “*R*” since no confusion is possible.

### Overview of the proof

We begin by introducing a truncated (at $r=3M$) 1-dimensional eigenvalue problem in Sect. 4.3, which is simply the Regge–Wheeler equation written down for a real mode solution with fixed real frequency $\omega$ and spherical harmonic $\ell ,m$, with Dirichlet boundary conditions imposed at $r=3M$ and the higher order Robin boundary condition at infinity. Solving this eigenvalue problem serves two purposes. First it produces the modes that will eventually be used in the construction of the quasimodes. Second, and more generally, one obtains a Hilbert basis of radial functions (with eigenvalue spectrum $\omega _{\ell ,n}$) which—using also the decomposition into spherical harmonics—can be employed to express every solution to the Regge–Wheeler equation on $r \ge 3M$ (with Dirichlet conditions at $r=3M$ and higher order Robin boundary conditions at infinity) in. This directly leads to a well-posedness result for the (truncated at $r=3M$) Regge–Wheeler equation, see Proposition 4.9. By domain of dependence, this well-posedness result can be easily upgraded to produce solutions on the whole of $\mathcal {M} \cap \{t^\star \ge t^\star _0\}$ from initial data posed on $t^\star =t^\star _0$, which we will do in Theorem 4.10. Armed with this we can finally embark on the proof of Theorem 4.1, which from this point onward proceeds just as for the linear wave equation [HS14]. We show that for sufficiently large $\ell$ the lowest mode ($m=0$) satisfies $\omega _\ell \sim \ell$ (Lemma 4.11). We pick this mode and smoothly cut it off to be identically zero for $r \le 3M$. The cut-off solution will now fail to satisfy the Regge–Wheeler equation but the error is exponential small by an Agmon estimate. Using Duhamel’s principle (this is where we need the well-posedness result) we finally construct an actual solution with slow decay in time.

#### Remark 4.4

The eigenvalue problem that we are about to solve is non-standard because of the higher-order “Robin” boundary condition (2.36bb). Namely, for Neumann or standard Robin boundary conditions, the eigenvalue problem is classically solved by incorporating these conditions in a weak formulation of the problem, see *e.g.* [Bré05, Section 8]. Here the higher-order boundary conditions involve time derivatives, *i.e.* the mode frequencies $\omega$, which are themselves the eigenvalues that we are looking for. Remarkably, we manage to include these in the weak formulation of the problem, see (4.10) and the minimisation problem (4.11).

### The truncated Regge–Wheeler eigenvalue problem

The idea is to obtain the radial part $R_{\textrm{QM},\ell }$ of the quasimode $\Psi _{\textrm{QM},m,\ell }$ as a solution to the following truncated “Robin” Regge–Wheeler eigenvalue problem.

#### Proposition 4.5

Let $\ell \ge 2$, $|m|\le \ell$, $\omega \in \mathbb {R}$ and $R : [r^\star _{3M},\pi /2]_{r^\star }\rightarrow \mathbb {R}$ be a smooth function, where $r^\star _{3M}=r^\star (r=3M)$. The mode

$$\begin{aligned} \Psi (t,r^\star ,{\vartheta },\varphi )&= e^{-i\omega t}R(r^\star )S_{m\ell }({\vartheta })e^{+im\varphi }, \end{aligned}$$

is a solution to the Regge–Wheeler equation (1.6) with Dirichlet boundary conditions at $r=3M$ and “Robin” boundary conditions (2.36bb) at infinity, if, and only if, *R* and $\omega$ are solutions to the following eigenvalue problem

4.9a $$\begin{aligned} \omega ^2R&= -R'' + w\left(\ell (\ell +1)-\frac{6M}{r}\right)R, \end{aligned}$$

with

4.9b $$\begin{aligned} \begin{aligned} R\left(r^\star _{3M}\right)&= 0,&\text {and} &  \left(-2\omega ^2R+ \frac{\ell (\ell +1)(\ell (\ell +1)-2)}{6M}{\partial }_{r^\star }R + k^2 \ell (\ell +1)R\right)\left(\frac{\pi }{2}\right)&= 0. \end{aligned} \end{aligned}$$

#### Proof

Direct computation. $$\square $$

We have the following weak formulation of the truncated “Robin” Regge–Wheeler eigenvalue problem (4.9). The proof is by integration by parts and is left to the reader.

#### Lemma 4.6

(Weak formulation of the “Robin” Regge–Wheeler eigenvalue problem). Let $\ell \ge 2$ and $\omega \in \mathbb {R}$. $R : [r^\star _{3M},\pi /2]_{r^\star }\rightarrow \mathbb {R}$ is a smooth solution to the truncated Robin Regge–Wheeler eigenvalue problem (4.9) if, and only if, for all $v\in C^\infty _c((r^\star _{3M},\pi /2])$,

4.10 $$\begin{aligned} \begin{aligned}&\int _{r^\star _{3M}}^{\frac{\pi }{2}}\left(R'v' + w\left(\ell (\ell +1)-\frac{6M}{r}\right)Rv\right) \,{\textrm{d}}r^\star + \frac{6Mk^2}{\ell (\ell +1)-2} \int _{r^\star _{3M}}^{\frac{\pi }{2}} \left(R'v + Rv'\right)\,{\textrm{d}}r^\star \\&\quad = \; \omega ^2\left(\int _{r^\star _{3M}}^{\frac{\pi }{2}}Rv \,{\textrm{d}}r^\star + \frac{12M}{\ell (\ell +1)(\ell (\ell +1)-2)} \int _{r^\star _{3M}}^{\frac{\pi }{2}} \left(R'v + Rv'\right)\,{\textrm{d}}r^\star \right). \end{aligned} \end{aligned}$$

Using the weak formulation of Lemma 4.6, we obtain the following diagonalisation result for the truncated “Robin” Regge–Wheeler eigenvalue problem.

#### Proposition 4.7

(Diagonalisation of the “Robin” Regge–Wheeler eigenvalue problem) The truncated “Robin” Regge–Wheeler eigenvalue problem (4.9) admits an Hilbert basis of (smooth) solutions $\left(R_{\textrm{QM},\ell ,n}\right)_{n\in \mathbb {N}}$ with eigenvalues $\omega ^2_{\ell ,n}$ on the real Hilbert space $H^1_0\left((r^\star _{3M},\pi /2]\right)$^10^ for the scalar product^11^

$$\begin{aligned} a(u,v)&:= \int _{r^\star _{3M}}^{\frac{\pi }{2}}\left(u'v' + w\left(\ell (\ell +1)-\frac{6M}{r}\right)uv\right) \,{\textrm{d}}r^\star \nonumber \\&\qquad + \frac{6Mk^2}{\ell (\ell +1)-2} \int _{r^\star _{3M}}^{\frac{\pi }{2}} \left(u'v + uv'\right)\,{\textrm{d}}r^\star . \end{aligned}$$

We denote by $\omega _\ell ^2=\omega ^2_{\ell ,0}$ the fundamental eigenvalue to the problem (4.9) and by $R_{\textrm{QM},\ell } = R_{\textrm{QM},\ell ,0}$ its associated eigenfunctions. $\omega _\ell ^2$ is given by the following twisted Rayleigh quotient

4.11 $$\begin{aligned} \begin{aligned} \omega _\ell ^2&:= \inf _{R\in H^1_0\left((r^\star _{3M},\pi /2]\right)\setminus \{0\}}\dfrac{{\int _{r^\star _{3M}}^{\frac{\pi }{2}}\left((R')^2 + w\left(\ell (\ell +1)-\frac{6M}{r}\right)R^2\right) \,{\textrm{d}}r^\star + \frac{6Mk^2}{\ell (\ell +1)-2}\left(R(\frac{\pi }{2})\right)^2}}{{\int _{r^\star _{3M}}^{\frac{\pi }{2}} R^2 \,{\textrm{d}}r^\star + \frac{12M}{\ell (\ell +1)(\ell (\ell +1)-2)}\left(R(\frac{\pi }{2})\right)^2}}, \end{aligned} \end{aligned}$$

and $R_{\textrm{QM},\ell }$ is a solution to the above minimisation problem.

#### Proof

Define the continuous, symmetric, positive, bilinear form *b* on $H^1_0\left((r^\star _{3M},\pi /2]\right)$ by

$$\begin{aligned} b(f,v)&:= \int _{r^\star _{3M}}^{\frac{\pi }{2}}fv \,{\textrm{d}}r^\star + \frac{12M}{\ell (\ell +1)(\ell (\ell +1)-2)} \int _{r^\star _{3M}}^{\frac{\pi }{2}} \left(f'v + fv'\right)\,{\textrm{d}}r^\star . \end{aligned}$$

By Riesz-Fréchet theorem (see [Bré05, Section V]), there exists a continuous linear operator $T:H^1_0\left((r^\star _{3M},\pi /2]\right) \rightarrow H^1_0\left((r^\star _{3M},\pi /2]\right)$ such that

4.12 $$\begin{aligned} \forall v\in H^1_0\left((r^\star _{3M},\pi /2]\right),\quad a((Tf),v) = b(f,v). \end{aligned}$$

Since *b* is positive and symmetric, the operator *T* is positive and symmetric with respect to *a*. Moreover, for $f\in H^1_0\left((r^\star _{3M},\pi /2]\right)$, (4.12) rewrites

4.13 $$\begin{aligned} \begin{aligned}&\int _{r^\star _{3M}}^{\frac{\pi }{2}}\left((Tf)'+ \frac{6Mk^2}{\ell (\ell +1)-2}(Tf) - \frac{12M}{\ell (\ell +1)(\ell (\ell +1)-2)} f \right) v' \, {\textrm{d}}r^\star \\&\quad = \int _{r^\star _{3M}}^{\frac{\pi }{2}}\underbrace{\left(f + \frac{12M}{\ell (\ell +1)(\ell (\ell +1)-2)} f' -w\left(\ell (\ell +1)-\frac{6M}{r}\right)(Tf) - \frac{6Mk^2}{\ell (\ell +1)-2}(Tf)'\right)}_{\in L^2((r^\star _{3M},\pi /2))} v \,{\textrm{d}}r^\star \end{aligned} \end{aligned}$$

for all $v\in H^1_0\left((r^\star _{3M},\pi /2]\right)$. Hence $(Tf)''$ is locally in $L^2$ and (4.9a) holds locally in $L^2$. But $(Tf) \in L^2((r^\star _{3M},\pi /2))$, thus, by (4.9a), $(Tf)''\in L^2((r^\star _{3M},\pi /2))$ and $\textrm{Im}(T) \subset H^2([r^\star _{3M},\pi /2])$. Hence, by Rellich-Kondrachov theorem (see [Bré05, Section IX]), *T* is compact. Therefore, by the spectral theorem, *T* is diagonalisable in an Hilbert basis for *a* and its maximal eigenvalue $\omega _\ell ^{-2}$ is obtained by (4.11) (see [Bré05, Section VI]). Bootstrapping the regularity of the eigenfunctions, one obtains that these are smooth functions on $[r^\star _{3M},\pi /2]$. Applying Lemma 4.6, this finishes the proof of the proposition. $\square$

#### Remark 4.8

We define the Hilbert space

4.14 $$\begin{aligned} \begin{aligned} \mathcal {H}&:= \bigg \{u \in H^3\left([r^\star _{3M},\pi /2]\right) \cap H^1_0\left((r^\star _{3M},\pi /2]\right):\\&\quad \quad \quad \quad \quad \quad \left[ 2u''-k^2\ell (\ell +1)u + \frac{(\ell -1)\ell (\ell +1)(\ell +2)}{6M}u'\right] \left( \frac{\pi }{2}\right) = 0 \bigg \}, \end{aligned} \end{aligned}$$

—which is a closed subspace of $H^3\left([r^\star _{3M},\pi /2]\right)$ –, and the scalar product

$$\begin{aligned} \widetilde{a}(u,v) := a(Lu,Lv) + \ell ^4a(u,v), \end{aligned}$$

with $Lu := -u''+w\left( \ell (\ell +1)-\frac{6M}{r}\right) u$—which defines a norm equivalent to the $H^3$-norm –. One can check that

- the eigenfunctions $R_{\textrm{QM},\ell ,n}$ obtained in Proposition 4.7 belong to $\mathcal {H}$ (see (4.9b)),
- $T(\mathcal {H})\subset \mathcal {H}$ and $T:\mathcal {H}\rightarrow \mathcal {H}$ is continuous and compact,
- the operator *T* of the proof of Proposition 4.7, restricted to $\mathcal {H}$, is self-adjoint for $\widetilde{a}$.

Thus, the Hilbert basis $(R_{\textrm{QM},\ell ,n})_{n\in \mathbb {N}}$ of $H^1_0\left((r^\star _{3M},\pi /2]\right)$ obtained in Proposition 4.7 is also a Hilbert basis of $\mathcal {H}$ for the scalar product $\widetilde{a}$ (in particular, it is dense in $\mathcal {H}$ for the $H^3$-topology).^12^ Note also that, for any function $u\in \mathcal {H}$, we have the following relations between the projections in $H^1_0$ and in $\mathcal {H}$

4.15 $$\begin{aligned} \widetilde{a}(u,R_{\textrm{QM},\ell ,n})&= \left(\omega _{\ell ,n}^4+\ell ^4\right)a(u,R_{\textrm{QM},\ell ,n}). \end{aligned}$$

Following the same ideas, defining the scalar product $(u,v)\mapsto a(L^pu,L^pv)+\ell ^{4p}a(u,v)$ on the Hilbert space $\mathcal {H}\cap H^{2p+1}\left([r^\star _{3M},\pi /2]\right)$, one can show that $(R_{\textrm{QM},\ell ,n})_{n\in \mathbb {N}}$ is a Hilbert basis of $\mathcal {H}\cap H^{2p+1}\left([r^\star _{3M},\pi /2]\right)$ for all $p\in \mathbb {N}$ for the above mentioned scalar product (in particular, dense for the $H^{2p+1}$-topology), and we have an analogous formula to (4.15).

### Well-posedness of the Regge–Wheeler problem

We can use the results of the previous section to obtain a well-posedness statement for the Regge–Wheeler problem for $\Psi ^R$ with the higher order boundary condition (2.36bb). We first state a well-posedness result for the truncated problem which is a consequence of Proposition 4.7:

#### Proposition 4.9

(Well-posedness of the truncated “Robin” problem). Let $t_0\in \mathbb {R}$. Let $(\Phi _0^R,\dot{\Phi }^R_0)$ be two smooth spin-weighted functions on $\{t=t_0\}\cap \{r\ge 3M\}$, regular at infinity (see Sect. 2.2), such that $\Phi _0^R|_{r=3M}=\dot{\Phi }_0^R|_{r=3M}=0$ and such that the following corner conditions are satisfied

4.16 $$\begin{aligned} \begin{aligned} 2{\partial }_{r^\star }^2\Phi _0^R - k^2\mathcal {L}\Phi _0^R + \frac{\mathcal {L}(\mathcal {L}-2)}{6M}{\partial }_{r^\star }\Phi _0^R&\xrightarrow {r\rightarrow +\infty } 0,\\ 2{\partial }_{r^\star }^2\dot{\Phi }_0^R - k^2\mathcal {L}\dot{\Phi }_0^R + \frac{\mathcal {L}(\mathcal {L}-2)}{6M}{\partial }_{r^\star }\dot{\Phi }_0^R&\xrightarrow {r\rightarrow +\infty } 0. \end{aligned} \end{aligned}$$

Let $\mathfrak {F}$ be a smooth spin-weighted function on $\{t\ge t_0\}\cap \{r\ge 3M\}$ such that $\mathfrak {F}(t)|_{r=3M}=0$ and $\mathfrak {F}(t)$ satisfies the boundary condition (4.16) at infinity. Then, there exists a unique smooth solution $\Phi ^R$ to the Regge–Wheeler equation (1.6) in the region $\{t\ge t_0\}\cap \{r\ge 3M\}$ satisfying Dirichlet conditions at $r=3M$ and the “Robin” boundary conditions (2.36bb) at infinity.

#### Proof

From the fact that $\left(S_{m\ell }({\vartheta })e^{-im\varphi }\right)_{\ell \ge 2, m\in \mathbb {Z}}$ is a Hilbert basis of the spin-$+2$-weighted complex $H^3$-functions on $\mathbb {S}^2$ and the fact that $\left(R_{\textrm{QM},\ell ,n}(r^\star )\right)_{\ell \ge 2, n\in \mathbb {N}}$ is a Hilbert basis of the real $H^1_0((r^\star _{3M},\pi /2])\cap H^{3}\left([r^\star _{3M},\pi /2]\right)$-functions satisfying the boundary condition of (4.14) (see Remark 4.8), we have that $\left(R_{\textrm{QM},\ell ,n}(r^\star ) S_{m\ell }({\vartheta })e^{-im\varphi }\right)_{\ell \ge 2, m\in \mathbb {Z}, n\in \mathbb {N}}$ is a Hilbert basis of the Hilbert space $\mathcal {H}$ of spin-$+2$-weighted $H^1_0\left( (r^\star _{3M},\pi /2]\times \mathbb {S}^2\right) \cap H^{3}\left( [r^\star _{3M},\pi /2]\times \mathbb {S}^2\right)$-functions which satisfy the boundary conditions

$$\begin{aligned} \left[2 {\partial }_{r^\star }^2\Phi - k^2\mathcal {L}\Phi + \frac{\mathcal {L}(\mathcal {L}-2)}{6M}{\partial }_{r^\star }\Phi \right]\left(\frac{\pi }{2}\right)=0. \end{aligned}$$

Denote by $\Phi _{0,\ell ,m,n}^R,\dot{\Phi }^R_{0,\ell ,m,n},\mathfrak {F}_{\ell ,m,n}(t)$ the projections of $\Phi ^R_0,\dot{\Phi }^R_0,\mathfrak {F}(t)$ on that Hilbert basis (by the regularity and corner compatibility assumptions on $\Phi _0^R,\dot{\Phi }_0^R$ and $\mathfrak {F}$, these quantities belong to the Hilbert space $\mathcal {H}$). We define

4.17 $$\begin{aligned} \begin{aligned} \Phi ^R(t,r^\star ,{\vartheta },\varphi )&:= \sum _{\ell \ge 2}\sum _{n\in \mathbb {N}}\sum _{m\in \mathbb {Z}} \bigg ( \Phi ^R_{0,\ell ,m,n} \cos \left(\omega _{\ell ,n}(t-t_0)\right) + \dot{\Phi }^R_{0,\ell ,m,n}\omega ^{-1}_{\ell ,n}\sin \left(\omega _{\ell ,n}(t-t_0)\right) \\&\quad \quad \quad + \frac{1}{\omega _{\ell ,n}}\int _{t_0}^{t}\sin \left(\omega _{\ell ,n}(t-t')\right)\mathfrak {F}_{\ell ,m,n}(t') \,{\textrm{d}}t'\bigg ) R_{\textrm{QM},\ell ,n}(r^\star ) S_{m\ell }({\vartheta })e^{-im\varphi }. \end{aligned} \end{aligned}$$

Note that (4.17) defines a function $\Phi ^R$ in $\mathcal {H}$ because $\Phi _0^R,\dot{\Phi }_0^R$ and $\mathfrak {F}$ belong to $\mathcal {H}$ and because the coefficients $\cos \left(\omega _{\ell ,n}(t-t_0)\right)$, $\omega ^{-1}_{\ell ,n}\sin \left(\omega _{\ell ,n}(t-t_0)\right)$ are bounded functions of $\ell ,n$. Applying the Regge–Wheeler operator to (4.17), it is clear, using (2.46) and (4.9a), that $\Phi ^R$ is a solution of the inhomogeneous Regge–Wheeler equation $\mathfrak {R}\Phi ^R = \mathfrak {F}$ and since $\Phi$ is in $\mathcal {H}$ it satisfies the Dirichlet condition at $r=3M$ and the desired “Robin” boundary condition (2.36bb) at infinity. Moreover, since $\left(R_{\textrm{QM},\ell ,n}(r^\star ) S_{m\ell }({\vartheta })e^{-im\varphi }\right)$ is a Hilbert basis in any more regular Sobolev space (see Remark 4.8), and since $\Phi ^R_0,\dot{\Phi }^R_0,\mathfrak {F}$ are assumed to be smooth, the sums in (4.17) converge in any more regular Sobolev space and (4.17) defines a smooth function.

That (4.17) gives the unique solution in $\mathcal {H}$ to the Regge–Wheeler problem can be seen by projection of the Regge–Wheeler equation onto the Hilbert basis $\left(R_{\textrm{QM},\ell ,n}(r^\star ) S_{m\ell }({\vartheta }) \right. \left. e^{-im\varphi }\right)$. This finishes the proof of the proposition. $\square$

We finally use a domain of dependence argument to establish well-posedness for the (non-truncated) problem we are actually interested in:

#### Theorem 4.10

(Well-posedness of the “Robin” problem). Let $t^\star _0\in \mathbb {R}$. Let $(\Psi ^R_0,\dot{\Psi }^R_0)$ be two smooth spin-weighted functions on $\Sigma _{t^\star _0}$, regular at the future event horizon, and such that we have the following corner compatibility condition

4.18 $$\begin{aligned} \begin{aligned} 2{\partial }_{r^\star }^2\Psi _0^R - k^2\mathcal {L}\Psi _0^R + \frac{\mathcal {L}(\mathcal {L}-2)}{6M}{\partial }_{r^\star }\Psi _0^R&\xrightarrow {r\rightarrow +\infty } 0,\\ 2{\partial }_{r^\star }^2\dot{\Psi }_0^R - k^2\mathcal {L}\dot{\Psi }_0^R + \frac{\mathcal {L}(\mathcal {L}-2)}{6M}{\partial }_{r^\star }\dot{\Psi }_0^R&\xrightarrow {r\rightarrow +\infty } 0. \end{aligned} \end{aligned}$$

Let $\mathfrak {F}$ be a smooth spin-weighted function on $\{t^\star \ge t^\star _0\}$ regular at the future event horizon and at infinity, and assume that $\mathfrak {F}(t)$ satisfies the corner condition at infinity (4.18). There exists a unique smooth spin-weighted function $\Psi ^R$ on $\{t^\star \ge t^\star _0\}$ which solves the inhomogeneous Regge–Wheeler equation

4.19 $$\begin{aligned} \mathfrak {R}\Psi ^R = \mathfrak {F}, \end{aligned}$$

is regular at the future event horizon, satisfies the “Robin” boundary conditions (2.36bb) at infinity and is such that $\Psi ^R|_{t^\star =t^\star _0} = \Psi ^R_0$ and ${\partial }_{t^\star }\Psi ^R|_{t^\star =t^\star _0}=\dot{\Psi }^R_0$. Moreover, we have for all $t^\star \ge t^\star _0$4.20where $S_{t^\star ,\infty }=\Sigma _{t^\star }\cap \mathcal {I}$, together with consistent, higher order estimates.

**Fig. 2:**
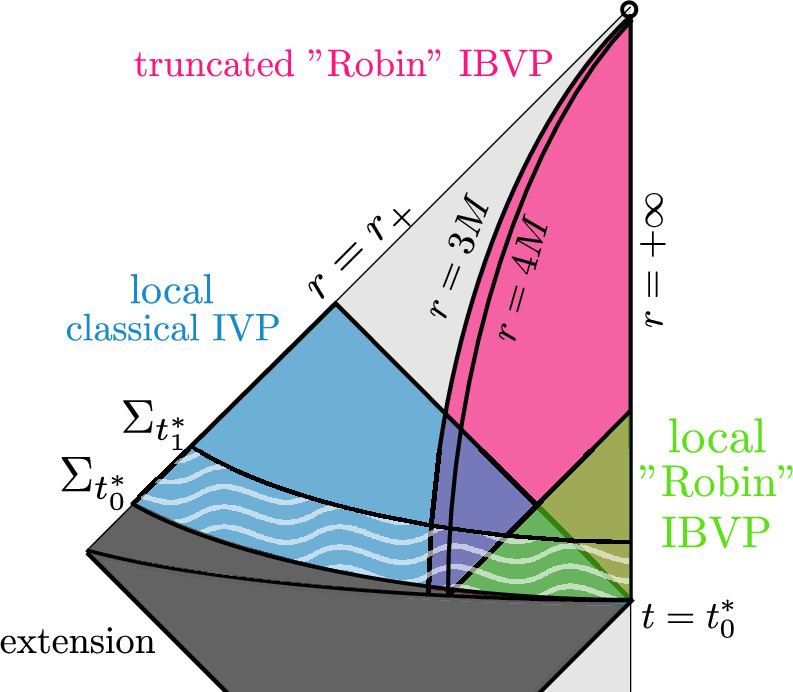
Global well-posedness of the "Robin" Regge–Wheeler problem

#### Proof of Theorem 4.10

The idea of the proof is to paste together a classical initial value well-posedness result in the domain of dependence of the initial slice and the truncated initial boundary value problem of Lemma 4.9. Let us define (see Fig. 2)

$$\begin{aligned} \mathcal {D}_{\textrm{blue}}&= D^+\left(\Sigma _{t^\star _0}\right),\\ \mathcal {D}_{\textrm{grey}}&= D^-\left(\Sigma _{t^\star _0}\cup \left(\mathcal {H}^+\cap \{t^\star \le t^\star _0\}\right)\right),\\ \mathcal {D}_{\textrm{pink}}&= \{t \ge t^\star _0\} \cap \{r\ge 3M\},\\ \mathcal {D}_{\textrm{green}}&= D^+\left(\left(\{t= t_0\}\cap \{r \ge 4M\}\right)\cup \{r=+\infty \}\right)\cap D^+\left(\Sigma _{t^\star _0}\cup \{r=+\infty \}\right). \end{aligned}$$

On $\mathcal {D}_{\textrm{blue}}$, there exists a unique smooth solution $\Psi ^R$ to the inhomogeneous Regge–Wheeler equation (4.19) with initial data $\Psi ^R_0,\dot{\Psi }^R_0$. Taking appropriate initial data on $\mathcal {H}^+\cap \{t^\star \le t^\star _0\}$, smoothly extending $\mathfrak {F}$ on $\mathcal {D}_{\textrm{grey}}$, and applying a (characteristic) classical well-posedness result, there exists a (non-unique) smooth solution $\widetilde{\Psi }$ to the inhomogeneous Regge–Wheeler equation (4.19) in $\mathcal {D}_{\textrm{grey}}$ which coincides with the initial data $\Psi _0^R,\dot{\Psi }_0^R$ on $\Sigma _{t^\star _0}$. Now, define $\widetilde{\widetilde{\Psi }} := \chi (r) \widetilde{\Psi }$, where $\chi$ is a smooth cut-off function such that $\chi |_{r\le 3M}=0$ and $\chi |_{r\ge 4M}=1$, and let $\widetilde{\widetilde{\Psi }}^R$ be the smooth solution on $\mathcal {D}_{\textrm{pink}}$ to the truncated “Robin” Regge–Wheeler problem of Proposition 4.9 with initial data given by $\widetilde{\widetilde{\Psi }}$ on $t=t^\star _0$ and with source term given by $\widetilde{\mathfrak {F}} = \chi (r)\mathfrak {F}$. We have that $\widetilde{\widetilde{\Psi }}^R$ coincides with the original initial data $\Psi _0^R,\dot{\Psi }_0^R$ on $\Sigma _{t^\star _0}\cap D^+\left(\left(\{t = t_0\}\cap \{r \ge 4M\}\right)\cup \{r=+\infty \}\right)$. Hence, by energy estimates, we have $\Psi ^R = \widetilde{\widetilde{\Psi }}^R$ in $\mathcal {D}_{\textrm{blue}}\cap \mathcal {D}_{\textrm{green}}$ and we can define $\Psi ^R := \widetilde{\widetilde{\Psi }}^R$ in $\mathcal {D}_{\textrm{green}}$. Let $t^\star _1 > t^\star _0$ be such that $\{t^\star _0 \le t^\star \le t^\star _1\}\subset \mathcal {D}_{\textrm{blue}} \cup \mathcal {D}_{\textrm{green}}$ (see Fig. 2). Then, $\Psi ^R$ is a smooth solution to the inhomogeneous Regge–Wheeler equation with “Robin” boundary conditions in $\{t^\star _0\le t^\star \le t^\star _1\}$. Having established existence of a smooth solution, we can apply the energy and red-shift estimates of [GH24b], which immediately give the uniqueness and establish that (4.20) is satisfied in that region, together with consistent higher order estimates. By a continuity argument, the solution exists in the full domain $\{t^\star \ge t^\star _0\}$ and (4.20) holds globally. This finishes the proof of the theorem.

### Proof of Theorem 4.1

#### Localisation of the fundamental mode (lowest eigenvalue)

We first choose $\ell$ large enough so that the fundamental eigenvalue satisfies $\omega _\ell \sim \ell$.

##### Lemma 4.11

Let $\eta >0$. There exists $\ell$ sufficiently large, such that the fundamental eigenvalue $\omega _\ell$ defined in Proposition 4.7 satisfies

4.21 $$\begin{aligned} \frac{k^2}{2} \le \frac{\omega _{\ell }^2}{\ell (\ell +1)} \le k^2+\eta . \end{aligned}$$

##### Proof

From (4.11) and the fact that the infimum is assumed by Proposition 4.7, we have

$$\begin{aligned} 0&= \int _{r^\star _{3M}}^{\frac{\pi }{2}} \left((R'_{\textrm{QM},\ell })^2 + \left(w\left(\ell (\ell +1)-\frac{6M}{r}\right)-\omega _\ell ^2\right)R_{\textrm{QM},\ell }^2\right) \,{\textrm{d}}r^\star \\&\quad + \left(\frac{6Mk^2}{\ell (\ell +1)-2}-\frac{12M\omega _\ell ^2}{\ell (\ell +1)(\ell (\ell +1)-2)}\right)\left(R_{\textrm{QM},\ell }(\frac{\pi }{2})\right)^2. \end{aligned}$$

Hence, we must have that

4.22 $$\begin{aligned} \omega _\ell ^2&\ge \inf _{[r^\star _{3M},\pi /2]} \left(w\left(\ell (\ell +1)-\frac{6M}{r}\right)\right),&\text {or} &  \frac{12M\omega _\ell ^2}{\ell (\ell +1)(\ell (\ell +1)-2)}&\ge \frac{6Mk^2}{\ell (\ell +1)-2}. \end{aligned}$$

For $\ell \ge 2$, we have that

$$\begin{aligned} \inf _{[r^\star _{3M},\pi /2]} \left(w\left(\ell (\ell +1)-\frac{6M}{r}\right)\right) \ge k^2(\ell (\ell +1)-2) \ge \frac{k^2}{2} \ell (\ell +1). \end{aligned}$$

Hence, either of the above two conditions in (4.22) yields the lower bound in (4.21). Let $r^\star _0 \in (r^\star _{3M},\pi /2)$. Taking *R* in (4.11) to be the piece wise affine function such that $R(r^\star )=0$ for $r^\star \in (r^\star _{3M},r^\star _0)$ and $R(\pi /2) = 1$, and using that *w* is decreasing on $(r^\star _{3M},\pi /2)$, we deduce (from the characterisation of the eigenvalue as the infimum) that

$$\begin{aligned} \omega _\ell ^2 \le w(r^\star _0)\ell (\ell +1) + o_{\ell \rightarrow +\infty }(\ell ^2). \end{aligned}$$

Using that $w(\pi /2) = k^2$, the upper bound in (4.21) follows and this concludes the proof of the lemma. $\square$

##### Corollary 4.12

Using (4.2) and (4.11), and absorbing the boundary term by a trace estimate, we have

4.23 $$\begin{aligned} \int _{r^\star _{3M}}^{\frac{\pi }{2}} \left(\omega _\ell ^2|R_{\textrm{QM},\ell }|^2 + \left(R'_{\textrm{QM},\ell }\right)^2 +\ell ^2|R_{\textrm{QM},\ell }|^2\right)\, {\textrm{d}}r^\star \simeq _{M,k}\ell ^2\int _{r^\star _{3M}}^{\frac{\pi }{2}}|R_{\textrm{QM},\ell }|^2\,{\textrm{d}}r^\star , \end{aligned}$$

for $\ell$ sufficiently large depending on *M*, *k*.

#### Estimating the energy in the classically forbidden region

We have the following localisation property, which can be paraphrased by saying that the energy of the mode is exponentially small near $r=3M$. The result is proven from Agmon-type estimates (see [HS14, Section 5] and (4.26) below).

##### Lemma 4.13

Let $r^\star _0\in (r^\star _{3M},\pi /2)$. There exists $C=C(M,k,r^\star _0)>0$, such that for $\ell$ sufficiently large, we have

4.24 $$\begin{aligned}&\int _{r^\star _{3M}}^{r^\star _0}\left((R_{\textrm{QM},\ell }')^2 + \ell (\ell +1)R_{\textrm{QM},\ell }^2\right) \,{\textrm{d}}r^\star \nonumber \\&\qquad \le C e^{-C^{-1}\ell } \int _{r^\star _{3M}}^{\frac{\pi }{2}}\left((R_{\textrm{QM},\ell }')^2 + \ell (\ell +1)R_{\textrm{QM},\ell }^2\right) \,{\textrm{d}}r^\star . \end{aligned}$$

##### Proof

Let $r^\star _0\in (r^\star _{3M},\pi /2)$. Let $\delta >0$ such that $r^\star _0+2\delta <\pi /2$ and $\eta >0$ such that

4.25 $$\begin{aligned} k^2+3\eta < w(r^\star _0+2\delta ). \end{aligned}$$

Define a function $\phi$ such that $\phi (\pi /2)=0$ and

$$\begin{aligned} \phi '&:= -\sqrt{\ell (\ell +1)\eta }\chi , \end{aligned}$$

with $0\le \chi \le 1$ a smooth function such that $\chi = 1$ on $(r^\star _{3M},r^\star _0+\delta )$ and $\chi =0$ on $(r^\star _0+2\delta ,\pi /2)$. Multiplying (4.9) by $e^{2\phi }$ and integrating by parts, we have

4.26 $$\begin{aligned} \begin{aligned}&\int _{r^\star _{3M}}^{\frac{\pi }{2}} \left(\left(\left(R_{\textrm{QM},\ell }e^{\phi }\right)'\right)^2 + \left((V -\omega _\ell ^2) - \left(\phi '\right)^2\right)R_{\textrm{QM},\ell }^2e^{2\phi } \right)\,{\textrm{d}}r^\star \\&\quad = \; \left(\frac{12M\omega _\ell ^2}{\ell (\ell +1)(\ell (\ell +1)-2)} - \frac{6Mk^2}{\ell (\ell +1)-2}\right)\left(R_{\textrm{QM},\ell }^2e^{2\phi }\right)\left(\frac{\pi }{2}\right). \end{aligned} \end{aligned}$$

Using the definition of $\phi$, (4.21), (4.25), and that *w* is a decreasing function on $(r^\star _{3M},\pi /2)$, we have

4.27 $$\begin{aligned} \begin{aligned} (V -\omega _\ell ^2) - \left(\phi '\right)^2&= \ell (\ell +1)\left(w - \frac{\omega _\ell ^2}{\ell (\ell +1)} - \eta \chi ^2\right) - w \frac{6M}{r}\\&\ge \ell (\ell +1)\left(w(r^\star _0+2\delta ) - k^2 - 2\eta \right) - 2w(3M)\\&\ge \ell (\ell +1)\eta - 2w(3M) \\&\ge \frac{1}{2}\ell (\ell +1)\eta , \end{aligned} \end{aligned}$$

for all $r^\star \in (r^\star _{3M},r^\star _0+2\delta )$ and where the second and last lines hold provided that $\ell$ is taken sufficiently large. Moreover, from the definition of $\phi$, we have

4.28 $$\begin{aligned} \begin{aligned} \phi (r^\star )&\ge \delta \sqrt{\ell (\ell +1)\eta }, \end{aligned} \end{aligned}$$

for all $r^\star \in (r^\star _{3M},r^\star _0)$. Applying now (4.26), using (4.27) and (4.28), we obtain

4.29 $$\begin{aligned} \begin{aligned}&\ell (\ell +1) e^{2\delta \sqrt{\ell (\ell +1)\eta }} \int _{r^\star _{3M}}^{r^\star _0} R_{\textrm{QM},\ell }^2 \,{\textrm{d}}r^\star \\&\quad \le 2\eta ^{-1} \int _{r^\star _{3M}}^{r^\star _0+2\delta }\left((V -\omega _\ell ^2) - \left(\phi '\right)^2\right)R_{\textrm{QM},\ell }^2e^{2\phi }\,{\textrm{d}}r^\star \\&\quad \le 2\eta ^{-1}\int _{r^\star _0+2\delta }^{\frac{\pi }{2}} (V -\omega _\ell ^2)R_{\textrm{QM},\ell }^2\,{\textrm{d}}r^\star \\&\qquad + 2\eta ^{-1}\left(\frac{12M\omega _\ell ^2}{\ell (\ell +1)(\ell (\ell +1)-2)} - \frac{6Mk^2}{\ell (\ell +1)-2}\right)R_{\textrm{QM},\ell }^2\left(\frac{\pi }{2}\right) \\&\quad \le 4\eta ^{-1}\int _{r^\star _{3M}}^{\frac{\pi }{2}} \left(\left(R_{\textrm{QM},\ell }'\right)^2 + (V -\omega _\ell ^2)R_{\textrm{QM},\ell }^2 \right)\,{\textrm{d}}r^\star . \end{aligned} \end{aligned}$$

Re-using (4.26) and plugging (4.29), we get

$$\begin{aligned}&e^{2\delta \sqrt{\ell (\ell +1)\eta }} \int _{r^\star _{3M}}^{r^\star _0} \left(\left(R_{\textrm{QM},\ell }'\right)^2 + \ell (\ell +1) R_{\textrm{QM},\ell }^2 \right)\,{\textrm{d}}r^\star \\&\quad \lesssim \eta ^{-1}\int _{r^\star _{3M}}^{\frac{\pi }{2}} \left(\left(R_{\textrm{QM},\ell }'\right)^2 + (V -\omega _\ell ^2)R_{\textrm{QM},\ell }^2 \right)\,{\textrm{d}}r^\star , \end{aligned}$$

and this finishes the proof of the lemma. $\square$

#### Completing the proof

We can now complete the proof of Theorem 4.1. We define

4.30 $$\begin{aligned} \widetilde{\Psi }_{\textrm{QM},m,\ell }(t,r^\star ,{\vartheta },\varphi )&:= e^{-i\omega _\ell t}R_{\textrm{QM},\ell }(r^\star )S_{m\ell }({\vartheta })e^{+im\varphi } \nonumber \\ &\quad + e^{+i\omega _\ell t}R_{\textrm{QM},\ell }(r^\star )S_{-m\ell }({\vartheta })e^{-im\varphi }, \end{aligned}$$

where $\omega _\ell$ and $R_{\textrm{QM},\ell }$ are the fundamental eigenvalue and eigenvector to the boundary value problem (4.9) from Proposition 4.7 and $\ell$ is sufficiently large so that in particular Lemma 4.11 applies. Define

$$\begin{aligned} \Psi _{\textrm{QM},m,\ell }&:= \chi (r^\star )\widetilde{\Psi }_{\textrm{QM},\ell }, \end{aligned}$$

where $\chi$ is a smooth cut-off function satisfying $\chi \big |_{r\le 3M}=0$ and $\chi \big |_{r\ge 3M+\delta }=1$. Items 1, 2, 4 and 5 for the quasimode part $\Psi _{\textrm{QM},m,\ell }$ listed in Theorem 4.1 are then immediate consequences of the definitions above. Item 3 follows from Lemma 4.11. Item 6 follows from Lemma 4.13, Item 7 from Corollary 4.12 (using the relation (4.9) to replace higher spatial derivatives in the Sobolev norms and $\omega _\ell \sim \ell$ for the time derivatives) and the fact that the solutions are periodic in time.^13^ Finally, that $\Psi _{{\textrm{Err}},m,\ell }$ can be defined as solution to (4.7a) and satisfies the estimate (4.8) is a direct consequence of the well-posedness result of Theorem 4.10 (in particular the inhomogeneous estimate (4.20) and the bounds (4.4) on the source term. This finishes the proof of Theorem 4.1.

## Quasimodes for the Teukolsky Problem

In this section we prove the main theorem, Theorem 3.1.

### Estimating the reverse Chandrasekhar transformations

We first define and control the reverse Chandrasekhar transformations (see (1.8)) of the quasimodes and error terms for the Regge–Wheeler equation constructed in Theorem 4.1. Let therefore $\Psi ^D_{m,\ell }$ or $\Psi ^R_{m,\ell }$ be a quantity as constructed in Theorem 4.1. In the Dirichlet case, we define

5.2 $$\begin{aligned} \begin{aligned} \Psi ^{[+2]}_{\textrm{QM},m,\ell }&:= \Psi ^D_{\textrm{QM},m,\ell } - \frac{12M}{\mathcal {L}(\mathcal {L}-2)}{\partial }_t\Psi ^D_{\textrm{QM},m,\ell } , \\ \Psi ^{[+2]}_{{\textrm{Err}},m,\ell }&:= \Psi ^D_{{\textrm{Err}},m,\ell } - \frac{12M}{\mathcal {L}(\mathcal {L}-2)}{\partial }_t\Psi ^D_{{\textrm{Err}},m,\ell }, \\ \Psi ^{[-2]}_{\textrm{QM},m,\ell }&:= -\big (\Psi ^D_{\textrm{QM},m,\ell }\big )^*- \left(\frac{12M}{\mathcal {L}(\mathcal {L}-2)}{\partial }_t\Psi ^D_{\textrm{QM},m,\ell }\right)^*, \\ \Psi ^{[-2]}_{{\textrm{Err}},m,\ell }&:= -\big (\Psi ^D_{{\textrm{Err}},m,\ell }\big )^*- \left(\frac{12M}{\mathcal {L}(\mathcal {L}-2)}{\partial }_t\Psi ^D_{{\textrm{Err}},m,\ell }\right)^*, \end{aligned} \end{aligned}$$

and in the “Robin” case

4.31 $$\begin{aligned} \begin{aligned} \Psi ^{[+2]}_{\textrm{QM},m,\ell }&:= \Psi ^R_{\textrm{QM},m,\ell },&\Psi ^{[+2]}_{{\textrm{Err}},m,\ell }&:= \Psi ^R_{{\textrm{Err}},m,\ell }, \\ \Psi ^{[-2]}_{\textrm{QM},m,\ell }&:= \big (\Psi ^R_{\textrm{QM},m,\ell }\big )^*,&\Psi ^{[-2]}_{{\textrm{Err}},m,\ell }&:= \big (\Psi ^R_{{\textrm{Err}},m,\ell }\big )^*. \end{aligned} \end{aligned}$$

Now, in both cases, we can define

5.3 $$\begin{aligned} \begin{aligned} \widetilde{\alpha }^{[+2]}_{\textrm{QM},m,\ell }&:= w^2\left(\mathcal {L}(\mathcal {L}-2)+12M{\partial }_{t^\star }\right)(w^{-1}L)^2\Psi ^{[+2]}_{\textrm{QM},m,\ell }, \\ \widetilde{\alpha }^{[+2]}_{{\textrm{Err}},m,\ell }&:= w^2\left(\mathcal {L}(\mathcal {L}-2)+12M{\partial }_{t^\star }\right)(w^{-1}L)^2\Psi ^{[+2]}_{{\textrm{Err}},m,\ell },\\ \widetilde{\alpha }^{[-2]}_{\textrm{QM},m,\ell }&:= w^2\left(\mathcal {L}(\mathcal {L}-2)-12M{\partial }_{t^\star }\right)(w^{-1}{\,\underline{L}})^2\Psi ^{[-2]}_{\textrm{QM},m,\ell }, \\ \widetilde{\alpha }^{[-2]}_{{\textrm{Err}},m,\ell }&:= w^2\left(\mathcal {L}(\mathcal {L}-2)-12M{\partial }_{t^\star }\right)(w^{-1}{\,\underline{L}})^2\Psi ^{[-2]}_{{\textrm{Err}},m,\ell }. \end{aligned} \end{aligned}$$

By construction, the quantities $\widetilde{\alpha }^{[\pm 2]}_{\textrm{QM},m,\ell }$ (and $\Psi ^{[\pm 2]}_{\textrm{QM},m,\ell }$) are sums of real mode with frequency $\pm \omega _\ell$. Moreover, by Proposition 2.7, the quantities $\Psi ^{[\pm 2]} = \Psi ^{[\pm 2]}_{\textrm{QM},m,\ell } + \Psi ^{[\pm 2]}_{{\textrm{Err}},m,\ell }$ are solutions to the (homogeneous) Regge–Wheeler problem (Definition 2.3), and, by Proposition 2.4, the quantities $\widetilde{\alpha }^{[\pm 2]} = \widetilde{\alpha }^{[\pm 2]}_{\textrm{QM},m,\ell }+\widetilde{\alpha }^{[\pm 2]}_{{\textrm{Err}},m,\ell }$ are solutions to the homogeneous Teukolsky problem (Definition 2.1). We have the following control of the error term:

#### Lemma 5.1

For all $\ell \ge \ell _{\textrm{QM}}$, $0 \le m \le \ell$, and all $n\ge 1$, we have on the initial slice $\Sigma _{t^\star _0}$

5.4 $$\begin{aligned} \begin{aligned} \textrm{E}^{\mathfrak {T},n}[\widetilde{\alpha }_{{\textrm{Err}},m,\ell }](t^\star _0)&\le Ce^{-C^{-1}\ell } \big \Vert \Psi _{\textrm{QM},m,\ell }^{D/R}\big \Vert _{H^1(\Sigma _{t^\star _0})}, \end{aligned} \end{aligned}$$

where $C=C(M,k,n)>0$. For all $n\ge 1$, we have on all truncated slices $\Sigma _{t^\star }\cap \{r\ge 3M\}$ with $t^\star \ge t^\star _0$,

5.5 $$\begin{aligned} \begin{aligned} \textrm{E}^{\mathfrak {T},n}_{r\ge 3M}[\widetilde{\alpha }_{{\textrm{Err}},m,\ell }](t^\star )&\le C(1+t^\star )e^{-C^{-1}\ell } \big \Vert \Psi _{\textrm{QM},m,\ell }^{D/R}\big \Vert _{H^1(\Sigma _{t^\star _0})}, \end{aligned} \end{aligned}$$

where $C=C(M,k,n)>0$ and where here and in the following, $\textrm{E}^{\mathfrak {T},n}_{r\ge R}[\Phi ](t^\star )$ is the same energy norm as defined in Sect. 3, but where the integrals are only taken on $\Sigma _{t^\star }\cap \{r\ge R\}$.

#### Proof

This follows directly from the definitions (5.1), (5.2) and (5.3) and the estimates (4.8). $\square$

We also collect the following estimates on the energy norms of the quasimodes $\widetilde{\alpha }^{[\pm 2]}_{\textrm{QM},m,\ell }$.

#### Lemma 5.2

For all $n\ge 1$ and all $t^\star \ge t^\star _0$, we have

5.6 $$\begin{aligned} C^{-1} \ell ^{2n+2} \big \Vert \Psi ^{D/R}_{\textrm{QM},m,\ell }\big \Vert _{H^1(\Sigma _{t^\star _0})}^2 \le \textrm{E}^{\mathfrak {T},n}[\widetilde{\alpha }_{\textrm{QM},m,\ell }](t^\star ) \le C \ell ^{2n+2} \big \Vert \Psi ^{D/R}_{\textrm{QM},m,\ell }\big \Vert ^2_{H^1(\Sigma _{t^\star _0})}, \end{aligned}$$

with $C=C(M,k,n)>0$ and for $\ell$ sufficiently large (depending on *M*, *k*, *n*).

#### Proof

Let us first show that

5.7 $$\begin{aligned}&\left\Vert{\partial }_{t^\star }^2\Psi ^{D/R}_{\textrm{QM},m,\ell }\right\Vert^2_{L^2(\Sigma _{t^\star })} + \left\Vert{\partial }_{t^\star }\Psi ^{D/R}_{\textrm{QM},m,\ell }\right\Vert^2_{H^1(\Sigma _{t^\star })} \nonumber \\&\qquad + \left\Vert\Psi ^{D/R}_{\textrm{QM},m,\ell }\right\Vert^2_{H^2(\Sigma _{t^\star })} \simeq _{M,k} \int _{\Sigma _{t^\star }}|{\,\underline{L}}{\,\underline{L}}\Psi ^{D/R}_{\textrm{QM},m,\ell }|^2. \end{aligned}$$

We prove (5.7) in the “Robin” case but the proof in the Dirichlet case follows along the same lines. Because of the localisation of $\Psi ^R_{\textrm{QM},m,\ell }$ in $r\ge 3M$, we can ignore the *w* weights. Using $\underline{L} = \partial _t - \partial _{r^\star }= \partial _t - \frac{\Delta }{r^2} \partial _{r}$, the definition of $\Psi ^R_{\textrm{QM},m,\ell }$ from the proof of Theorem 4.1 and that $R_{\textrm{QM},\ell }$ are real-valued functions (see Theorem 4.1 and its proof), we have

5.8 $$\begin{aligned} \begin{aligned} \int _{\Sigma _{t^\star }}|{\,\underline{L}}{\,\underline{L}}\Psi ^R_{\textrm{QM},m,\ell }|^2&\simeq _{M,k} \int _{-\infty }^{\frac{\pi }{2}} \left|-\omega _\ell ^2\chi R_{\textrm{QM},\ell } -2 i\omega _{\ell } \left(\chi R_{\textrm{QM},\ell }\right)' + \left(\chi R_{\textrm{QM},\ell }\right)''\right|^2 \,{\textrm{d}}r^\star , \\ \simeq _{M,k}&\int _{-\infty }^{\frac{\pi }{2}}\left(\left|(\chi R_{\textrm{QM},\ell })'' - \chi \omega _\ell ^2 R_{\textrm{QM},\ell }\right|^2 + 4 |\omega _{\ell }|^2\left|\left(\chi R_{\textrm{QM},\ell }\right)'\right|^2\right) \,{\textrm{d}}r^\star . \end{aligned} \end{aligned}$$

Using (4.9), we have

5.9 $$\begin{aligned} \begin{aligned}&\int _{r^\star _{3M}}^{\frac{\pi }{2}}\left(\left|R''_{\textrm{QM},\ell } - \omega _\ell ^2 R_{\textrm{QM},\ell }\right|^2 + 4 |\omega _{\ell }|^2\left|R'_{\textrm{QM},\ell }\right|^2\right)\,{\textrm{d}}r^\star \\&\quad = \int _{r^\star _{3M}}^{\frac{\pi }{2}} \left(\left|V-2\omega _\ell ^2\right|^2 |R_{\textrm{QM},\ell }|^2 + 4|\omega _{\ell }|^2 \left|R'_{\textrm{QM},\ell }\right|^2\right)\,{\textrm{d}}r^\star . \end{aligned} \end{aligned}$$

Using (4.11), we have

5.10 $$\begin{aligned} \begin{aligned} \int _{r^\star _{3M}}^{\frac{\pi }{2}} \left|R_{\textrm{QM},\ell }'\right|^2 \,{\textrm{d}}r^\star&= \int _{r^\star _{3M}}^{\frac{\pi }{2}} (\omega _\ell ^2-V) \left|R_{\textrm{QM},\ell }\right|^2 \,{\textrm{d}}r^\star \\&\quad + \left(\frac{12M\omega _\ell ^2}{\ell (\ell +1)(\ell (\ell +1)-2)}-\frac{6Mk^2}{\ell (\ell +1)-2}\right)\left|R_{\textrm{QM},\ell }\left(\frac{\pi }{2}\right)\right|^2. \end{aligned} \end{aligned}$$

The identity (5.10) yields in particular

5.11 $$\begin{aligned} \begin{aligned}&\int _{r^\star _{3M}}^{\frac{\pi }{2}} \left|V-2\omega _\ell ^2\right|^2 |R_{\textrm{QM},\ell }|^2\,{\textrm{d}}r^\star \\&\quad = \int _{r^\star _{3M}}^{\frac{\pi }{2}} V^2 |R_{\textrm{QM},\ell }|^2\,{\textrm{d}}r^\star + \int _{r^\star _{3M}}^{\frac{\pi }{2}} 4\omega _\ell ^2(\omega _{\ell }^2-V) |R_{\textrm{QM},\ell }|^2\,{\textrm{d}}r^\star \\&\quad = \int _{r^\star _{3M}}^{\frac{\pi }{2}} V^2 |R_{\textrm{QM},\ell }|^2\,{\textrm{d}}r^\star + 4\omega _\ell ^2 \int _{r^\star _{3M}}^{\frac{\pi }{2}} \left|R'_{\textrm{QM},\ell }\right|^2\,{\textrm{d}}r^\star \\&\qquad + 4\omega _\ell ^2\left(\frac{6Mk^2}{\ell (\ell +1)-2}-\frac{12M\omega _\ell ^2}{\ell (\ell +1)(\ell (\ell +1)-2)}\right)\left|R_{\textrm{QM},\ell }\left(\frac{\pi }{2}\right)\right|^2. \end{aligned} \end{aligned}$$

Thus, combining (5.9) and (5.11), using (4.2), (4.9) and a trace estimate for the boundary term, gives

5.12 $$\begin{aligned}&\int _{r^\star _{3M}}^{\frac{\pi }{2}}\left(\left|R''_{\textrm{QM},\ell } - \omega _\ell ^2 R_{\textrm{QM},\ell }\right|^2 + 4 |\omega _{\ell }|^2\left|R'_{\textrm{QM},\ell }\right|^2\right)\,{\textrm{d}}r^\star \nonumber \\&\quad = \int _{r^\star _{3M}}^{\frac{\pi }{2}} V^2 |R_{\textrm{QM},\ell }|^2\,{\textrm{d}}r^\star + 8\omega _\ell ^2 \int _{r^\star _{3M}}^{\frac{\pi }{2}} \left|R'_{\textrm{QM},\ell }\right|^2\,{\textrm{d}}r^\star \nonumber \\&\qquad + 4\omega _\ell ^2\left(\frac{6Mk^2}{\ell (\ell +1)-2}-\frac{12M\omega _\ell ^2}{\ell (\ell +1)(\ell (\ell +1)-2)}\right)\left|R_{\textrm{QM},\ell }\left(\frac{\pi }{2}\right)\right|^2\nonumber \\&\quad \simeq _{M,k} \left\Vert\widetilde{\Psi }^R_{\textrm{QM},m,\ell }\right\Vert^2_{H^2(\Sigma _{t^\star })}, \end{aligned}$$

where we recall from the proof of Theorem 4.1 the definition (4.30) of $\widetilde{\Psi }_{\textrm{QM},m,\ell }^R$. Using the localisation property (4.3) for $\Psi ^R_{\textrm{QM},m,\ell }$ and (4.6)—recalling that $\Psi ^R_{\textrm{QM},m,\ell } = \chi \widetilde{\Psi }_{\textrm{QM},m,\ell }^R$ –, and using estimate (4.24) for $R_{\textrm{QM},\ell }$ (together with (4.2) and (4.9a)), we infer from (5.12) that for $\ell$ sufficiently large

$$\begin{aligned} \begin{aligned}&\int _{r^\star _{3M}}^{\frac{\pi }{2}}\left(\left|(\chi R_{\textrm{QM},\ell })'' - \omega _\ell ^2 (\chi R_{\textrm{QM},\ell })\right|^2 + 4 |\omega _{\ell }|^2\left|(\chi R_{\textrm{QM},\ell })'\right|^2\right)\,{\textrm{d}}r^\star \\&\quad \simeq _{M,k} \left\Vert\Psi ^R_{\textrm{QM},m,\ell }\right\Vert^2_{H^2(\Sigma _{t^\star })}, \end{aligned} \end{aligned}$$

which plugged in (5.8) yields (5.7).

Now, let us conclude the lemma in the “Robin” case. Using (4.6), (5.7), the definition (5.3) of $\widetilde{\alpha }_{\textrm{QM},m,\ell }^{[\pm 2]}$ and the localisation in $r\ge 3M$ property, we have

5.13 $$\begin{aligned}&\ell ^{2n+2}\left\Vert\Psi ^R_{\textrm{QM},m,\ell }\right\Vert^2_{H^1(\Sigma _{t^\star _0})} \nonumber \\&\quad \lesssim _{M,k,n} \ell ^{2n+2}\left\Vert\Psi ^R_{\textrm{QM},m,\ell }\right\Vert^2_{H^1(\Sigma _{t^\star })} \nonumber \\&\quad \lesssim _{M,k,n} \ell ^{2n}\left(\left\Vert{\partial }_{t^\star }^2\Psi ^R_{\textrm{QM},m,\ell }\right\Vert^2_{L^2(\Sigma _{t^\star })} + \left\Vert{\partial }_{t^\star }\Psi ^R_{\textrm{QM},m,\ell }\right\Vert^2_{H^1(\Sigma _{t^\star })}+ \left\Vert\Psi ^R_{\textrm{QM},m,\ell }\right\Vert^2_{H^2(\Sigma _{t^\star })}\right)\nonumber \\&\quad \lesssim _{M,k,n} \ell ^{2n}\int _{\Sigma _{t^\star }} |{\,\underline{L}}{\,\underline{L}}\Psi ^R_{\textrm{QM},m,\ell }|^2 \\&\quad \lesssim _{M,k,n}\textrm{E}^{\mathfrak {T},n}[\widetilde{\alpha }_{\textrm{QM},m,\ell }].\nonumber \end{aligned}$$

The other inequality in (5.6) follows directly from the definition (5.3) of $\widetilde{\alpha }_{\textrm{QM},\ell }^{[\pm 2]}$, (4.5) and (4.6) and this finishes the proof of the desired (5.6). In the Dirichlet case, we observe by (4.2), that

$$\begin{aligned}&\left|{\,\underline{L}}{\,\underline{L}}\left(\Psi ^D_{\textrm{QM},m,\ell }-\frac{12M{\partial }_t}{\mathcal {L}(\mathcal {L}-2)}\Psi ^D_{\textrm{QM},m,\ell }\right)\right|\\&\quad \simeq _{M,k} \left(1\pm \frac{|\omega _\ell |}{\ell ^4}\right)\left|{\,\underline{L}}{\,\underline{L}}\Psi ^D_{\textrm{QM},m,\ell }\right| \simeq _{M,k} \left(1\pm \ell ^{-3}\right)\left|{\,\underline{L}}{\,\underline{L}}\Psi ^D_{\textrm{QM},m,\ell }\right| \\&\quad \simeq \left|{\,\underline{L}}{\,\underline{L}}\Psi ^D_{\textrm{QM},m,\ell }\right|, \end{aligned}$$

which can be plugged in (5.13) to conclude the proof. $\square$

### Concluding the proof of Theorem 3.1

Let us fix $n>2$ and $p\in \mathbb {N}$. First note that for all $a,b\in {\mathbb R}$,

5.14 $$\begin{aligned} \frac{1}{2}a^2-b^2&\le (a+b)^2 \le 2a^2+2b^2. \end{aligned}$$

In this section we use the quasimodes $\widetilde{\alpha }_{\textrm{QM},m,\ell }^{[\pm 2]}$ and the errors $\widetilde{\alpha }_{{\textrm{Err}},m,\ell }^{[\pm 2]}$ constructed in Sects. 4 and 5. See (5.3) for a definition in terms of the Regge–Wheeler quasimodes and error terms constructed in Theorem 4.1. We will take $m=0$ for simplicity. Using (5.4), (5.5) to control the error terms and (5.6), and using (5.14), we have^14^

5.15 $$\begin{aligned} \begin{aligned}&\frac{\textrm{E}^{\mathfrak {T},n}[\widetilde{\alpha }_{\textrm{QM},0,\ell } + \widetilde{\alpha }_{{\textrm{Err}},0,\ell }](t^\star )}{\textrm{E}^{\mathfrak {T},n}[\mathcal {L}^{p/2}\left(\widetilde{\alpha }_{\textrm{QM},0,\ell } + \widetilde{\alpha }_{{\textrm{Err}},0,\ell }\right)](t^\star _0)} \\&\quad \ge \frac{\textrm{E}^{\mathfrak {T},n}_{r\ge 3M}\left[\widetilde{\alpha }_{\textrm{QM},0,\ell } + \widetilde{\alpha }_{{\textrm{Err}},0,\ell }\right](t^\star )}{\textrm{E}^{\mathfrak {T},n}[\mathcal {L}^{p/2}\left(\widetilde{\alpha }_{\textrm{QM},0,\ell } + \widetilde{\alpha }_{{\textrm{Err}},0,\ell }\right)](t^\star _0)} \\&\quad \ge \frac{\frac{1}{2}\textrm{E}^{\mathfrak {T},n}[\widetilde{\alpha }_{\textrm{QM},0,\ell }](t^\star ) - \textrm{E}^{\mathfrak {T},n}_{r\ge 3M}[\widetilde{\alpha }_{{\textrm{Err}},0,\ell }](t^\star )}{2\textrm{E}^{\mathfrak {T},n}[\mathcal {L}^{p/2}\widetilde{\alpha }_{\textrm{QM},0,\ell }] + 2\textrm{E}^{\mathfrak {T},n}[\mathcal {L}^{p/2}\widetilde{\alpha }_{{\textrm{Err}},0,\ell }](t^\star _0)} \\&\quad \ge \frac{\frac{1}{2}\textrm{E}^{\mathfrak {T},n}[\widetilde{\alpha }_{\textrm{QM},0,\ell }](t^\star ) - C(1+t^\star )e^{-C^{-1}\ell } \big \Vert \Psi ^{R}_{\textrm{QM},0,\ell }\big \Vert ^2_{H^1(\Sigma _{t^\star _0})}}{2\textrm{E}^{\mathfrak {T},n}[\mathcal {L}^{p/2}\widetilde{\alpha }_{\textrm{QM},0,\ell }] + 2Ce^{-C^{-1}\ell } \big \Vert \Psi ^{R}_{\textrm{QM},0,\ell }\big \Vert _{H^1(\Sigma _{t^\star _0})}^2} \\&\quad \ge \frac{\frac{1}{2}C^{-1}\ell ^{2n+2}\big \Vert \Psi ^{R}_{\textrm{QM},0,\ell }\big \Vert ^2_{H^1(\Sigma _{t^\star _0})} - C(1+t^\star )e^{-C^{-1}\ell } \big \Vert \Psi ^{R}_{\textrm{QM},0,\ell }\big \Vert ^2_{H^1(\Sigma _{t^\star _0})}}{2C\ell ^{2n+2p+2}\big \Vert \Psi ^{R}_{\textrm{QM},0,\ell }\big \Vert ^2_{H^1(\Sigma _{t^\star _0})} + 2Ce^{-C^{-1}\ell } \big \Vert \Psi ^{R}_{\textrm{QM},0,\ell }\big \Vert _{H^1(\Sigma _{t^\star _0})}^2} \\&\quad \ge \frac{1}{2}\frac{\frac{1}{2}C^{-2}\ell ^{2n+2}-(1+t^\star )e^{-C^{-1}\ell }}{\ell ^{2n+2p+2}+e^{-C^{-1}\ell }}, \end{aligned} \end{aligned}$$

for all $\ell \ge \ell _c$ with $\ell _c=\ell _c(M,k,n)\ge \ell _\textrm{QM}$ is a sufficiently large constant, and where $C=C(M,k,n)>0$. Taking $\ell = \lfloor 2 C \log (t^\star -t^\star _0)\rfloor$, using that^15^$\lfloor x \rfloor \sim _{x\rightarrow +\infty } x$ and that

$$\begin{aligned} t^\star e^{-C^{-1}\ell }&= \exp \big (\log t^\star - C^{-1} \lfloor 2C \log (t^\star -t^\star _0)\rfloor \big ) \\&= \exp \big (\log t^\star - 2 \log t^\star + o_{t^\star \rightarrow +\infty }(\log (t^\star )\big ) \xrightarrow {t^\star \rightarrow +\infty } 0, \end{aligned}$$

we have

5.16 $$\begin{aligned} \begin{aligned}&\log (t^\star -t^\star _0)^{2p}\frac{\frac{1}{2}C^{-2}\ell ^{2n+2}-(1+t^\star )e^{-C^{-1}\ell }}{\ell ^{2n+2p+2}+e^{-C^{-1}\ell }} \\&\quad = \; \log (t^\star -t^\star _0)^{2p} \frac{\frac{C^{-2}}{2}\lfloor 2C\log (t^\star -t^\star _0)\rfloor ^{2n+2}+o_{t^\star \rightarrow +\infty }(1)}{\lfloor 2C\log (t^\star -t^\star _0)\rfloor ^{2n+2p+2}+o_{t^\star \rightarrow +\infty }(1)} \\ \xrightarrow {t^\star \rightarrow +\infty }&\; \frac{C^{-2}}{2} (2C)^{-2p}>0. \end{aligned} \end{aligned}$$

From (5.15) and (5.16), we deduce

$$\begin{aligned} \liminf _{t^\star \rightarrow +\infty }\sup _{\widetilde{\alpha }\in \mathcal {S}}\left(\log (t^\star -t^\star _0)^{2p} \frac{\textrm{E}^{\mathfrak {T},n}[\widetilde{\alpha }](t^\star )}{\textrm{E}^{\mathfrak {T},n}[\mathcal {L}^{p/2}\widetilde{\alpha }](t^\star _0)}\right) \ge \frac{C^{-2}}{4} (2C)^{-2p}>0, \end{aligned}$$

where

$$\begin{aligned} \mathcal {S}&:= \big \{\widetilde{\alpha }^{[\pm 2]} \text{ solutions } \text{ of } \text{ the } \text{ Teukolsky } \text{ problem } \text{ satisfying } \text{ the } \\ &\qquad \quad \text{ Teukolsky--Starobinsky } \text{ constraints } \text{(1.3) }\big \} \setminus \{0\}, \end{aligned}$$

which is the desired (3.1). Let us now turn to the proof of (3.2). Define

5.17 $$\begin{aligned} \begin{aligned} \widetilde{\alpha }^{[\pm 2]}&:= \sum _{\ell \ge \ell _\textrm{QM}} c_\ell \left(\widetilde{\alpha }^{[\pm 2]}_{\textrm{QM},0,\ell } + \widetilde{\alpha }^{[\pm 2]}_{{\textrm{Err}},0,\ell }\right), \end{aligned} \end{aligned}$$

with

$$\begin{aligned} c_\ell&:= \left(\textrm{E}^{\mathfrak {T},n}[\mathcal {L}^{p/2}\left(\widetilde{\alpha }_{\textrm{QM},0,\ell } + \widetilde{\alpha }_{{\textrm{Err}},0,\ell }\right)](t^\star _0)\right)^{-1/2} \frac{1}{\sqrt{\ell }\log \ell }. \end{aligned}$$

First, using that the $\left(\widetilde{\alpha }^{[\pm 2]}_{\textrm{QM},0,\ell } + \widetilde{\alpha }^{[\pm 2]}_{{\textrm{Err}},0,\ell }\right)_{\ell \ge \ell _{\textrm{QM}}}$ are orthogonal (see Proposition 2.14) and using (5.4) and (5.6), one has

$$\begin{aligned} \textrm{E}^{\mathfrak {T},n+p}[\widetilde{\alpha }](t^\star _0)&= \sum _{\ell \ge \ell _\textrm{QM}} c_\ell ^2 \textrm{E}^{\mathfrak {T},n+p}[\widetilde{\alpha }_{\textrm{QM},0,\ell } + \widetilde{\alpha }_{{\textrm{Err}},0,\ell }](t^\star _0) \\&\lesssim \sum _{\ell \ge \ell _\textrm{QM}} c_\ell ^2 \textrm{E}^{\mathfrak {T},n}\left[\mathcal {L}^{p/2}\left(\widetilde{\alpha }_{\textrm{QM},0,\ell } + \widetilde{\alpha }_{{\textrm{Err}},0,\ell }\right)\right](t^\star _0) \\&\le \sum _{\ell \ge 2} \frac{1}{\ell (\log \ell )^2} < \infty , \end{aligned}$$

and $\widetilde{\alpha }$ defined by (5.17) is an $H^{n+p}$-regular solution of the Teukolsky problem satisfying the Teukolsky–Starobinsky constraints, as required in Theorem 3.1. Moreover, using (5.15),

5.18 $$\begin{aligned} \begin{aligned} \textrm{E}^{\mathfrak {T},n}[\widetilde{\alpha }](t^\star )&= \sum _{\ell \ge \ell _\textrm{QM}} c_\ell ^2 \textrm{E}^{\mathfrak {T},n}[\widetilde{\alpha }_{\textrm{QM},0,\ell } + \widetilde{\alpha }_{{\textrm{Err}},0,\ell }](t^\star ) \\&\ge \sum _{\ell \ge \ell _c} c_\ell ^2\frac{1}{2}\frac{\frac{1}{2}C^{-2}\ell ^{2n+2}-(1+t^\star )e^{-C^{-1}\ell }}{\ell ^{2n+2p+2}+e^{-C^{-1}\ell }}\\&\quad \times \textrm{E}^{\mathfrak {T},n}[\mathcal {L}^{p/2}\left(\widetilde{\alpha }_{\textrm{QM},0,\ell } + \widetilde{\alpha }_{{\textrm{Err}},0,\ell }\right)](t^\star _0) \\&\ge \sum _{\ell \ge 2C\log (t^\star -t^\star _0)} \frac{1}{\ell (\log \ell )^2} \frac{1}{2}\frac{\frac{1}{2}C^{-2}\ell ^{2n+2}-(1+t^\star )e^{-C^{-1}\ell }}{\ell ^{2n+2p+2}+e^{-C^{-1}\ell }}, \end{aligned} \end{aligned}$$

where $\ell _c(M,k,n)>0$ is the (sufficiently large) constant in (5.15) and provided that $t^\star$ is sufficiently large so that $2C\log (t^\star -t^\star _0) \ge \ell _c$. Using direct comparison and integral comparison for numerical series, we have

5.19 $$\begin{aligned} \begin{aligned}&\sum _{\ell \ge 2C\log (t^\star -t^\star _0)} \frac{1}{\ell (\log \ell )^2} \frac{1}{2}\frac{\frac{1}{2}C^{-2}\ell ^{2n+2}-(1+t^\star )e^{-C^{-1}\ell }}{\ell ^{2n+2p+2}+e^{-C^{-1}\ell }} \\&\qquad \quad \sim _{t^\star \rightarrow +\infty } \sum _{\ell \ge 2C\log (t^\star -t^\star _0)} \frac{1}{4C^2}\frac{1}{\ell ^{1+2p} (\log \ell )^2} \\&\qquad \quad \sim _{t^\star \rightarrow +\infty } {\left\{ \begin{array}{ll} \frac{1}{4C^2} \frac{1}{\log \left(\log (t^\star -t^\star _0)\right)} & \text {if }p=0, \\ \frac{1}{4C^2} \frac{1}{2p} \frac{1}{(2C\log (t^\star -t^\star _0))^{2p}}\frac{1}{\left(\log \left(\log (t^\star -t^\star _0)\right)\right)^2} & \text {if }p\in \mathbb {N}^*. \end{array}\right. } \end{aligned} \end{aligned}$$

Plugging (5.19) in (5.18), we get

$$\begin{aligned} \log (t^\star -t^\star _0)^{2p+\varepsilon } \textrm{E}^{\mathfrak {T},n}[\widetilde{\alpha }](t^\star ) \xrightarrow {t^\star \rightarrow +\infty }&+ \infty , \end{aligned}$$

for all $\varepsilon >0$ and for all $p\in \mathbb {N}$, which is the desired (3.2). This finishes the proof of Theorem 3.1.

#### Remark 5.3

In Theorem 1.11 of [GH24b], the boundedness and decay statements for $\widetilde{\alpha }^{[\pm 2]}$ are formulated with the following energy norms:

5.20 $$\begin{aligned} \textrm{E}^{\mathfrak {T},\mathfrak {R},n}[\widetilde{\alpha }] = \textrm{E}^{\mathfrak {T},n}[\widetilde{\alpha }] + \sum _{i=0}^n\left\Vert{\partial }_t^{n-2-i}\Psi ^R\right\Vert_{H^{i-2}(S_{t^\star ,\infty })}^2 + \textrm{E}^{\mathfrak {R},n-2}\left[\mathcal {L}^{-1}(\mathcal {L}-2)^{-1}{\partial }_t\Psi ^D\right], \end{aligned}$$

where $H^{i-2}(S_{t^\star ,\infty })$ is the regular (fractional) Sobolev space on the sphere at infinity $S_{t^\star ,\infty } = \Sigma _{t^\star }\cap \mathcal {I}$, and where $\textrm{E}^{\mathfrak {R},n-2}\left[\mathcal {L}^{-1}(\mathcal {L}-2)^{-1}{\partial }_t\Psi ^D\right]$ is an energy-norm of $\mathcal {L}^{-1}(\mathcal {L}-2)^{-1}{\partial }_t\Psi ^D$ (see [GH24b, (1.24) and Section 1.4]). For the quasimodes $\widetilde{\alpha }=\widetilde{\alpha }_{\textrm{QM},m,\ell }$ constructed in Sect. 5.1, using (4.2) and a trace estimate, one can absorb the additional terms in (5.20) and we have

5.21 $$\begin{aligned} \begin{aligned} \textrm{E}^{\mathfrak {T},\mathfrak {R},n}[\widetilde{\alpha }_{\textrm{QM},m,\ell }](t^\star ) \lesssim \left( 1 + \ell ^{-3}\right) \textrm{E}^{\mathfrak {T},n}[\widetilde{\alpha }_{\textrm{QM},m,\ell }](t^\star ), \end{aligned} \end{aligned}$$

for all $t^\star \ge t^\star _0$. For the error $\widetilde{\alpha }= \widetilde{\alpha }_{{\textrm{Err}},m,\ell }$ all the norms at initial time $t^\star =t^\star _0$ are controlled by (4.8) and we have

5.22 $$\begin{aligned} \textrm{E}^{\mathfrak {T},\mathfrak {R},n}[\widetilde{\alpha }_{{\textrm{Err}},m,\ell }](t^\star _0) \lesssim e^{-C^{-1}\ell }\textrm{E}^{\mathfrak {T},n}[\widetilde{\alpha }_{\textrm{QM},m,\ell }](t^\star _0), \end{aligned}$$

with $C=C(M,k,n)>0$. Hence, combining (5.21) and (5.22), we have $\textrm{E}^{\mathfrak {T},\mathfrak {R},n}[\widetilde{\alpha }](t^\star _0) \simeq \textrm{E}^{\mathfrak {T},n}[\widetilde{\alpha }](t^\star _0)$ for $\widetilde{\alpha }= \widetilde{\alpha }_{\textrm{QM},m,\ell } + \widetilde{\alpha }_{{\textrm{Err}},m,\ell }$, provided that $\ell$ is sufficiently large. It follows that the estimate of Theorem 3.1 also holds with the energies $\textrm{E}^{\mathfrak {T},\mathfrak {R},n}[\widetilde{\alpha }]$ replacing $\textrm{E}^{\mathfrak {T},n}[\widetilde{\alpha }]$ and hence that the main decay estimate (1.24) in [GH24b] is optimal.

## Data Availability

Data sharing is not applicable to this article as no datasets were generated or analysed during the current study.

## The “Metric Reconstruction” Method

In Section 3 of [GH24a] we provided a way to construct a solution of the system of gravitational perturbations in double null gauge from a solution to the Teukolsky system. In this appendix, we provide an alternative way to produce such a solution from the “metric reconstruction” method. While remarkably simple, one of the drawbacks of the method—besides the fact that it is very fragile to non-linear applications—is that it is not clear that *all* solutions of the system of gravitational perturbations arise in this way. In any case, here the method provides a simple way to produce *a* solution exhibiting inverse logarithmic decay.

The Einstein equations $R_{\mu \nu } = \Lambda g_{\mu \nu } = -3k^2 g_{\mu \nu }$ for perturbations $g_{\mu \nu } + \varepsilon h_{\mu \nu }$ of the Schwarzschild-AdS metric $g=g_{\textrm{M,k}}$ linearise as the following *linearised Einstein equations*

A.1 $$\begin{aligned} \begin{aligned} 0&= -\nabla _\mu \nabla _\nu h ^\alpha _\alpha - \nabla ^\alpha \nabla _\alpha h_{\mu \nu } + \nabla ^\alpha \nabla _\nu h_{\alpha \mu } + \nabla ^\alpha \nabla _\mu h_{\alpha \nu } \\ &\quad + g_{\mu \nu }\left(\nabla ^\alpha \nabla _\alpha h^{\beta }_{\beta }- \nabla ^\alpha \nabla ^{\beta }h_{\alpha {\beta }}\right) +2 \Lambda h_{\mu \nu }. \end{aligned} \end{aligned}$$

The following theorem is an adaptation of the “metric reconstruction” of [CK75, Chr75, KC79, Wal78]. See also [DRS09, Appendix C], and see further developments of this idea in [GHZ20, HT24]. The—long but direct—computation is left to the reader.^16^

### Theorem A.1

Let $\widetilde{\alpha }^{[-2]}_G$ be a solution of the $[-2]$-Teukolsky equation (2.5b). Then recalling (1.4) and setting $t=u+v, r^\star =v-u$ (the convention of [GH24a]), the expression

A.2 $$\begin{aligned} \begin{aligned} \mathfrak {h}[\widetilde{\alpha }_G^{[-2]}]&= -4\textrm{Re}\bigg [2\mathcal {L}_2 (w^{-1}L)\left(r\widetilde{\alpha }^{[-2]}_G\right) \left({\textrm{d}}u ({\textrm{d}}{\vartheta }+i\sin {\vartheta }{\textrm{d}}\varphi ) \right. \\&\qquad \left. +({\textrm{d}}{\vartheta }+i\sin {\vartheta }{\textrm{d}}\varphi ){\textrm{d}}u\right) + 4\mathcal {L}_1\mathcal {L}_2\left(r\widetilde{\alpha }_G^{[-2]}\right){\textrm{d}}u {\textrm{d}}u \\&\qquad + r^{-1}(w^{-1}L)\left(r^2(w^{-1}L)\widetilde{\alpha }_G^{[-2]}\right)({\textrm{d}}{\vartheta }+i\sin {\vartheta }{\textrm{d}}\varphi )^2\bigg ], \\ \end{aligned} \end{aligned}$$

defines a real, symmetric, spacetime 2-tensor, solution to the linearised Einstein equations (A.1). Moreover, its associated Teukolsky quantities $\widetilde{\alpha }^{[\pm 2]}[\mathfrak {h}[\widetilde{\alpha }_G^{[-2]}]]$ are given by

A.3 $$\begin{aligned} \widetilde{\alpha }^{[+2]}[\mathfrak {h}[\widetilde{\alpha }_G^{[-2]}]]&= w^2(w^{-1}L)^4(\widetilde{\alpha }_G^{[-2]})^*, \nonumber \\ \widetilde{\alpha }^{[-2]}[\mathfrak {h}[\widetilde{\alpha }_G^{[-2]}]]&= \mathcal {L}^{\dagger }_{-1}\mathcal {L}^{\dagger }_0\mathcal {L}^{\dagger }_{1}\mathcal {L}^{\dagger }_{2}(\widetilde{\alpha }_G^{[-2]})^*- 12M{\partial }_t\widetilde{\alpha }_G^{[-2]}. \end{aligned}$$

### Remark A.2

If $\widetilde{\alpha }^{[\pm 2]}_G$ are solutions to the Teukolsky problem (cf. Definition 2.1) satisfying in addition the Teukolsky–Starobinsky relations (1.3), then

A.4 $$\begin{aligned} \begin{aligned} \widetilde{\alpha }^{[+2]}[\mathfrak {h}[\widetilde{\alpha }_G^{[-2]}]]&= \mathcal {L}_{-1}\mathcal {L}_0\mathcal {L}_{1}\mathcal {L}_{2}(\widetilde{\alpha }_G^{[+2]})^*- 12M{\partial }_t\widetilde{\alpha }_G^{[+2]},\\ \widetilde{\alpha }^{[-2]}[\mathfrak {h}[\widetilde{\alpha }_G^{[-2]}]]&= \mathcal {L}^{\dagger }_{-1}\mathcal {L}^{\dagger }_0\mathcal {L}^{\dagger }_{1}\mathcal {L}^{\dagger }_{2}(\widetilde{\alpha }_G^{[-2]})^*- 12M{\partial }_t\widetilde{\alpha }_G^{[-2]}. \end{aligned} \end{aligned}$$

In particular, $\widetilde{\alpha }^{[\pm 2]}[\mathfrak {h}[\widetilde{\alpha }_G^{[-2]}]]$ are also solutions to the Teukolsky problem.

### Remark A.3

The tensor $\mathfrak {h}$ given by (A.2) is in the so-called *ingoing radiation gauge*, *i.e.* $\mathfrak {h}({\,\underline{L}},\cdot )=0$. Defining

$$\begin{aligned} \widetilde{\mathfrak {h}}_{\mu \nu }&:= \mathfrak {h}_{\mu \nu } + \nabla _\mu (fL)_\nu + \nabla _\nu (fL)_\mu \\ &\; = \big [\mathfrak {h} -2\Omega ^2{\textrm{d}}f {\textrm{d}}u - 2\Omega ^2{\textrm{d}}u {\textrm{d}}f +2\Omega ^2f r({\textrm{d}}{\vartheta }^2+\sin ^2{\vartheta }{\textrm{d}}\varphi ^2)\big ]_{\mu \nu }, \end{aligned}$$

with ${\,\underline{L}}f = -4\Omega ^{-2}\mathcal {L}_1\mathcal {L}_2(r\widetilde{\alpha }^{[-2]}_G)$, and $f = 0$ on $\mathcal {I}$, we have that $\widetilde{\mathfrak {h}}$ is in the *double null gauge* of [GH24a] and still satisfies all the conclusions of Theorem A.1. One can observe that $\widetilde{\alpha }^{[\pm 2]}_G$ being regular at the future horizon and at infinity implies that $\widetilde{\mathfrak {h}}$ is regular at the horizon and asymptotically AdS in the sense of [GH24a, (48) and Definition 2.6].^17^ Moreover, one can easily see that $\widetilde{\alpha }^{[\pm 2]}_G$ satisfying the boundary conditions (1.2) implies that $\mathfrak {h}$ is conformal to the induced Anti-de Sitter metric at infinity in the sense of [GH24a, Definition 2.4].^18^

### Remark A.4

To obtain a proof of Theorem 1.4 with the metric reconstruction method, one still has to show that all solutions to the Teukolsky problem, satisfying the Teukolsky–Starobinsky identities, can be obtained as the Teukolsky quantities of $\mathfrak {h}$, *i.e.* that the map $\widetilde{\alpha }^{[\pm 2]}_G \mapsto \widetilde{\alpha }^{[\pm 2]}[\mathfrak {h}[\widetilde{\alpha }_G^{[-2]}]]$ given by (A.4) is surjective. We believe that in the present Schwarzschild-AdS case, the map is indeed surjective. See [WP05, AAB19] for discussions, reviews and results in the asymptotically flat Kerr case.

## The Teukolsky IBVP with Teukolsky–Starobinsky Constraints

In this appendix we connect to previous results in the physics literature where the Teukolsky system for $\widetilde{\alpha }^{[+2]}$ and $\widetilde{\alpha }^{[-2]}$ was decoupled at the expense of a higher order boundary condition. We first recall this result, which in our case is a straightforward consequence of Proposition 2.21.

### Corollary B.1

Let $\widetilde{\alpha }^{[+2]}$ and $\widetilde{\alpha }^{[-2]}$ be solutions to the Teukolsky problem as in Definition 2.1, which in addition satisfy the Teukolsky–Starobinsky identities (1.3). Then

- $\widetilde{\alpha }^{[+2]}$ satisfies the decoupled higher order boundary conditions
  B.1a $$\begin{aligned} k^2\mathcal {L}^{[+2]} {\,\underline{L}}\widetilde{\alpha }^{[+2]}_s + {\partial }_t{\,\underline{L}}{\,\underline{L}}\widetilde{\alpha }^{[+2]}_s&\xrightarrow {r\rightarrow +\infty } 0, \end{aligned}$$
  B.1b $$\begin{aligned} {\,\underline{L}}{\,\underline{L}}\widetilde{\alpha }^{[+2]}_a&\xrightarrow {r\rightarrow +\infty } 0 . \end{aligned}$$
- $\widetilde{\alpha }^{[-2]}$ satisfies the higher order decoupled boundary conditions
  B.2a $$\begin{aligned} k^2\mathcal {L}^{[-2]}L\widetilde{\alpha }^{[-2]}_s+{\partial }_tLL\widetilde{\alpha }^{[-2]}_s&\xrightarrow {r\rightarrow +\infty } 0, \end{aligned}$$
  B.2b $$\begin{aligned} LL\widetilde{\alpha }^{[-2]}_a&\xrightarrow {r\rightarrow +\infty } 0. \end{aligned}$$

### Proof

We have

$$\begin{aligned} \text {(B.1a) and~(B.2a)} \Leftrightarrow \Psi ^{[\pm 2]}_a \xrightarrow {r\rightarrow +\infty } 0 \Leftrightarrow \Psi ^R_a \xrightarrow {r\rightarrow +\infty } 0 \text {\ and \ } r^2 \partial _r \Psi ^R_a \xrightarrow {r\rightarrow +\infty } 0 \Leftrightarrow \Psi ^R_a= 0, \end{aligned}$$

where the first equivalence follows from the definition of $\Psi ^{[\pm 2]}_a$, the second from combining (2.35) and (2.36bb) and the last equivalence follows from unique continuation (see footnote 8). Furthermore, using equations (2.14) and the boundary conditions (2.11), (1.7a),

$$\begin{aligned} \text {(B.1a) and~(B.2a)}&\Leftrightarrow \mathcal {L}\left(\psi ^{[+2]}_s+\psi ^{[-2]}_s\right) + {\partial }_t\Psi ^{[+2]}_s + {\partial }_t\Psi ^{[-2]}_s \xrightarrow {r\rightarrow +\infty } 0 \\&\Leftrightarrow -\left(L\Psi ^{[+2]}_s + {\,\underline{L}}\Psi ^{[-2]}_s\right) + {\partial }_t\Psi ^{[+2]}_s + {\partial }_t\Psi ^{[-2]}_s \xrightarrow {r\rightarrow +\infty } 0 \\&\Leftrightarrow {\partial }_{r^\star }\Psi ^D_s \xrightarrow {r\rightarrow +\infty } 0 \Leftrightarrow \Psi ^D_s = 0. \end{aligned}$$

The corollary then follows from Proposition 2.21. $\square$

### Remark B.2

To our knowledge, the type of higher order boundary conditions (B.1) and (B.2) for Teukolsky quantities first appeared in the case of mode solutions on Kerr-AdS in [DS13] (see also a derivation in [GH23, Appendix A]). It was used in [DS13, CDH14] and further works to compute the mode solutions to each of the Teukolsky equation alone, thereby avoiding to consider a system of coupled equations.

Given the unusual form of the boundary conditions (B.1) and (B.2) it is not clear whether the Teukolsky equation for $\widetilde{\alpha }^{[+2]}$ or $\widetilde{\alpha }^{[-2]}$ with the boundary conditions (B.1) or (B.2) gives rise in itself to a well-posed evolution problem; see the discussion in [HLSW20, §6.3]. To construct solutions to the decoupled initial boundary value problem for $\widetilde{\alpha }^{[+2]}$ (or for $\widetilde{\alpha }^{[-2]}$) satisfying the higher order boundary conditions of Corollary B.1, one can construct an auxiliary $\widetilde{\alpha }^{[-2]}$ (or $\widetilde{\alpha }^{[+2]}$ resp.) and apply the usual well-posedness result for the coupled system. From this point of view there is no gain in having the decoupled higher order formulation. We nevertheless state the result and its proof.

### Theorem B.3

(Well-posedness of the Teukolsky IBVP with Teukolsky–Starobinsky constraints). Let $t^\star _0\in \mathbb {R}$ and let $\widetilde{\alpha }^{[+2]}_0,\dot{\widetilde{\alpha }}^{[+2]}_0$ be two smooth spin-$+2$-weighted complex functions on $\Sigma _{t^\star _0}$, regular at the horizon and at infinity and satisfying the corner compatibility condition on the corner $\Sigma _{t^\star _0}\cap \mathcal {I}$

B.3 $$\begin{aligned} \mathcal {L}^{[+2]}\psi ^{[+2]}_{0,s} + {\partial }_t\Psi ^{[+2]}_{0,s}&\xrightarrow {r\rightarrow +\infty } 0, \end{aligned}$$

B.4 $$\begin{aligned} {\partial }_t^n\Psi ^{[+2]}_{0,a}&\xrightarrow {r\rightarrow +\infty } 0, \end{aligned}$$

for all $n\ge 0$, where $\psi _{0}^{[+2]} := w^{-1}(\dot{\widetilde{\alpha }}^{[+2]}_0 - {\partial }_{r^\star }\widetilde{\alpha }^{[+2]})$, and where $\Psi _0^{[+2]}$ and its time derivatives are defined in terms of $\widetilde{\alpha }^{[+2]}_0,\dot{\widetilde{\alpha }}^{[+2]}_0$ consistently with the Teukolsky equation (2.5a) being satisfied. There exists a unique smooth spin-$+2$-weighted function $\widetilde{\alpha }^{[+2]}$ on $\{t^\star \ge t^\star _0\}$, solution to the Teukolsky equation (2.5a), regular at the horizon and at infinity, and such that the boundary condition (B.1) holds, *i.e.*

$$\begin{aligned} k^2\mathcal {L}^{[+2]} {\,\underline{L}}\widetilde{\alpha }^{[+2]}_s + {\partial }_t{\,\underline{L}}{\,\underline{L}}\widetilde{\alpha }^{[+2]}_s \xrightarrow {r\rightarrow +\infty } 0, &  \text {and} &  {\,\underline{L}}{\,\underline{L}}\widetilde{\alpha }^{[+2]}_a \xrightarrow {r\rightarrow +\infty } 0. \end{aligned}$$

Moreover, there exists a unique smooth spin-$-2$-weighted function $\widetilde{\alpha }^{[-2]}$ on $\{t^\star \ge t^\star _0\}$ solution of the Teukolsky equation (2.5b), regular at the horizon and at infinity, and such that the couple $(\widetilde{\alpha }^{[+2]},\widetilde{\alpha }^{[-2]})$ satisfy the conformal Anti-de Sitter boundary conditions (1.2) and the Teukolsky–Starobinsky constraints (1.3). For all $n\ge 3$ and all $t^\star _1\ge t^\star _0$, we have the estimates

B.5 $$\begin{aligned} \begin{aligned} \textrm{E}^{\mathfrak {T},n}[\widetilde{\alpha }](t^\star _1)&\lesssim _{M,k,n} \textrm{E}^{\mathfrak {T},n}[\widetilde{\alpha }](t^\star _0). \end{aligned} \end{aligned}$$

The analogous result holds *mutatis mutandis* with $\widetilde{\alpha }^{[\mp 2]}$ instead of $\widetilde{\alpha }^{[\pm 2]}$.

### Proof

We start with the $[-2]$ case. Let $\widetilde{\alpha }^{[-2]}_{\textrm{IVP}}$ be the solution of the classical IVP for the Teukolsky equation (2.5b) on $D^+(\Sigma _{t^\star _0})$. Define ${\underline{\mathcal {C}}}:= \{t+r^\star = t^\star _0 + \pi /2\}$, the ingoing null cone emanating from the corner $\Sigma _{t^\star _0}\cap \mathcal {I}$. On ${\underline{\mathcal {C}}}$ we know $\widetilde{\alpha }^{[-2]}=\widetilde{\alpha }^{[-2]}_{\textrm{IVP}}$ and all its derivatives and our goal is to construct $\widetilde{\alpha }^{[+2]}$ (and all its derivatives). At the corner ${\underline{\mathcal {C}}}\cap \mathcal {I}$, we have $\widetilde{\alpha }^{[+2]}=(\widetilde{\alpha }^{[-2]})^*$ by the Teukolsky boundary condition (1.2a). Moreover, for all $n\ge 1$, $(w^{-1}{\,\underline{L}})^n\widetilde{\alpha }^{[+2]}$ is determined by $\widetilde{\alpha }^{[-2]}$ and its derivatives (see the proof of Proposition 2.2, more precisely (1.2b), (1.7a), (2.18)). The quantities $\widetilde{\alpha }^{[\pm 2]}$ will have to satisfy the Teukolsky–Starobinsky identity (1.3b), which we rewrite for convenience

B.6 $$\begin{aligned} w^2(w^{-1}{\,\underline{L}})^4(\widetilde{\alpha }^{[+2]})^*&= \mathcal {L}_{-1}^{\dagger }\mathcal {L}_{0}^{\dagger }\mathcal {L}_{1}^{\dagger }\mathcal {L}_{2}^{\dagger }(\widetilde{\alpha }^{[-2]})^*+ 12M{\partial }_t\widetilde{\alpha }^{[-2]}. \end{aligned}$$

Since the right-hand side of (B.6) is known on ${\underline{\mathcal {C}}}$, we define $\widetilde{\alpha }^{[+2]}$ as the solution of (B.6)—seen as four transport equations along ${\,\underline{L}}$—-with initial conditions at the corner given by (1.2a), (1.2b), (1.7a), (2.18). All the transversal derivatives of $\widetilde{\alpha }^{[+2]}$ can then be determined by the fact that $\widetilde{\alpha }^{[+2]}$ must satisfy the Teukolsky equation (2.5a) and using expressions of $(w^{-1}L)^n\widetilde{\alpha }^{[+2]}$ in terms of derivatives of $\widetilde{\alpha }^{[-2]}$. Note moreover that with this definition $\widetilde{\alpha }^{[-2]}$ being regular at the horizon implies that $\widetilde{\alpha }^{[+2]}$ is regular at the future horizon. We can now define $(\widetilde{\alpha }^{[+2]},\widetilde{\alpha }^{[-2]})$ to be the solution on the domain $\mathcal {D}:= D^+\left({\underline{\mathcal {C}}}\cup \mathcal {I}\right)$ of the system of Teukolsky equations (2.5) with boundary conditions (1.2) which is given by a classical well-posedness result (see Theorem 1.6 in [GH24a]).

We want to check that the Teukolsky–Starobinsky identities (1.3) hold on $\mathcal {D}$, which is equivalent to $\Psi ^D_s=\Psi ^R_a=0$ by the result of Proposition 2.21. First note that (B.6) implies

B.7 $$\begin{aligned} (w^{-1}{\,\underline{L}})^2\Psi ^D_s&= (w^{-1}{\,\underline{L}})^2\Psi ^R_a = 0 \quad \quad \text {on }{\underline{\mathcal {C}}}. \end{aligned}$$

The corner conditions (B.3), (B.4), in the $[-2]$ case write as

B.8 $$\begin{aligned} \mathcal {L}^{[-2]}\psi ^{[-2]}_{s} + {\partial }_t\Psi ^{[-2]}_{s}&\xrightarrow {r\rightarrow +\infty } 0, \end{aligned}$$

B.9 $$\begin{aligned} {\partial }_t^n\Psi ^{[-2]}_{a}&\xrightarrow {r\rightarrow +\infty } 0, \end{aligned}$$

on ${\underline{\mathcal {C}}}\cap \mathcal {I}$ and for all $n\ge 0$. Since $\widetilde{\alpha }^{[+2]},\widetilde{\alpha }^{[-2]}$ satisfy the Teukolsky equations (2.5) and the boundary conditions (1.2), the limit (2.19) of the proof of Proposition 2.2 holds and, using (B.8) and its equivalent for the $[+2]$ quantities obtained using (1.2b), (1.7a), one deduces that ${\,\underline{L}}\Psi ^D_s\rightarrow 0$ at the corner ${\underline{\mathcal {C}}}\cap \mathcal {I}$. From that limit and the fact that $\Psi ^D_s\rightarrow 0$ at $\mathcal {I}$, we deduce by integrating two times (B.7) that $\Psi ^D_s=0$ on ${\underline{\mathcal {C}}}$. By energy estimate, it follows that $\Psi ^D_s=0$ on $\mathcal {D}$ (see [GH24b]). Now, (B.9) together with (1.7a) implies that $\Psi ^R_a,{\partial }_t\Psi ^R_a,{\partial }^2_t\Psi ^R_a\rightarrow 0$ at the corner ${\underline{\mathcal {C}}}\cap \mathcal {I}$, which together with the “Robin” condition (2.36bb), also implies that ${\,\underline{L}}\Psi ^R_a\rightarrow 0$ at ${\underline{\mathcal {C}}}\cap \mathcal {I}$. Arguing as for $\Psi ^D_s$, we conclude that $\Psi ^R_a=0$ on $\mathcal {D}$, hence the Teukolsky–Starobinsky identities are satisfied by $\widetilde{\alpha }^{[+2]},\widetilde{\alpha }^{[-2]}$. By Corollary B.1, we infer that $\widetilde{\alpha }^{[-2]}$ satisfies the desired boundary conditions (B.2). It remains to extend $\widetilde{\alpha }^{[+2]}$ to the bottom part $D^+(\Sigma _{t^\star _0})$. This can be done by integrating Teukolsky–Starobinsky identity (1.3a)—seen as a first order ODE along ${\partial }_t$ for $\widetilde{\alpha }^{[+2]}$—backwards from ${\underline{\mathcal {C}}}$ into $D^+(\Sigma _{t^\star _0})$. We leave to the reader to check that, by integration from ${\underline{\mathcal {C}}}$, $\widetilde{\alpha }^{[+2]}$ satisfies all the desired properties in $D^+(\Sigma _{t^\star _0})$. By the red-shift and energy estimates of [GH24b], we have that (B.5) holds for $\widetilde{\alpha }^{[+2]},\widetilde{\alpha }^{[-2]}$ and this finishes the proof of the theorem in the $[-2]$ case.

Let us now sketch the $[+2]$ case. The $[+2]$ case is less natural since the Teukolsky–Starobinsky identities (1.3) do not give obvious transport equations for $\widetilde{\alpha }^{[-2]}$ along ${\underline{\mathcal {C}}}$ with $\widetilde{\alpha }^{[+2]}$ as source terms. Nonetheless, one can still define $(\Psi ^{[-2]}_s)^*= \Psi ^{[+2]}_s$ on ${\underline{\mathcal {C}}}$, and $\Psi ^{[-2]}_a$ as the solution—with the appropriate boundary conditions given by (1.7a), (2.18)—of $w^2(w^{-1}L)^2(\Psi ^{[-2]}_a)^*= -(\mathcal {L}(\mathcal {L}-2)+12M{\partial }_t)\widetilde{\alpha }^{[+2]}_a$ on ${\underline{\mathcal {C}}}$, where we replace the *L* by ${\,\underline{L}}$ on the LHS using that $(\mathcal {L}(\mathcal {L}-2)-6M(L+{\,\underline{L}}))(\Psi ^{[-2]}_a)^*= - (\mathcal {L}(\mathcal {L}-2)+12M{\partial }_t)\Psi ^{[+2]}_a$ (since $\Psi ^R_a$ must vanish). Now that $\Psi ^{[-2]}$ is defined on ${\underline{\mathcal {C}}}$, one can define $\widetilde{\alpha }^{[-2]},\psi ^{[-2]}$ using that (2.14b) gives an expression for $\widetilde{\alpha }^{[-2]}$ in terms of $\psi ^{[-2]},\Psi ^{[-2]}$, which can be plugged in (2.12b) to yield a transport equation for $\psi ^{[-2]}$ along ${\underline{\mathcal {C}}}$. The rest of the proof then follows along analogous ideas as in the $[-2]$ case and is left to the reader. This finishes the proof of the theorem. $\square$

### Remark B.4

Theorem B.3 could be formulated for initial data posed on an ingoing null hypersurface ${\underline{\mathcal {C}}}$. In that case, following the proof of the theorem, the initial data would be a triplet $(\widetilde{\alpha }; L\widetilde{\alpha }, L^2\widetilde{\alpha })$ where $\widetilde{\alpha }$ is one of the Teukolsky quantities $\widetilde{\alpha }^{[\pm 2]}$ on ${\underline{\mathcal {C}}}$, and $L\widetilde{\alpha },L^2\widetilde{\alpha }$ are its transverse derivatives at the corner ${\underline{\mathcal {C}}}\cap \mathcal {I}$. Theorem B.3 is then clearly a Teukolsky version of the well-posedness result for the full system of gravitational perturbations [GH24a, Theorem 3.9] (and could actually be obtained, in the $[-2]$ case, as a corollary of that theorem). In fact the proof of Theorem B.3 follows the same idea as  [GH24a, Theorem 3.9]: constructing initial data for all the quantities using the constraints, then applying a standard well-posedness result for wave equations.

## Footnotes

1 In particular, the Teukolsky quantities associated with a solution of the system of gravitational perturbations will always satisfy these relations (showing immediately the necessity of the condition). See Lemma 2.15 of [GH24a].

2 For the wave equation, one can in fact construct a large class of solutions which decay exponentially in time by a scattering construction [Hoo24]. In view of the robustness of that construction, there should be no serious issue in generalising it to the Teukolsky system studied here.

3 Subject to additional conditions on the black hole parameters, specifically the validity of the Hawking-Reall bound.

4 At the mode level, these result in a Robin-type boundary condition for (the radial part of) each mode, which allows relatively straightforward numerical computation of the mode.

5 The elliptic operator $\mathcal {L}(\mathcal {L}-2)$ is invertible. See Sect. 2.8.1 for further discussion.

6 For mode solutions, the eigenvalues of $\mathcal {L}(\mathcal {L}-2)-12M{\partial }_t$ and $\mathcal {L}(\mathcal {L}-2)+12M{\partial }_t$ coincide with the so-called “radial Teukolsky–Starobinsky constants”, see *e.g.* [Cha83]. These operators also appear in the so-called Teukolsky–Starobinsky identities which relate $\widetilde{\alpha }^{[+2]}$ and $\widetilde{\alpha }^{[-2]}$, see Sect. 2.8.

7 Note that $$(\mathcal {C}\phi )^*= \mathcal {C}^*\phi ^*$$.

8 More precisely, we use the fact that if a solution to the Teukolsky system satisfies both Dirichlet and Neumann conditions on both quantities at the conformal boundary, then the solution necessarily vanishes. While one could invoke general unique continuation results (see for instance Section 6 of [HS16] where the Teukolsky system is discussed on pure AdS), in our simple spherically symmetric setting one could decompose the solution into spherical modes to produce a $1+1$-PDE system for which local energy estimates imply the result. .

9 As it will be clear in the proof, neither the frequencies $\omega ^D_{\ell }, \omega ^R_{\ell }$, nor the functions $R^D_{\textrm{QM},\ell },R^R_{\textrm{QM},\ell }$, depend on *m*.

10 We recall that $H^1_0\left((r^\star _{3M},\pi /2]\right)$ is the closure for the standard $H^1$-norm of the space of smooth and compactly supported functions with compact support included in the interval $(r^\star _{3M},\pi /2]$ (in particular, these functions vanish at $r^\star _{3M}$ but can take any value at $\pi /2$).

11 Since we integrate only from $r=3M$ to $r=+\infty$, the potential $w\left(\ell (\ell +1)-\frac{6M}{r}\right)$ is in that case always positive and *a* indeed defines a scalar product.

12 Since, by the spectral theorem, *T* is diagonalisable in a Hilbert basis of $\mathcal {H}$, whose basis vectors must coincide with the $R_{\textrm{QM},\ell ,n}$.

13 Note also the relation

5.1 $$\begin{aligned} \begin{aligned} \Psi _{\textrm{QM},m,\ell }(t,r^\star ,{\vartheta },\varphi )&= e^{-i\omega _\ell t}\chi (r^\star )R_{\textrm{QM},\ell }(r^\star )S_{m\ell }({\vartheta })e^{+im\varphi } + e^{+i\omega _\ell t}\chi (r^\star )R_{\textrm{QM},\ell }(r^\star )S_{-m\ell }({\vartheta })e^{-im\varphi } \\&= e^{-i\omega _\ell t^\star } e^{i\omega _\ell \left(r^\star -\frac{1}{k}\arctan (kr)\right)}\chi (r^\star )R_{\textrm{QM},\ell }(r^\star )S_{m\ell }({\vartheta })e^{+im\varphi } \\&\quad +e^{+i\omega _\ell t^\star } e^{-i\omega _\ell \left(r^\star -\frac{1}{k}\arctan (kr)\right)}\chi (r^\star )R_{\textrm{QM},\ell }(r^\star )S_{-m\ell }({\vartheta })e^{-im\varphi }. \end{aligned} \end{aligned}$$

to convert to the $(t^\star ,r^\star ,\theta ,\phi )$ coordinates used to express the Sobolev norms. One could of course have avoided this (trivial) conversion altogether by defining $t^\star$ to agree with *t* in $r\ge r_+ + \frac{3M-r_+}{2}$ as the solutions constructed in this paper are supported in $r \ge 3M-\delta$ only.

14 All the estimates (5.4), (5.5) and (5.6) hold for all constant $\widetilde{C}$ with $\widetilde{C}>C$ where *C* is the respective constant in these estimates. Thus, up to taking the maximum of all the constants *C* we can assume that *C* is the same in (5.4), (5.5) and (5.6).

15 Here and in the sequel we write $f(x)\sim _{x\rightarrow +\infty }g(x)$ for “ $f(x)/g(x)\rightarrow 1$ when $x\rightarrow +\infty$” and $f(x)=o_{x\rightarrow +\infty }(g(x))$ for “ $f(x)/g(x)\rightarrow 0$ when $x\rightarrow +\infty$”.
